# PERSonality, Ehical, and PROfessional quality of life in Pediatric/Adult Intensive Nurses study: PERSEPRO PAIN study

**DOI:** 10.1371/journal.pone.0259721

**Published:** 2022-03-07

**Authors:** Yujiro Matsuishi, Bryan J. Mathis, Haruhiko Hoshino, Yuki Enomoto, Nobutake Shimojo, Satoru Kawano, Hideaki Sakuramoto, Yoshiaki Inoue

**Affiliations:** 1 Neuroscience Nursing, St. Luke’s International University, Tokyo, Japan; 2 International Medical Center, University of Tsukuba Hospital, Tsukuba, Ibaraki, Japan; 3 Adult Health Nursing, Department of Nursing, International University of Health and Welfare, Narita, Japan; 4 Department of Pediatrics, University of Tsukuba Hospital, Tsukuba, Ibaraki, Japan; 5 Department of Emergency and Critical Care Medicine, Faculty of Medicine, University of Tsukuba, Tsukuba, Ibaraki, Japan; 6 Adult Health Nursing, College of Nursing, Ibaraki Christian University, Hitachi, Ibaraki, Japan; Kaohsuing Medical University Hospital, TAIWAN

## Abstract

**Background:**

The World Health Organization included burnout syndrome criteria that reduce both professional quality of life and work satisfaction in its 11th Revision of the International Classification of Diseases in 2019 while nursing bodies have issued action calls to prevent burnout syndrome. Despite this, the effect of social factors, personality traits and cross-interaction on professional quality of life is still unclear.

**Aim:**

To reveal the association between ethical climate, personal trait and professional quality of life.

**Method:**

An online survey of registered nurses working in adult, pediatric or both ICUs. We used the ten-item personality measure based on The Big Five theory and Type-D personality Scale-14 then measured the ethical climate with the Hospital Ethical Climate Survey and the professional domains of burnout syndrome, secondary traumatic stress and compassion satisfaction by the Professional Quality of Life Scale Version 5 simultaneously. Multivariate analysis confirmed the triangular association of hospital ethical climate, personality traits and professional quality of life.

**Result:**

We enrolled 310 participants from September 2019 to February 2020. Mean age was 33.1 years (± 5.9) and about 70% were female. In the multivariate analysis, neuroticism (p = 0.03, p = 0.01) and Type D personality (both of p<0.01) were associated with burnout syndrome and secondary traumatic stress while agreeableness (p<0.01) was associated with secondary traumatic stress. Conversely, extraversion (p = 0.01), agreeableness (p<0.01) and openness (p<0.01) were associated with compassion satisfaction. We also observed interactions between hospital ethical climate and conscientiousness (p = 0.01) for burnout syndrome and secondary traumatic stress. Neuroticism was related to (p<0.01) BOS and compassion satisfaction while Type D personality (p<0.01) correlated with burnout syndrome and secondary traumatic stress.

**Conclusion:**

Hospital ethical climate strongly affects professional quality of life in nurses with specific personality traits. Therefore, it is important to maintain an ethical hospital climate, considering individual personalities to prevent burnout syndrome.

## Introduction

The term “burnout” was first used in nearly half a century ago by Freudenberger [1] while Maslach defined burnout syndrome (BOS) by three symptoms: emotional exhaustion, depersonalization and reduced personal accomplishment [2, 3]. The World Health Organization (WHO) included BOS criteria in its 11th Revision of the International Classification of Diseases (ICD-11) in 2019 [4] and the UK Health and Safety Executive (HSE) reported 15.4 million working days lost due to BOS in 2017–201 [5]. BOS in the nursing fields, especially critical care, has been recognized by national societies such as the Critical Care Societies Collaborative (CCSC) and the American Association of Critical Care Nurses (AACN) with calls to action for BOS prevention [6].

Working specialty plays a crucial role in burnout, an effect especially seen in pediatric intensive care [7] and intensive care nurses [8–10]. These nurses have been reported to experience intense occupational stress in their daily work [11] and, as observing death is a strong risk factor for BOS [12], places where death is frequently observed, such as the intensive care unit, makes BOS prevention challenging. The result is that patient safety can be compromised as seen in a previous study which revealed that time pressure adversely affected patient safety for nurses with BOS [13], an association confirmed by another study [14]. From these reasons, further studies on BOS in intensive care nurses are required and these studies benefit both nurses and patients. Previous studies have already confirmed that BOS is related to social factors such as work environment/climate, management [15, 16], and also specific personality factors [17–22].

Recently, BOS has been classified as not merely psychological but also a domain of professional quality of life (QOL). Stamm et al defined professional QOL as the feeling resulting from the relationship between caregivers and patients that includes three domains: burnout (BOS), secondary traumatic stress (STS) and compassion satisfaction(CS) [23].

The word “compassion” describes the sympathetic awareness of another’s distress and STS is compassion fatigue defined as the emotional withdrawal experienced by caregivers of traumatized people. Although it may be an expected side effect of caregiving, nursing is still a high risk cohort for having STS in spite of efforts and training to prevent it [24]. Balancing STS is CS, which is a positive feeling of accomplishment gained through the caregiving profession. Nurses must constantly walk this tightrope between STS and CS and thus measurements of professional QOL [23] must observe BOS, STS and CS simultaneously to analyze the balance between these three domains.

Even with an understanding of the association between BOS and its associated domains (STS and CS), associated factors for professional QOL are still unclear. A recent study revealed that lifestyle and demographic information were associated with a poor professional QOL [25] but the impact and interaction of factors such as hospital ethical climate and personality traits on BOS is underreported.

Therefore, we hypothesize that:

1. The ethical climate of a hospital (hospital ethical climate; HEC) is an independent risk factor for professional QOL (BOS, STS and CS).
2. Specific personality traits are independent risk factors for professional QOL (BOS, STS and CS).
3. There are interactions between hospital ethical climate and specific personality traits for professional QOL, indicating that hospital ethical climate strongly affects professional QOL in nurses with specific personality traits.

## Method

### Participant selection

The inclusion criteria were registered nurses currently working in adult, pediatric or both ICUs in Japan while exclusion criteria were nurses not working in included centers or not working in Japan. We used an online survey to solicit participants and enrolled registered nurses working in adult, pediatric or hybrid ICUs from September 2019 to February 2020. The survey was distributed via the official laboratory social network services (SNS) account to recruit participants. Informed consent (via an online form) was taken before the online survey and ethical approval was obtained (The University of Tsukuba Institutional Review Board: approval # R01-142).

We also distributed the survey to professional mailing lists consisting of Japanese intensive care nurses through a nonprofit organization called the Japanese Society of Education for Physicians and Trainees in Intensive Care (JSEPTIC). All surveys were conducted with Japanese domestic nurses.

### Demographic data

We extracted characteristics, including age, sex, marital status, number of children, hobbies, exercise habits, annual income, academic degree, years of nursing experience and years of ICU experience.

### Measurement of personality traits

When choosing tools to assess personality traits, we relied on both the Big Five and Type-D personality theories. The Big Five theory is widely used in psychology and is one of the most established approaches to describe individual personality traits [26]. On the other hand, the Type D personality theory was used to initially evaluate the association between patients with heart disease and mortality [27]; however, a recent study revealed an impact of Type D personality on other chronic diseases [28, 29]. As BOS and other occupational stress is often chronic, we thus assumed that Type D personality would also affect nurse BOS [30] and post-traumatic stress disorder symptoms (PTSD) [31]. The already-validated Japanese version of the ten-item personality measure (TIPI) is a measurement tool based on the Big Five theory consisting of 10 items and a 7-point Likert-scale to assess extraversion, agreeableness, conscientiousness, emotional stability and openness to experience [32]. We applied the original cutoff point for four subjective degrees ("Low", "Medium Low", "Medium High", "High") for each personality trait [33] but treated them as dichotomous (low and medium low as low, medium high and high as high) since skewed distributions and outliers might impart bias to the association between personality, ethical climate and BOS symptoms. Treating these personality traits as dichotomous increases the robustness of the interpretation. We also used the already-validated Japanese version of the Type-D personality Scale-14 (DS14) to test for Type D personalities [34]. The DS14 consists of 14 items on a 5-point Likert scale to assess Negative Affectivity (NA) and Social Inhibition (SI). Scores equal to or above 10 on both NA and SI indicate a Type D personality [34].

### Measurement of ethical climate and professional QOL

We measured HEC by the already-validated Japanese version of the Hospital Ethical Climate Survey (HECS) which consists of 26 items to assess the ethical climate of a workplace [35, 36]. HECS is the one of the most widely used surveys, having been translated into many languages [37–39], and it is recognized as having good psychometric properties for measuring ethical climates in health care organizations such as hospitals [35]. There are five domains, including peers, patients, managers, hospital and physicians, and a 5-point Likert scale that measures agreement with statements for each domain. We measured professional QOL by the already-validated Japanese version of the Professional Quality of Life Scale Version 5 (Pro-QOL V) [40]. The Pro-QOL V is the commonly used to measure BOS, STS, and CS in nurses, and consists of 30 items (10 items for each symptom) and a 5-point Likert scale that measures agreement with statements for each item [40]. Each subscale score of 22 or less were considered “low”, levels of 23–41 were considered “moderate” and levels of 42 and above suggested “high” levels of the subject symptom.

### Statistics

#### Multivariate modeling

For dichotomous variables we used Fisher’s exact test for two groups, along with t-testing and ANOVA, followed by Tukey’s test for multiple comparisons of continuous variables in univariate analyses. The outcome of interest was the relationship between personality traits and professional QOL. We evaluated each personality trait based on the Big Five theory and the association between BOS and the BOS-related symptoms STS and CS. We also evaluated the association between Type D personality and BOS, STS and CS. We used age [9, 41, 42], sex [8, 9], marital status [9, 43], children [8], exercise habit [44] and experience years in ICU [41, 45] as co-variates chosen *a priori* from the current literature. We applied general additive models (GAM) [46, 47] to examine the relationship between objective variables (BOS, STS and CS) and explanatory variables. GAM uses a non-linear link function to estimate relationships between objective variables and a smoothed function of explanatory variables [48]. A p<0.05 was considered statistically significant for all parameters.

### Interactions

We hypothesized that HEC mediates the relationship between personality traits and each aspect of professional QOL (BOS, STS and CS). Therefore, we assumed the severity of the relationship between personality traits and professional QOL aspects is not equal by stratifying the severity of the HEC. Thus, we conducted GAM modeling, including only the main effects as above (Model 1), but we also conducted interaction modeling using a thin-plate smoothing spline [49] in the broad sense of smoothing splines for ANOVA (SSANOVA)(Model 2), Interaction modeling can explain the influence between the HEC level and personality traits on the outcome. All data were analyzed using the R software package.

### Sample size calculation

Sample sizes were calculated based on the relationship between BOS and personality traits. Based on previous research, we assumed that effect size is slightly small (f^2^ = 0.02) and we determined that 264 observations would be required for a significance level (α) of 0.05 and test power (1-β) of 0.80.

### Ethics

This study was carried out under laws equivalent to or derived from the principles of the Declaration of Helsinki and was approved by the University of Tsukuba Institutional Review Board (approval # R01-142).

### Summary of study method

**Fig 1:**
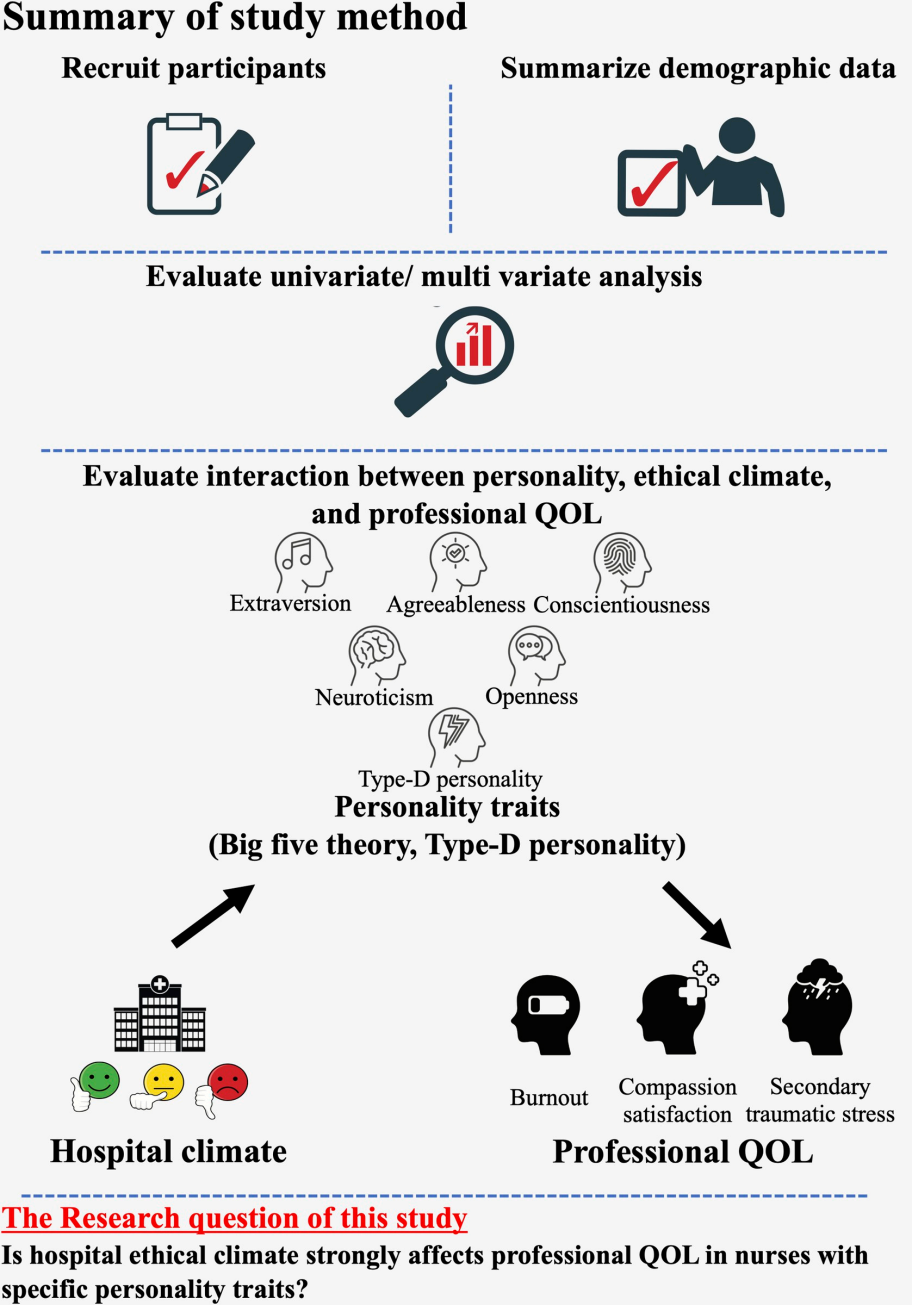
Summary of the study method. This figure shows the summary of the study method.

A summary of the study method is shown in Fig 1.

## Results

### Demographic data

We enrolled 310 participants from September 2019 to February 2020 and demographic data is shown in Table 1. The mean age of the participants was 33.1 years (± 5.9) and about 70% were female. Approximately 55% of the participants were married and 65% were childless. About half (54%) of the participants had hobbies and 29% had an exercise habit. Approximately 40% of the nurses had diplomas, with 35% of the nurses having a bachelor’s degree and 1% had a doctorate. A majority of the nurses (36%) earned between 47,000–57,000 USD annually and had an average nursing experience duration of 11.1 (± 5.5) years and average ICU experience of 6.3 (± 3.3) years.

**Table 1:** Baseline characteristics of participants.

variable	Adult intensive care unit N = 160	Adult/pediatric intensive care unit N = 74	Pediatric intensive care unit N = 76	Total population N = 310
Age ± SD	33.1 ± 5.7^†^	34.3 ± 6.3*	33.1 ± 5.9	33.1 ± 5.9
Female n (%)	46 (29)	28 (38)	24 (32)	212 (68)
Marital status, Married (%)	90 (50)	41 (55)	44 (57)	173 (55)
Children, n (%)				
No children	101 (63)	45 (61)	56 (73)	202 (65)
One child	38 (24)	20 (27)	15 (20)	73 (24)
Two children	17 (11)	8 (11)	4 (5)	29 (9)
Three or more children	4 (3)	1 (1)	1 (1)	6 (2)
Hobby, n (%)	85 (53)	47 (63)	36 (47)	168 (54)
Exercise, n (%)	39 (24)	23 (31)	28 (36)	90 (29)
Annual income, n (%)				
< USD 27,000	4 (2)	3 (4)	3 (4)	10 (3)
USD 27,000–37,000	44 (28)	20 (27)	9 (12)	73 (24)
USD 37,000–47,000	45 (28)	18 (24)	26 (34)	89 (29)
USD 47,000–57,000	52 (32)	28 (38)	32 (42)	112 (36)
USD 57,000–65,000	9 (6)	2 (3)	5 (6)	16 (5)
>USD 65,000	6 (4)	3 (4)	1 (1)	10 (3)
Education level, n (%)				
Diploma	67 (42)	23 (31)	38 (50)	128 (41)
Junior college	26 (16)	10 (13.5)	9 (12)	45 (15)
Bachelor’s degree	54 (34)	28 (38)	26 (34)	108 (35)
Master’s degree	11 (7)	12 (16)	3 (4)	26 (8)
Doctor’s degree	2 (1)	1 (1)	0 (1)	3 (1)
Years of nursing experience, Average ± SD	11.2 ± 5.4	12.1 ± 5.8	10.1 ± 5.4	11.1 ± 5.5
Years of ICU experience, years, Average ± SD	6.5 ± 3.3	6.0 ± 3.4	6.3 ± 3.1	6.3 ± 3.3

*: significantly different from Adult intensive care unit, †:: significantly different from Adult/pediatric intensive care unit.

‡: significantly different from pediatric intensive care unit.

### Distribution of personality traits

**Fig 2:**
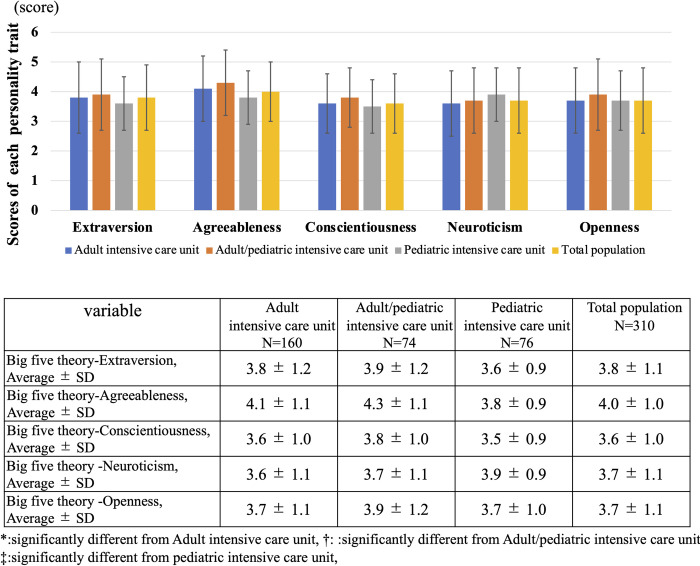
Distribution of personality traits based on big five theory. This figure shows the distribution of personality traits for based on Big Five theory for each intensive care unit.

As shown in Fig 2, the distribution of personality traits based on the Big Five theory did not significantly differ between Adult ICU, Adult/pediatric ICU and PICU categories for extraversion (Adult ICU: 3.8 ± 1.2 vs. Adult/pediatric ICU: 3.9 ± 1.2 vs. PICU: 3.6 ± 0.9, p = 0.35), agreeableness (Adult ICU: 4.1 ± 1.1 vs. Adult/pediatric ICU: 4.3 ± 1.1 vs. PICU: 3.8 ± 0.9, p = 0.05), conscientiousness (Adult ICU: 3.6 ± 1.0 vs. Adult/pediatric ICU: 3.8 ± 1.0 vs. PICU: 3.5 ± 0.9, p = 0.28), neuroticism (Adult ICU: 3.6 ± 1.1 vs. Adult/pediatric ICU: 3.7 ± 1.1 vs. PICU: 3.9 ± 0.9, p = 0.21) and openness (Adult ICU: 3.7 ± 1.1 vs. Adult/pediatric ICU: 3.9 ± 1.2 vs. PICU: 3.7 ± 1.0, p = 0.34).

**Fig 3:**
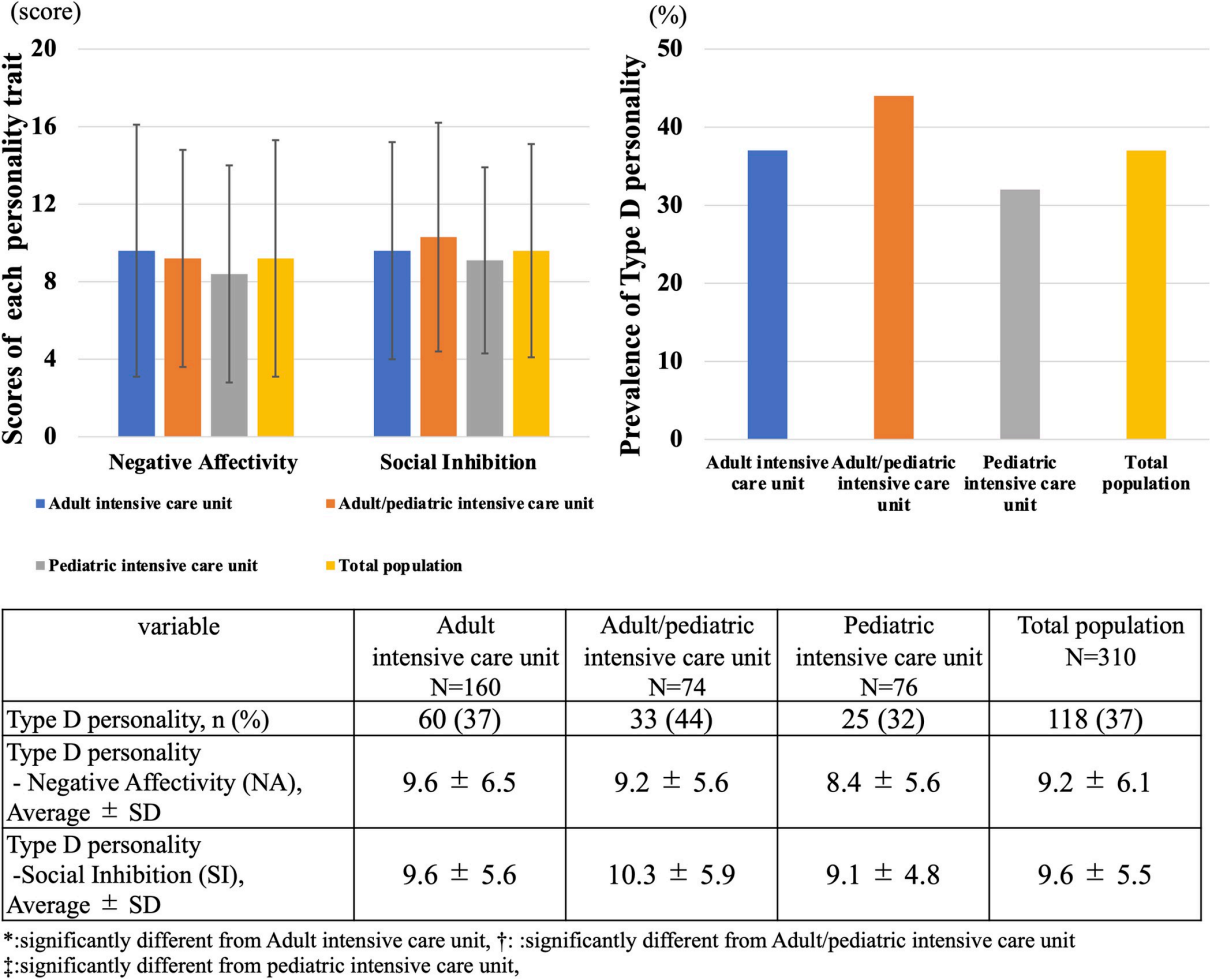
Distribution of personality traits based on type D personality. This figure shows the distribution of personality traits for based on Type D personality for each intensive care unit.

With regard to the distribution of personality traits based on Type-D personality shown in Fig 3, the prevalence of Type-D personality did not significantly differ between Adult ICU, Adult/pediatric ICU and PICU categories (Adult ICU: 37% vs. Adult/pediatric ICU: 44% vs. pediatric ICU: 32%, p = 0.67). Differences in the components of Type-D personality, NA (Adult ICU: 9.6 ± 6.5 vs. Adult/pediatric ICU: 9.2 ± 5.6 vs. pediatric ICU: 8.4 ± 5.6, p = 0.37) and SI (Adult ICU: 9.6 ± 5.6 vs. Adult/pediatric ICU: 10.3 ± 5.9 vs. pediatric ICU: 9.1 ± 4.8, p = 0.37) categories were also not significantly different.

### Severity of hospital ethical climate

**Fig 4:**
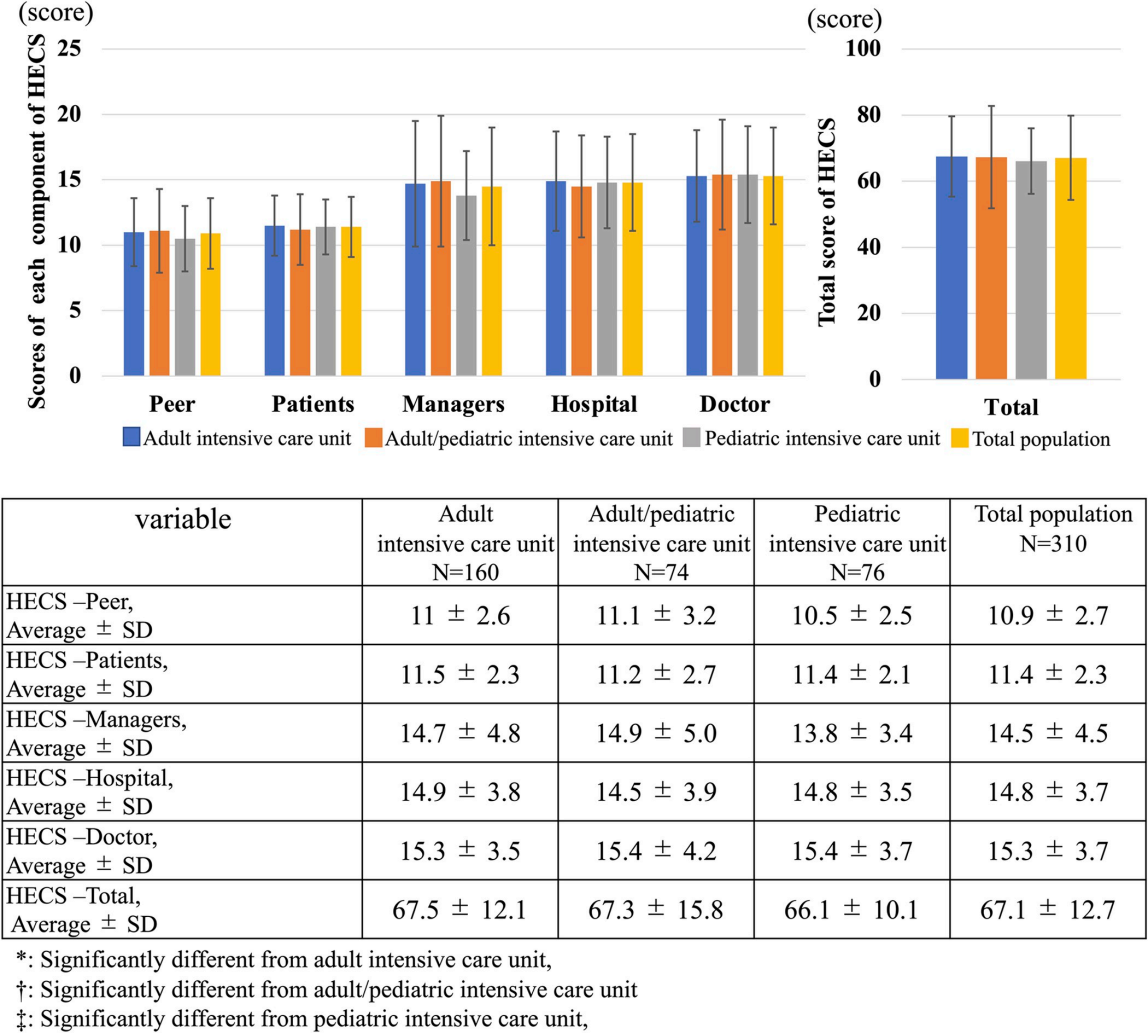
Severity of hospital ethical climate. This figure shows the distribution of Severity of hospital ethical climate for each intensive care unit.

As shown in Fig 4, The severity of total of ethical climate did not significantly differ between categories (Adult ICU: 67.5 ± 12.1 vs. Adult/pediatric ICU: 67.3 ± 15.8 vs. pediatric ICU: 66.1 ± 10.1, p = 0.69) and no significant differences were seen for each component: peers (Adult ICU: 11 ± 2.6 vs. Adult/pediatric ICU: 11.1 ± 3.2 vs. PICU: 10.5 ± 2.5, p = 0.38), patients (Adult ICU: 11.5 ± 2.3 vs. Adult/pediatric ICU: 11.2 ± 2.7 vs. PICU: 11.4 ± 2.1, p = 0.66), managers (Adult ICU: 14.7 ± 4.8 vs. Adult/pediatric ICU: 14.9 ± 5.0 vs. PICU: 13.8 ± 3.4, p = 0.27), hospital (Adult ICU: 14.9 ± 3.8 vs. Adult/pediatric ICU: 14.5 ± 3.9 vs. PICU: 14.8 ± 3.5, p = 0.79) and doctor (Adult ICU: 15.3 ± 3.5 vs. Adult/pediatric ICU: 15.4 ± 4.2 vs. PICU: 15.4 ± 3.7, p = 0.97).

### Average severity of BOS, STS and CS

**Fig 5:**
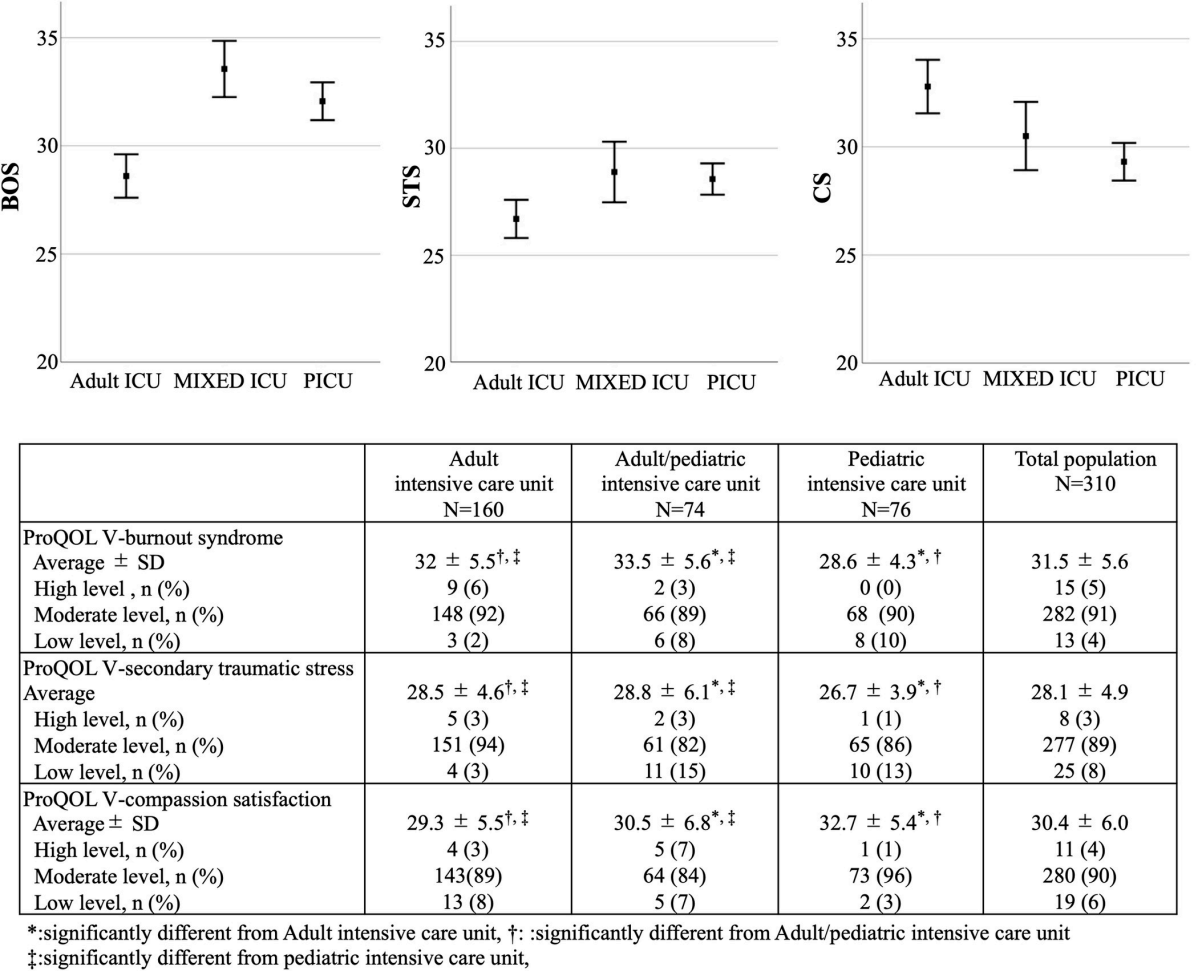
Severity of BOS, STS and CS. This figure shows the distribution of Severity of BOS, STS and CS for each intensive care unit.

As shown in Fig 5, the average BOS score in the PICU category was significantly lower compared with Adult ICU (p<0.05) and Adult/pediatric ICU categories (p<0.05) (Adult ICU: 32 ± 5.5 vs. Adult/pediatric ICU: 33.5 ± 5.6 vs. PICU: 28.6 ± 4.3) while the average of severity of STS in the PICU category was also significantly lower compared with Adult ICU (p<0.05) and Adult/pediatric ICU categories (p<0.05) (Adult ICU: 28.5 ± 4.6 vs. Adult/pediatric ICU: 28.8 ± 6.1 vs. PICU: 26.7 ± 3.9). Moreover, the average severity of CS in the PICU category was significantly higher compared with Adult ICU (p<0.05) and Adult/pediatric ICU categories (p<0.05) (Adult ICU: 29.3 ± 5.5 vs. Adult/pediatric ICU: 30.5 ± 6.8 vs. PICU: 32.7 ± 5.4).

### Factors associated with BOS, STS and CS by univariate analysis

We show the results of our univariate analysis in S1 Data (available online).

### Associations between personality traits and HEC for BOS, STS and CS by multivariate analysis

Table 2 shows the results of the multivariate analysis. Neuroticism (p = 0.03), Type-D personality (p<0.01) plus each component of type D personality, NA (p<0.01) and SI (p = 0.01) and total HECS score (p<0.01) plus each component of HECS (except Peers) were associated with increased BOS (Patients: p<0.01, Managers: p<0.01, Hospital: p<0.01, Doctor: p<0.01). All the risk factors for increased BOS were also risk factors for increased STS and agreeableness (p<0.01), and peer component of HECS (p<0.01) were an additional associated factor for STS. For CS, extraversion (p = 0.01), agreeableness (p<0.01), openness (p = 0.01) and total HECS score (p<0.01) plus each component of HECS were associated with increased CS (p<0.01 for all). Of all the multivariate modeling, only exercise habit was independently associated with increased BOS and STS but decreased CS. Detailed results of multivariate modeling, including covariates for each personality trait and HEC (including covariates), are available online. (S2 Data).

**Table 2:** Multivariate model for BOS, CF, STS.

Variable	Burnout Odds ratio (95%CI)		p-value	Secondary traumatic stress Odds ratio (95%CI)		p-value	Compassion Satisfaction Odds ratio (95%CI)		p-value
Big Five theory-Extraversion	0.6	(0.14–2.48)	0.48	0.31	(0.06–1.63)	0.17	12.3	(1.78–85.7)	0.01
Big Five theory-Agreeableness	1.53	(0.31–7.55)	0.47	0.12	(0.03–0.51)	<0.01	18.2	(3.42–97.1)	<0.01
Big Five theory-Conscientiousness	2.92	(0.26–32.7)	0.38	4.52	(0.51–39.7)	0.17	1.1	(0.08–14.7)	0.93
Big Five theory -Neuroticism	5.06	(1.1–23.2)	0.03	5.2	(1.32–20.5)	0.01	0.21	(0.04–1.09)	0.06
Big Five theory -Openness	1.94	(0.31–12)	0.47	0.31	(0.06–1.63)	0.17	12.3	(1.78–85.7)	0.01
Type D personality	13.2	(3.91–45)	<0.01	19.7	(6.69–58.2)	<0.01	0.37	(0.09–1.42)	0.15
Type D personality	GAM modeling: positive association		<0.01	GAM modeling: positive association		<0.01	GAM modeling: negative association		0.33
- Negative Affectivity (NA)									
Type D personality	GAM modeling: positive association		0.01	GAM modeling: positive association		<0.01	GAM modeling: negative association		0.66
-Social Inhibition (SI)									
HECS-Total	GAM modeling: negative association		<0.01	GAM modeling: negative association		<0.01	GAM modeling: positive association		<0.01
HECS–Peers	GAM modeling: negative association		0.07	GAM modeling: negative association		<0.01	GAM modeling: positive association		<0.01
HECS–Patients	GAM modeling: negative association		<0.01	GAM modeling: negative association		0.01	GAM modeling: positive association		<0.01
HECS–Managers	GAM modeling: negative association		<0.01	GAM modeling: negative association		<0.01	GAM modeling: positive association		<0.01
HECS–Hospital	GAM modeling: negative association		<0.01	GAM modeling: negative association		<0.01	GAM modeling: positive association		<0.01
HECS–Doctors	GAM modeling: negative association		<0.01	GAM modeling: negative association		<0.01	GAM modeling: positive association		<0.01

We used age, sex, marital status, children, exercise habit and years of ICU experience as co-variate.

Based on GAM modeling, we express associations as positive, negative or fluctuated. Representations of data shapes are provided in S2 Data.

### Interaction between HEC and personality traits based on Big Five theory for BOS, STS and CS

**Fig 6:**
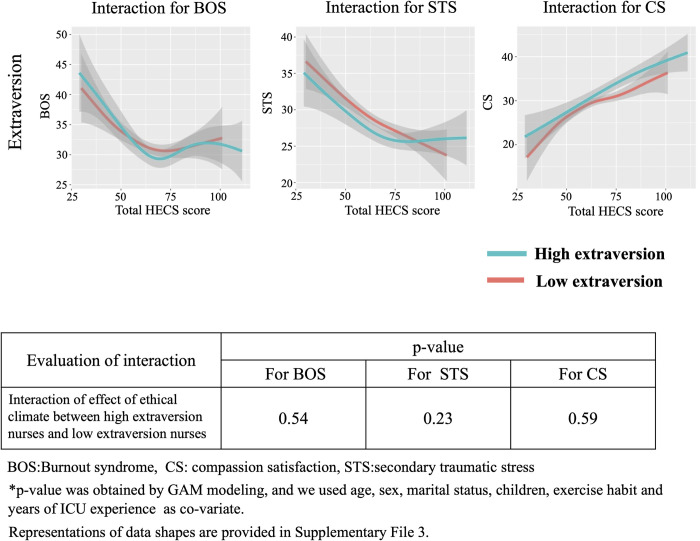
Interactions between extraversion, BOS, STS and CS. This figure shows the interaction between ethical climate and high and low extraversion based on Big Five theory. GAM modeling shows there is no interaction between ethical climate and extraversion for BOS, STS and CS.

**Fig 7:**
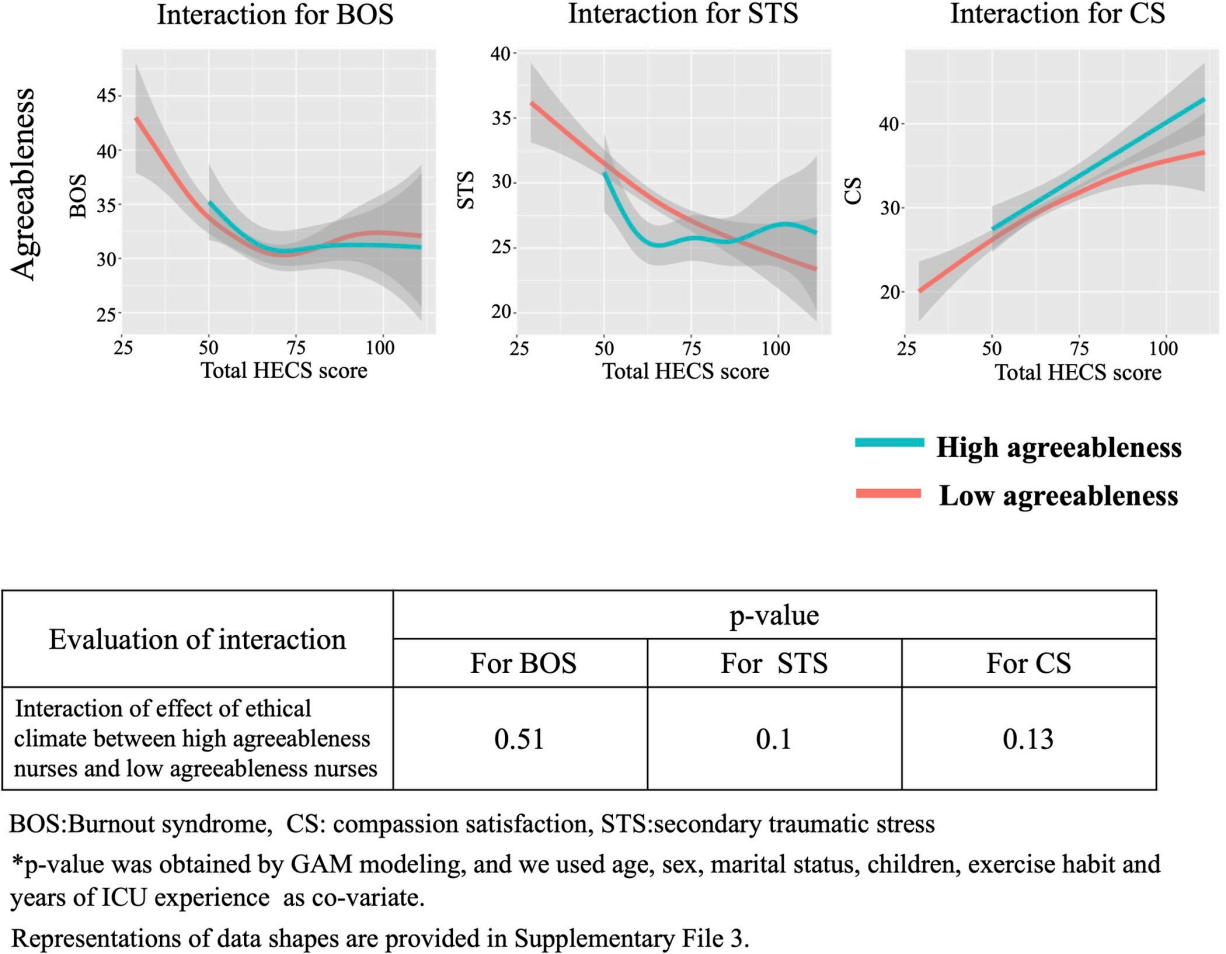
Interactions between agreeableness, BOS, STS and CS. This figure shows the interaction between ethical climate and high and low agreeableness based on Big Five theory. GAM modeling shows there is no interaction between ethical climate and agreeableness for BOS, STS and CS.

**Fig 8:**
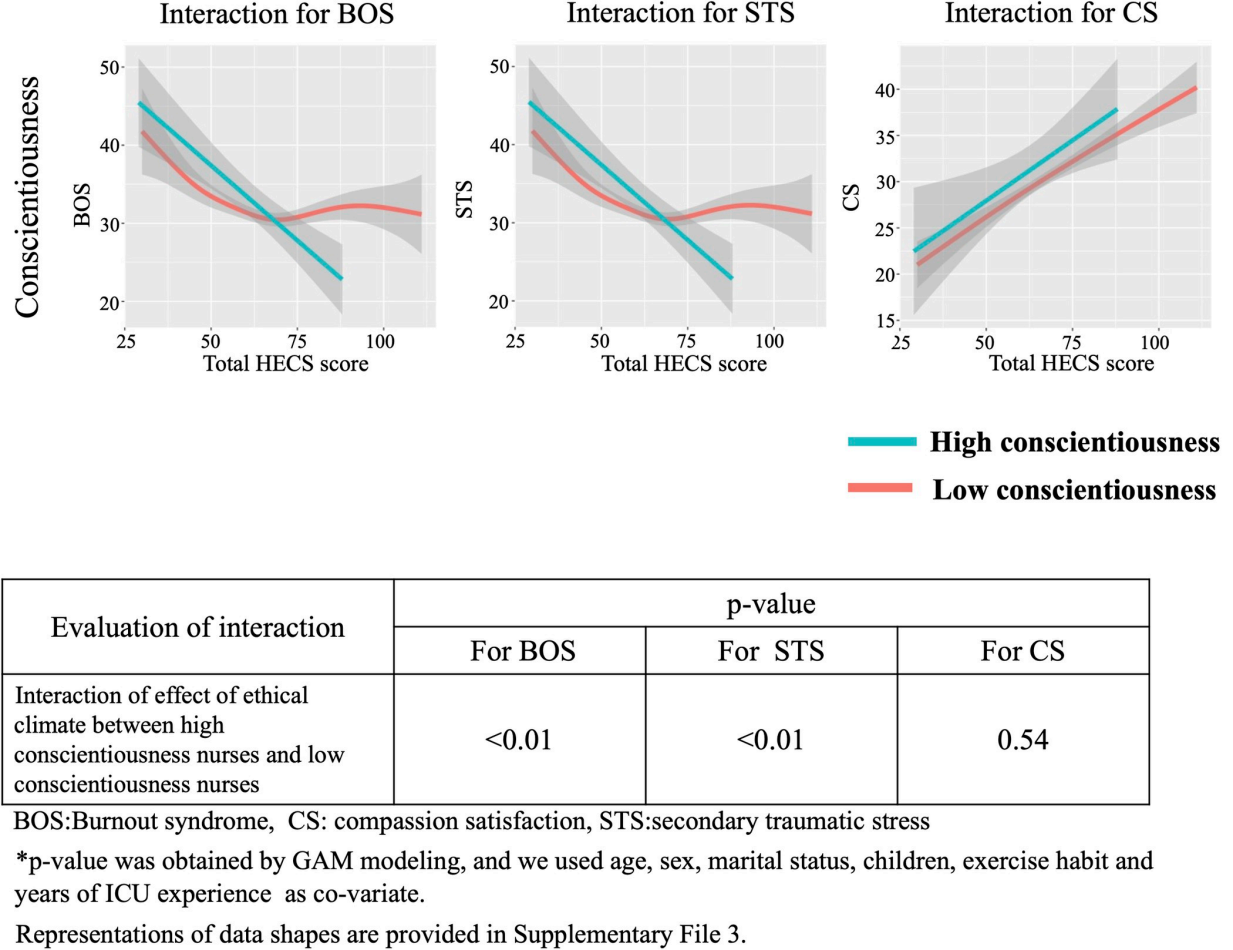
Interactions between conscientiousness, BOS, STS and CS. This figure shows the interaction between ethical climate and high and low conscientiousness based on Big Five theory. GAM modeling shows there is an interaction between ethical climate and conscientiousness for BOS and STS, but not CS.

**Fig 9:**
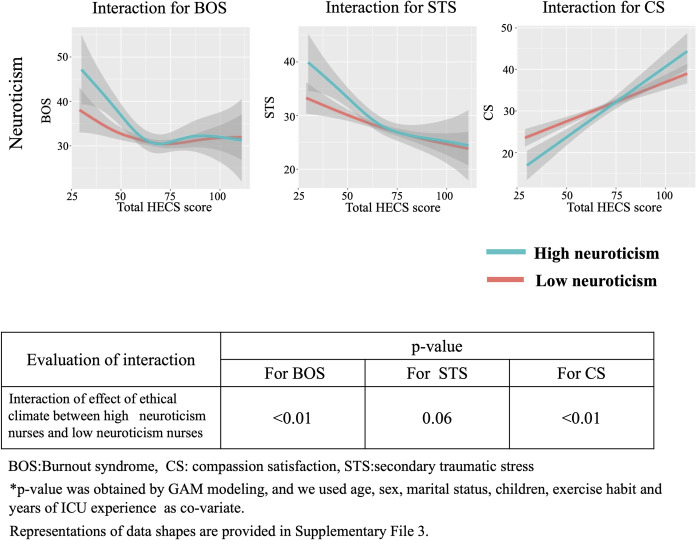
Interactions between neuroticism, BOS, STS and CS. This figure shows the interaction between ethical climate and high and low neuroticism based on Big Five theory. GAM modeling shows there is an interaction between ethical climate and conscientiousness for BOS and CS, but not STS.

**Fig 10:**
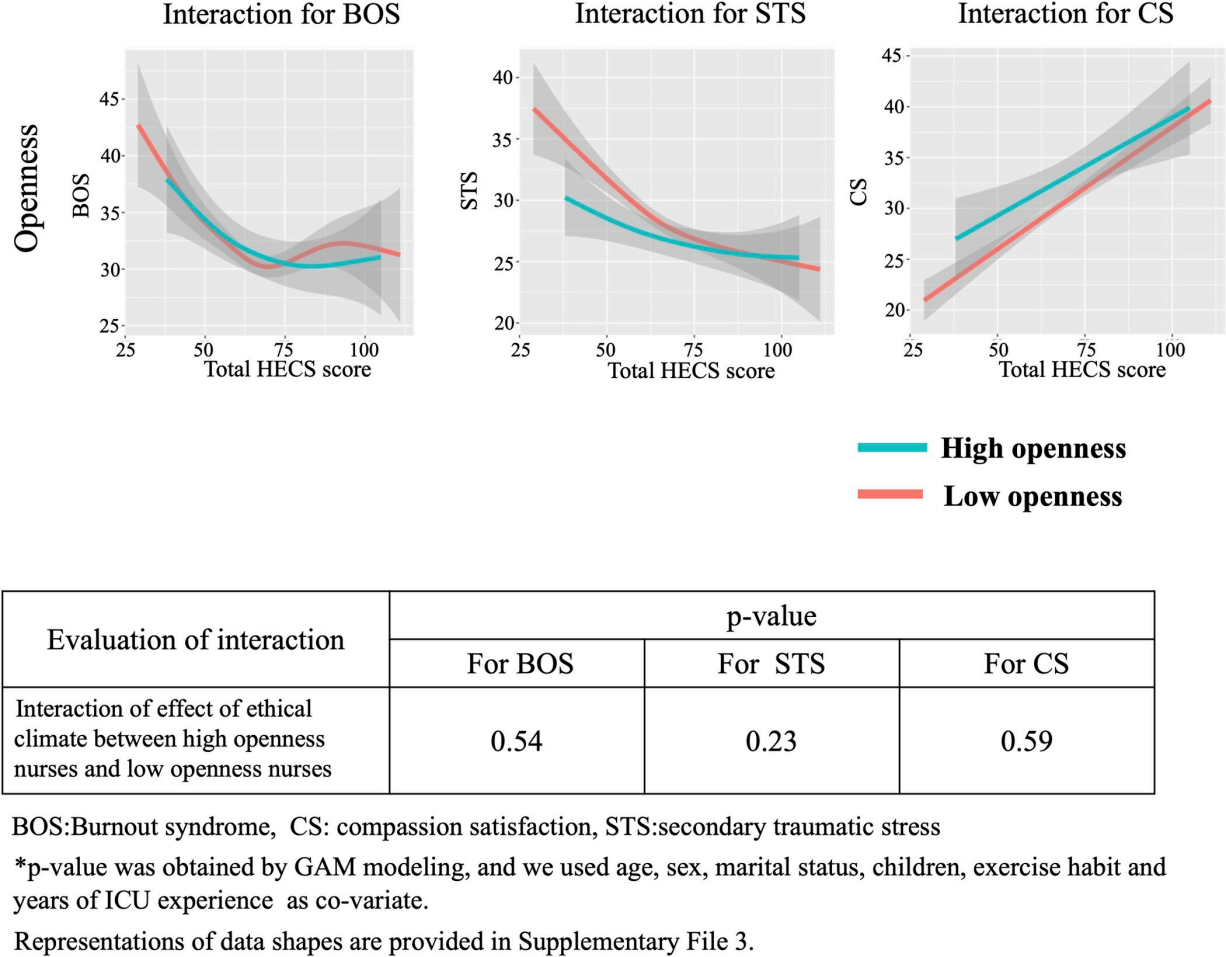
Interactions between openness, BOS, STS and CS. This figure shows the interaction between ethical climate and high and low openness based on Big Five theory. GAM modeling shows there is no interaction between ethical climate and openness for BOS, STS and CS.

We show the interaction between ethical climate and personality traits based on the Big Five theory for BOS, STS and CS in Figs 6–10. In Fig 6, GAM modeling shows no interactions between ethical climate and extraversion for BOS (p = 0.54), STS (p = 0.23), or CS (p = 0.59). This result indicates that ethical climate affects professional QOL equally between high and low extraversion nurses. In Fig 7, GAM modeling shows no interactions between ethical climate and agreeableness for BOS (p = 0.51), STS (p = 0.1), or CS (p = 0.13), indicating that ethical climate affects professional QOL equally between high and low agreeableness nurses. In Fig 8, GAM modeling shows an interaction between ethical climate and conscientiousness for BOS (p<0.01) and STS (p<0.01) but not CS (p = 0.54). This indicates that ethical climate more seriously affects high conscientiousness nurses for BOS and STS. Fig 9 shows the interaction between ethical climate and neuroticism. GAM modeling shows an interaction between ethical climate and neuroticism for BOS (p<0.01) and CS (p<0.01), but not STS (p = 0.06), indicating that ethical climate more seriously affects high neuroticism nurses for BOS and CS. In Fig 10, GAM modeling shows no interactions between ethical climate and openness for BOS (p = 0.54), STS (p = 0.23), or CS (p = 0.59), indicating that ethical climate affects professional QOL equally between high and low openness nurses. We also observed that high conscientiousness nurses did not answer more than 88 out of 120 questions for HECS compared with 111 out of 120 questions in the low conscientiousness group. This difference was also seen in agreeableness as high agreeableness nurses did not answer less than 50 out of 120 questions for HECS compared with 22 out of 120 questions in the low agreeableness group.

### Interaction between ethical climate and Type D personality for BOS, STS and CS

**Fig 11:**
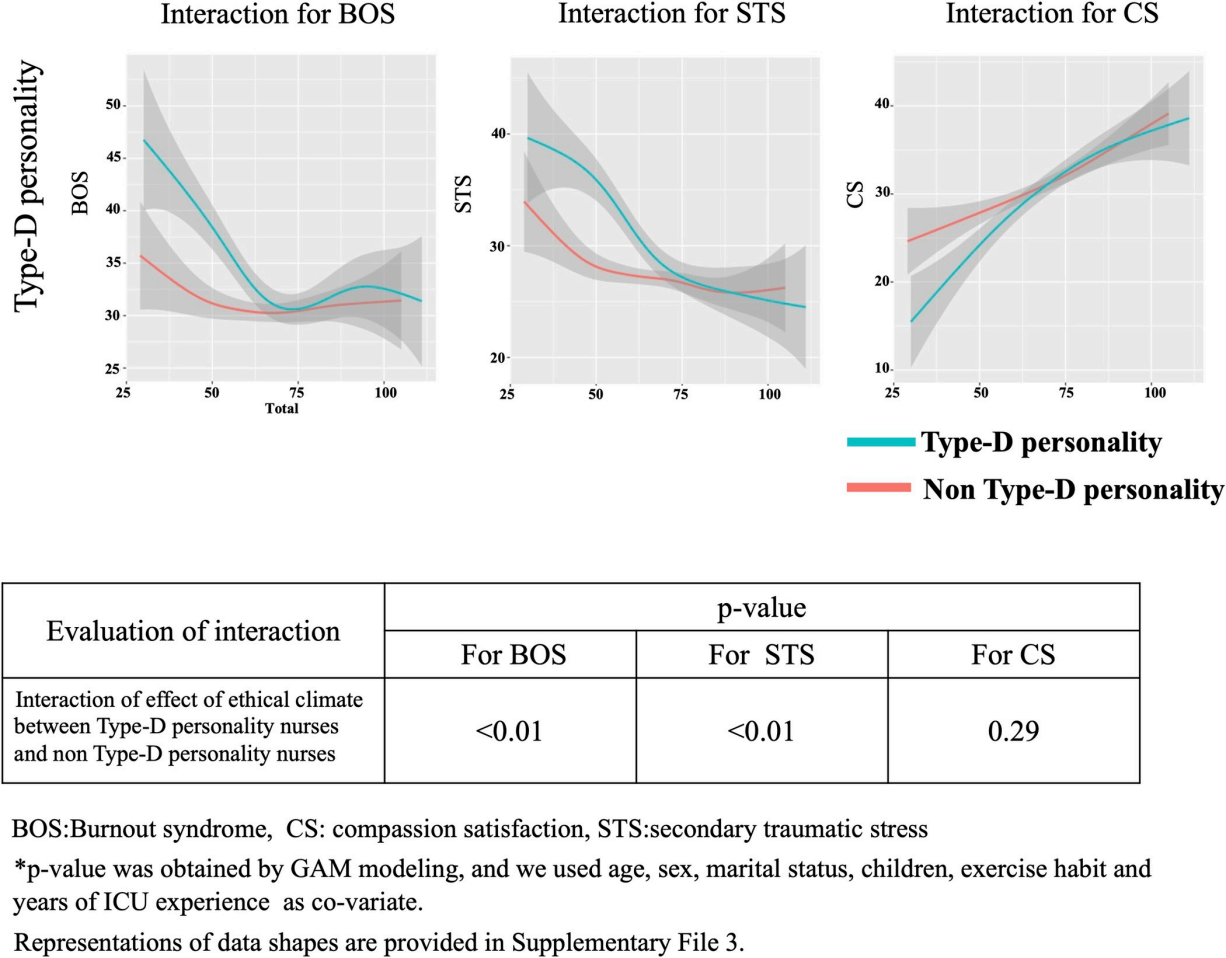
Interactions between Type-D personality traits and BOS, STS and CS. This figure shows the interaction between absence and presence of Type-D personality traits. GAM modeling shows there is an interaction between ethical climate and conscientiousness for BOS and STS, but not CS.

We also show the interaction between HEC and Type D personality for BOS, STS and CS in Fig 11. GAM modeling shows there is an interaction between ethical climate and Type-D personality for BOS (p<0.01) and STS (p<0.01), but not CS (p = 0.29), indicating that ethical climate more seriously affects Type-D personality nurses for BOS and STS.

### Summary of study results

**Fig 12:**
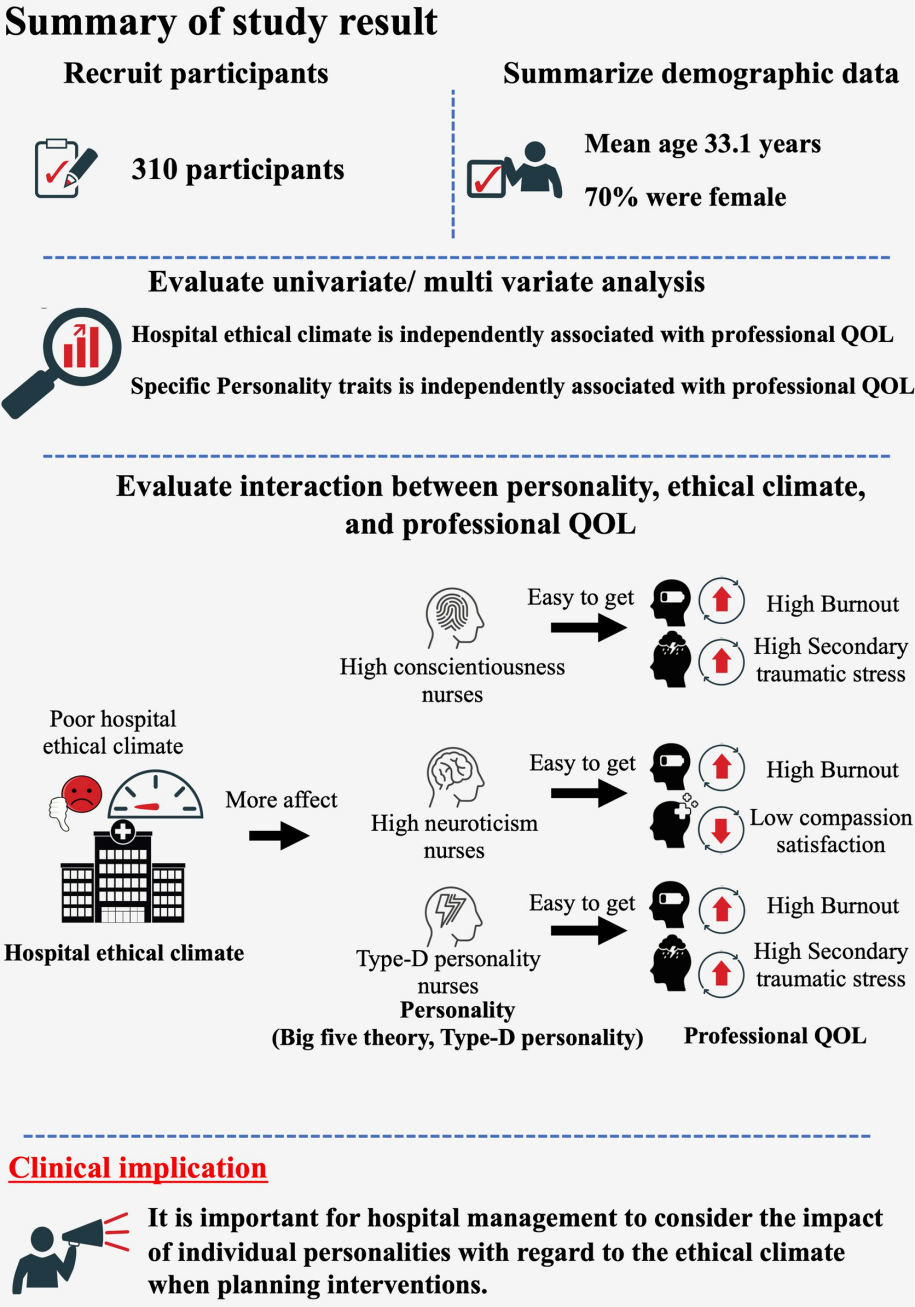
Summary of the study results. This figure shows the summary of the study results.

Study results are summarized in Fig 12.

## Discussion

The present study is the first to reveal the relationship between personality traits, hospital ethical climate and professional QOL. Here, we showed that HEC is independently associated with professional QOL (BOS, STS, CS) (Hypothesis 1) and also that high conscientiousness and high neuroticism drive BOS and STS under conditions of a poor hospital ethical climate. Moreover, Type D personality is also a key factor in BOS and STS under a poor hospital ethical climate, indicating that HEC strongly affects the magnitude of professional QOL in nurses with specific personality traits. (Hypothesis 3).

The average BOS, STS and CS scores in our cohort were 31.5 (± 5.6), 28.1 (± 4.9) and 30.4 (± 6.0), respectively, and these average BOS and STS scores are similar to a previous report evaluating all nurses in Japan [40]. The STS average scores we obtained was also similar to a previous study from China regarding various departments, including the ICU [25], which indicates that our cohort was an appropriate cross section of nurses and that our results are generally applicable to Asian nursing populations.

We also revealed differences with regard to professional QOL between adult, adult/pediatric and PICU nurses. The PICU nurses had significantly lower BOS and STS, coupled with significantly high CS, even though the demographic data and personality traits were not significantly different. As a previous study reported that those in pediatric care are subject to high levels of stress and burnout risk [7] and a meta-analysis also revealed a BOS high prevalence in pediatric nurses, this conflicts with our results [50]. However, as most studies did not evaluate both adults and children, possibly imparting selection bias, our comparative study adds to the scarce body of knowledge on this topic.

Another systematic review indicated that adult intensive care nurses are a high risk population [51] but, as the PICU is still a new therapeutic area [52], there are fewer professional QOL comparisons between PICU and other nursing professions. One study that included both adult and pediatric intensive care nurses found no differences [53] but adult intensive care unit nurses may more frequently face the deaths of their patients, impacting professional QOL [54]. Taking into account that observing death is a strong risk factor for BOS [12], our finding that pediatric nurses have lower BOS and STS but higher CS is reasonable.

Regarding the risk factors for BOS and CS, exercise habits are significantly associated with BOS and CS. As a previous review already reported reductions in BOS within general occupational health [44], our study is in line with this review and also showed the robustness of the strong association between exercise and BOS. Interestingly, exercise was significantly associated not only with BOS but CS. As Stamm et al conceptualized BOS, CS and STS as the facets of professional QOL, it is sensible that exercise’s effect on reduction of BOS could increase CS [23].

We also revealed an interaction between hospital ethical climate and specific personality traits for BOS, CS and STS. According to the Big Five theory, high conscientiousness, a Type D personality and neuroticism are key factors for BOS and STS within a poor hospital ethical climate, creating the idea that personality is a key factor in assessing professional QOL. This makes accurate pre-employment quantification of these traits crucial as a predictor of BOS/STS development.

Several studies have revealed the associations between personality and BOS and ethical climate and BOS. However, humans are dual aspect creatures living in both a social community and as individuals. Thus, the ethical climate must be thought of as a representative barometer of the atmosphere of the working place as a social community while personality traits are representative of the individual thinking. From our result, hospital ethical climate and personality traits with regard to professional QOL and its facets, including BOS, STS and CS, are thus significantly associated. Therefore, for clinical implications, it is important for hospital management to consider the impact of the ethical climate with regard to nursing staff safety and preservation when planning interventions. For BOS intervention, there are already reports that some psychological interventions are helpful in nurses. For example, a previous randomized controlled trial (RCT) reported that recording three good things each day significantly improved self-efficacy and job performance in BOS nurses [55] while an RCT called STOP THE BURN is ongoing to reveal the effect of “death cafés” for preventing BOS in ICU nurses [56]. The still-new death café concept is a debriefing method focusing on death now being studied for its ability to bring self-efficacy to applicable fields [57]. As BOS is a psychological change in response to chronic job stressors [58], self-efficacy is an important aspect for maintaining mental health [59] and improving it by recording positive aspects of the daily life to share with co-workers might reduce BOS in these susceptible occupations.

This observational study demonstrated links between personality traits, ethical climate and professional QOL. Further studies should be conducted on interventional approaches that consider personality traits in counseling to increase professional QOL. Because a substandard ethical climate greatly decreases professional QOL for specific personalities, this association must be considered for interpretation of any intervention-based results.

There is still a worldwide shortage of nurses, estimated to reach to 7.6 million in the year 2030 [60]. This number will invariably be inflated because of the COVID-19 pandemic and medical staff are now facing difficulties that place tremendous pressure on professional QOL. Taking into account the close relationship between personality, compassion, ethical climate and professional QOL, new studies focusing on strategies to retain nurses will be needed to ameliorate the nursing shortage in this pandemic age.

### Limitations

There are some limitations to this study. First, we conducted a cross-sectional study and therefore cannot mention any causal relationships. Second, we used an online survey system for gathering participants which might have led to self-selection and/or non-respondent bias. Self-selection and non-respondent biases are difficult to control for in questionnaire studies since we could not survey the non-responders for ethical reasons and these nurses may have refused participation because of a higher level of BOS. However, in spite of the limitations inherent in a cross-sectional study, this report still demonstrates a significant interaction between hospital ethical climate and specific personality traits with regard to professional QOL that could serve to bring insight into counteracting BOS and STS in PICU nurses.

## Conclusion

We conducted a cross-sectional study showing hospital ethical climate and specific personality traits as independent factors for professional QOL. Additionally, based on The Big Five theory, a poor hospital ethical climate precipitates BOS and STS with regard to high conscientiousness while high neuroticism also drives the BOS and worsens CS. Moreover, a Type-D personality is contributive to BOS and STS under a poor hospital ethical climate.

Therefore, it is important for hospital management to consider the impact of individual personalities with regard to the ethical climate when planning interventions.

## Supporting information

S1 Data (PDF)

S2 Data (PDF)
