# Supplementary material for: PERSonality, Ehical, and PROfessional quality of life in Pediatric/Adult Intensive Nurses study: PERSEPRO PAIN study
Source: PLoS One. 2022 Mar 7;17(3):e0259721. doi: 10.1371/journal.pone.0259721 (PMC8901072; doi:10.1371/journal.pone.0259721)
Supplement: S1 Data — (PDF) [file pone.0259721.s001.pdf]

Online Data Supplement 1: Result of univariate analysis for BOS,STS,CS

Title: PERSonality, Ehical, and PROfessional quality of life in Pediatric/Adult Intensive Nurses study: PERSEPRO PAIN study

Running title: PERSEPRO PAIN study

Yujiro Matsuishi[1†], Bryan J. Mathis[2†], Haruhiko Hoshino[3], Yuki Enomoto [4,5], Nobutake Shimojo[5], Satoru Kawano[5], Hideaki Sakuramoto[6], Yoshiaki Inoue[5]

1 Neuroscience Nursing, St. Luke's International University, Tokyo, Japan

2 International Medical Center, University of Tsukuba Hospital, Tsukuba, Ibaraki, Japan.

3 Adult Health Nursing, Department of Nursing, International University of Health and Welfare, Narita, Japan

4University of Tsukuba Hospital, Department of Pediatrics, Tsukuba, Ibaraki, Japan.

5Department of Emergency and Critical Care Medicine, Faculty of Medicine, University of Tsukuba, Tsukuba, Ibaraki, Japan.

6Adult Health Nursing, College of Nursing, Ibaraki Christian University, Hitachi, Ibaraki, Japan

†These authors contributed equally to this study.

Running title: PERSE PRO PAIN study

Corresponding author's contact information.

Dr. Yoshiaki Inoue

Department of Emergency and Critical Care Medicine, Faculty of Medicine, University of Tsukuba, Tsukuba, Ibaraki, 305-8575, Japan.

Phone: +81-29-853-5633.

FAX: +81-29-853-3092

E-mail: yinoue@md.tsukuba.ac.jp

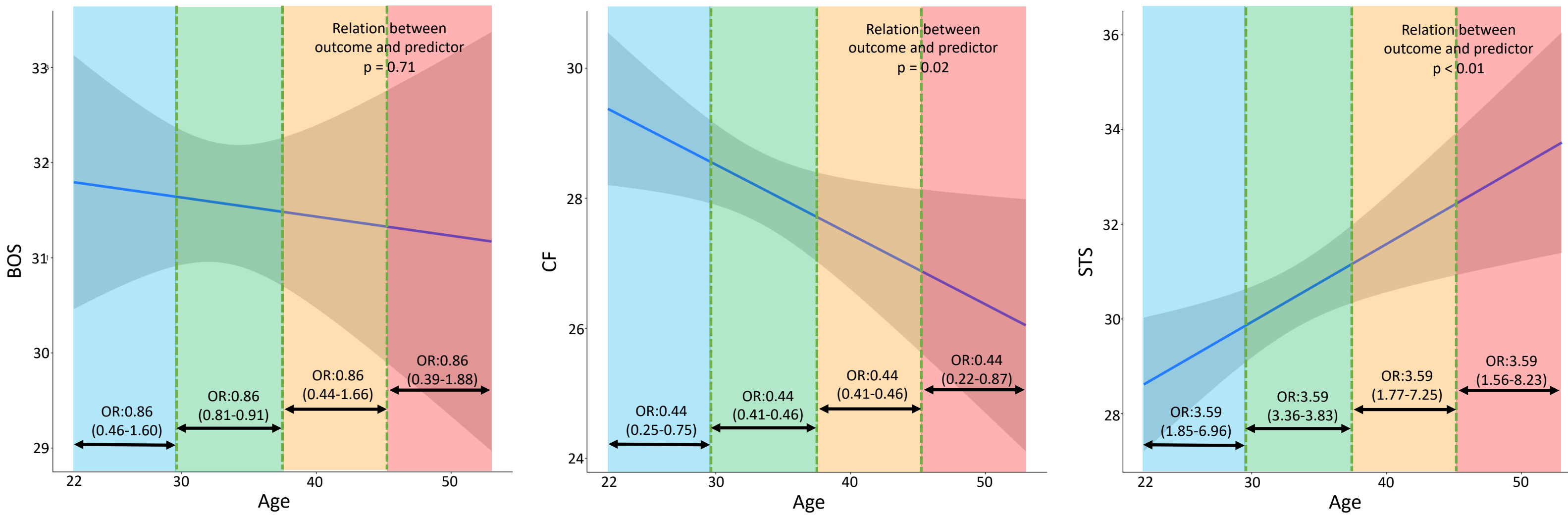

Figure 1: Univariate model of age for BOS, STS and CS

Table 1: Univariate model of age for BOS

Quartile	Range of factor	Odds ratio	95% CI
0th-25th	22-30	0.86	(0.46-1.60)
25th-50th	30-38	0.86	(0.81-0.91)
50th-75th	38-45	0.86	(0.44-1.66)
75th-100th	45-53	0.86	(0.39-1.88)

GAM modeling showed negative association and p-value was p=0.71

Table 2: Univariate model of age for STS

Quartile	Range of factor	Odds ratio	95% CI
0th-25th	22-30	0.44	(0.25-0.75)
25th-50th	30-38	0.44	(0.41-0.46)
50th-75th	38-45	0.44	(0.41-0.46)
75th-100th	45-53	0.44	(0.22-0.87)

GAM modeling showed negative association and p-value was p=0.02

Table 3: Univariate model of age for CS

Quartile	Range of factor	Odds ratio	95% CI
0th-25th	22-30	3.59	(1.85-6.96)
25th-50th	30-38	3.59	(3.36-3.83)
50th-75th	38-45	3.59	(1.77-7.25)
75th-100th	45-53	3.59	(1.56-8.23)

GAM modeling showed positive association and p-value was p<0.01

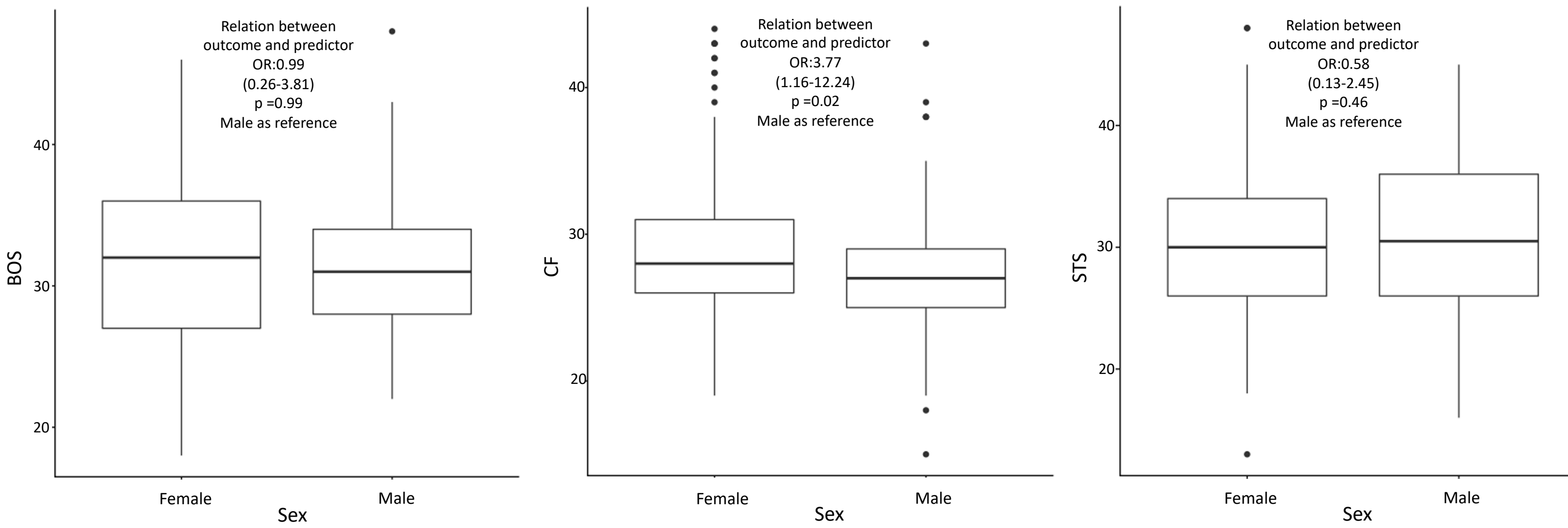

Figure 2: Univariate model of sex for BOS, STS and CS

Table 4: Univariate model of sex for BOS

Reference	Factor	Odds ratio	95%CI	p-value
male	female	0.99	(0.26-3.81)	0.99

Table 5: Univariate model of sex for STS

Reference	Factor	Odds ratio	95%CI	p-value
male	female	3.77	(1.16-12.24)	0.02

Table 6: Univariate model of sex for CS

Reference	Factor	Odds ratio	95%CI	p-value
male	female	0.58	(0.13-2.45)	0.46

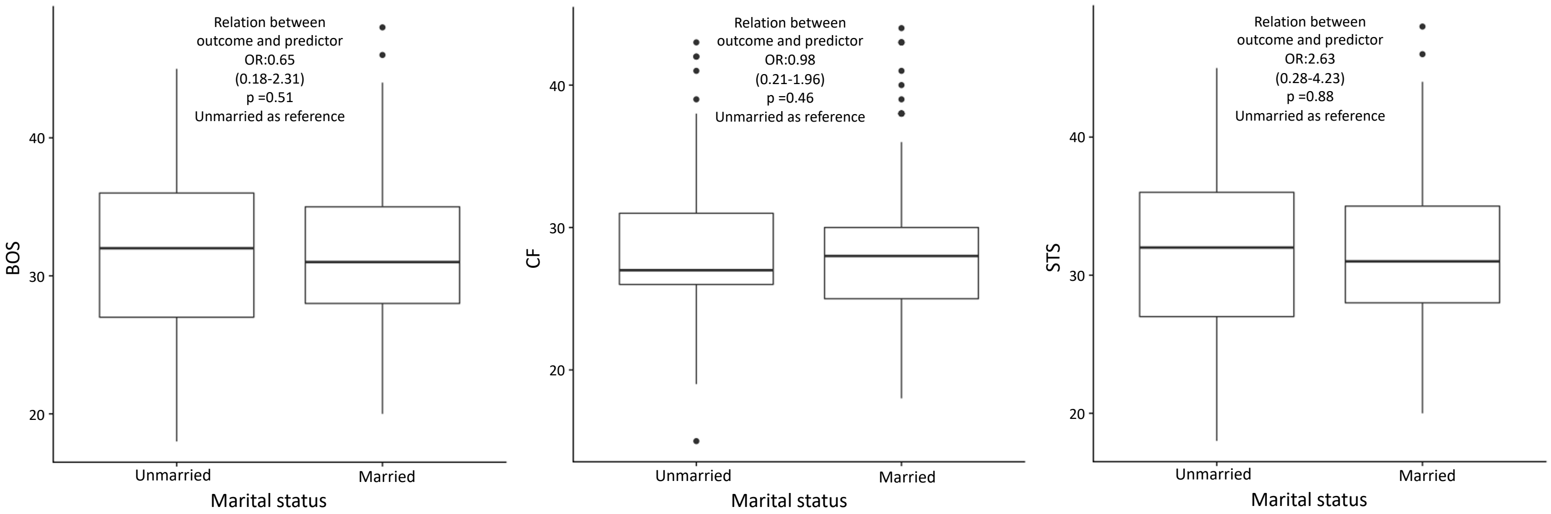

Figure 3: Univariate model of marital status for BOS, STS and CS

Table 7: Univariate model of marital status for BOS

Reference	Factor	Odds ratio	95%CI	p-value
unmarried	married	0.65	(0.18-2.31)	0.51

Table 8: Univariate model of marital status for STS

Reference	Factor	Odds ratio	95%CI	p-value
unmarried	married	0.98	(0.21-1.96)	0.46

Table 9: Univariate model of marital status for CS

Reference	Factor	Odds ratio	95%CI	p-value
unmarried	married	2.63	(0.28-4.23)	0.88

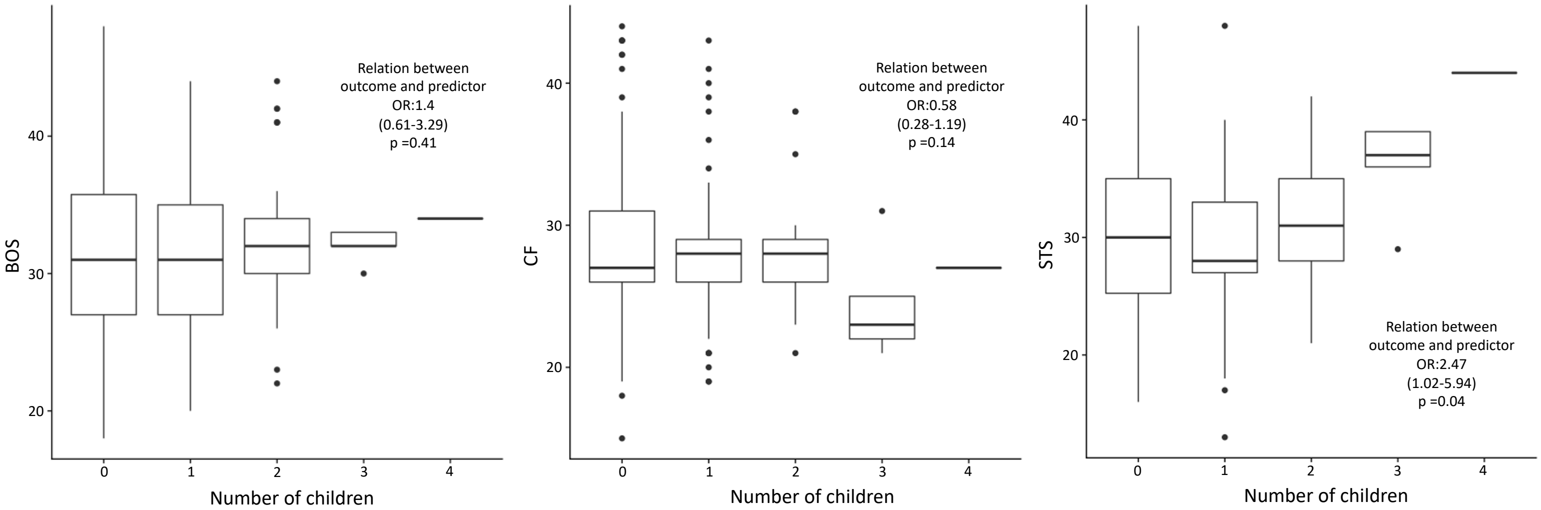

Figure 4: Univariate model of children for BOS, STS and CS

Table 10: Univariate model of children for BOS

Reference	Factor	Odds ratio	95%CI	p-value
Not have	Number of children	1.4	(0.61-3.29)	0.41

Table 11: Univariate model of children for STS

Reference	Factor	Odds ratio	95%CI	p-value
Not have	Number of children	0.58	(0.28-1.19)	0.14

Table 12: Univariate model of children for CS

Reference	Factor	Odds ratio	95%CI	p-value
Not have	Number of children	2.47	(1.02-5.94)	0.04

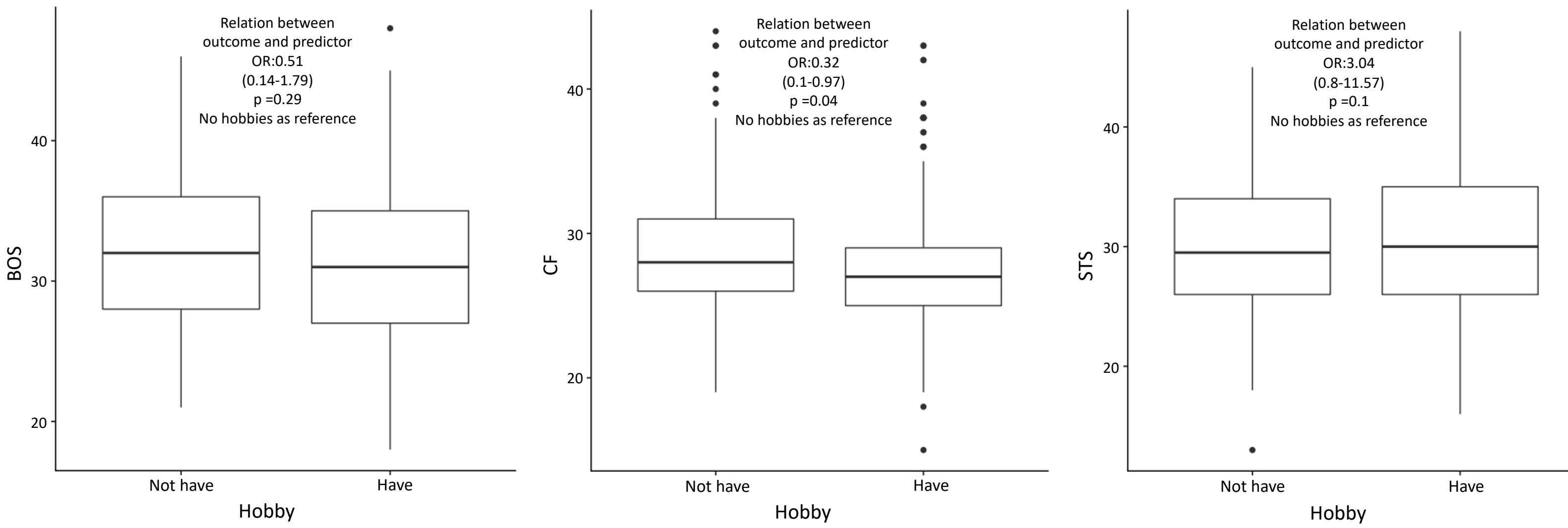

Figure 5: Univariate model of hobby for BOS, STS and CS

Table 13: Univariate model of hobby for BOS

Reference	Factor	Odds ratio	95%CI	p-value
No hobbies	Having hobby	0.51	(0.14-1.79)	0.29

Table 14: Univariate model of hobby for STS

Reference	Factor	Odds ratio	95%CI	p-value
No hobbies	Having hobby	0.32	(0.1-0.97)	0.04

Table 15: Univariate model of hobby for STS

Reference	Factor	Odds ratio	95%CI	p-value
No hobbies	Having hobby	3.04	(0.8-11.57)	0.1

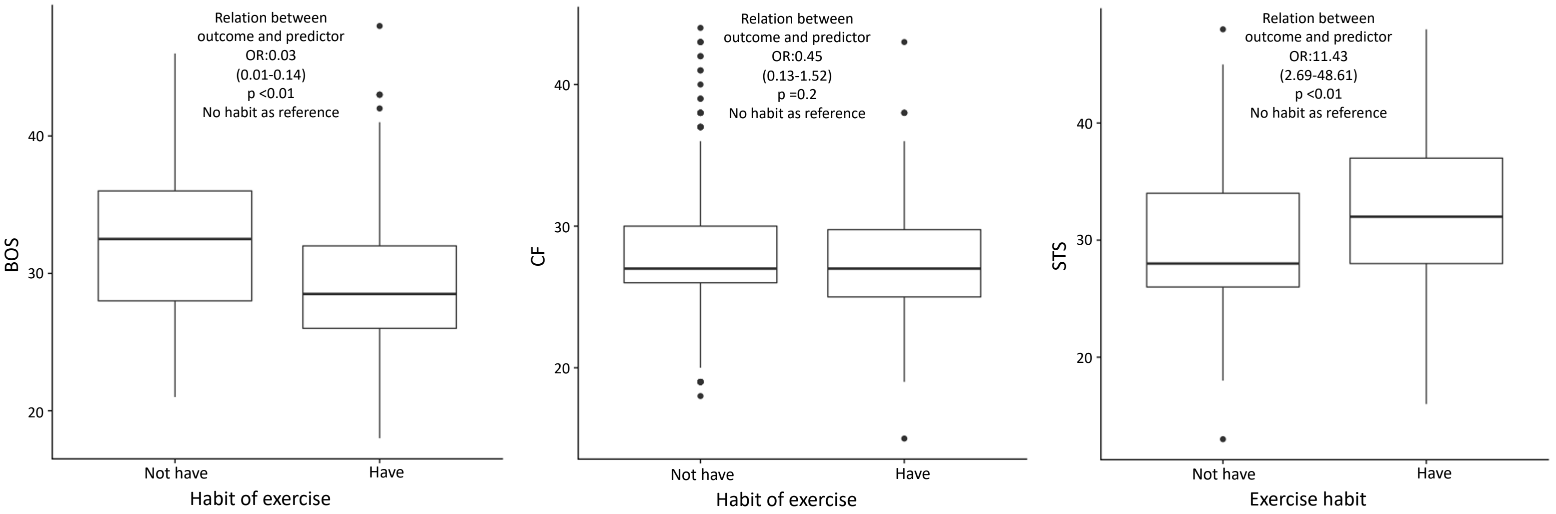

Figure 6: Univariate model of exercise for BOS, STS and CS

Table 16: Univariate model of exercise for BOS

Reference	Factor	Odds ratio	95%CI	p-value
No habit	Habit	0.03	(0.01-0.14)	>0.01

Table 17: Univariate model of exercise for STS

Reference	Factor	Odds ratio	95%CI	p-value
No habit	Habit	0.45	(0.13-1.52)	0.2

Table 18: Univariate model of exercise for CS

Reference	Factor	Odds ratio	95%CI	p-value
No habit	Habit	11.43	(2.69-48.61)	>0.01

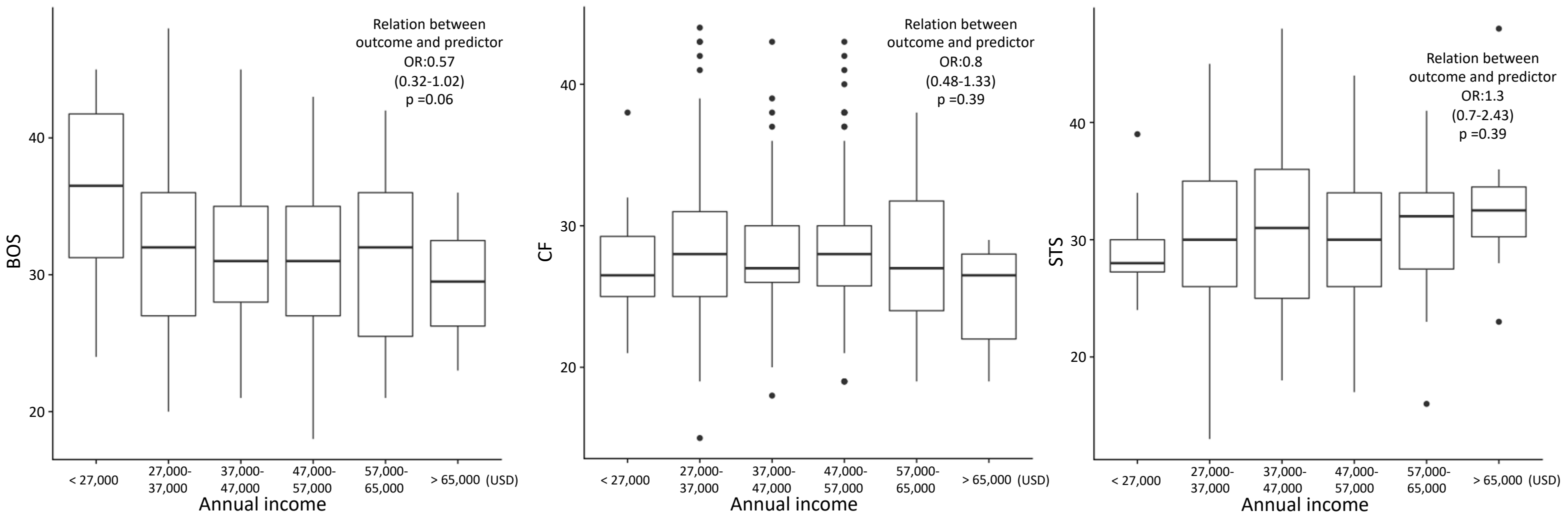

Figure 7: Univariate model of annual income for BOS, STS and CS

Table 19: Univariate model of annual income for BOS

Reference	Factor	Odds ratio	95%CI	p-value
<27,000	Degree of annual income	0.57	(0.32-1.02)	0.06

Table 20: Univariate model of annual income for STS

Reference	Factor	Odds ratio	95%CI	p-value
<27,000	Degree of annual income	0.8	(0.48-1.33)	0.39

Table 21: Univariate model of annual income for CS

Reference	Factor	Odds ratio	95%CI	p-value
<27,000	Degree of annual income	1.3	(0.7-2.43)	0.39

We categorized the annual income as follow:
<27,000 USD; 27,000-37,000 USD; 37,000-47,000 USD; 47,000-57,000 USD; 57,000-65,000 USD; >65,000 USD.

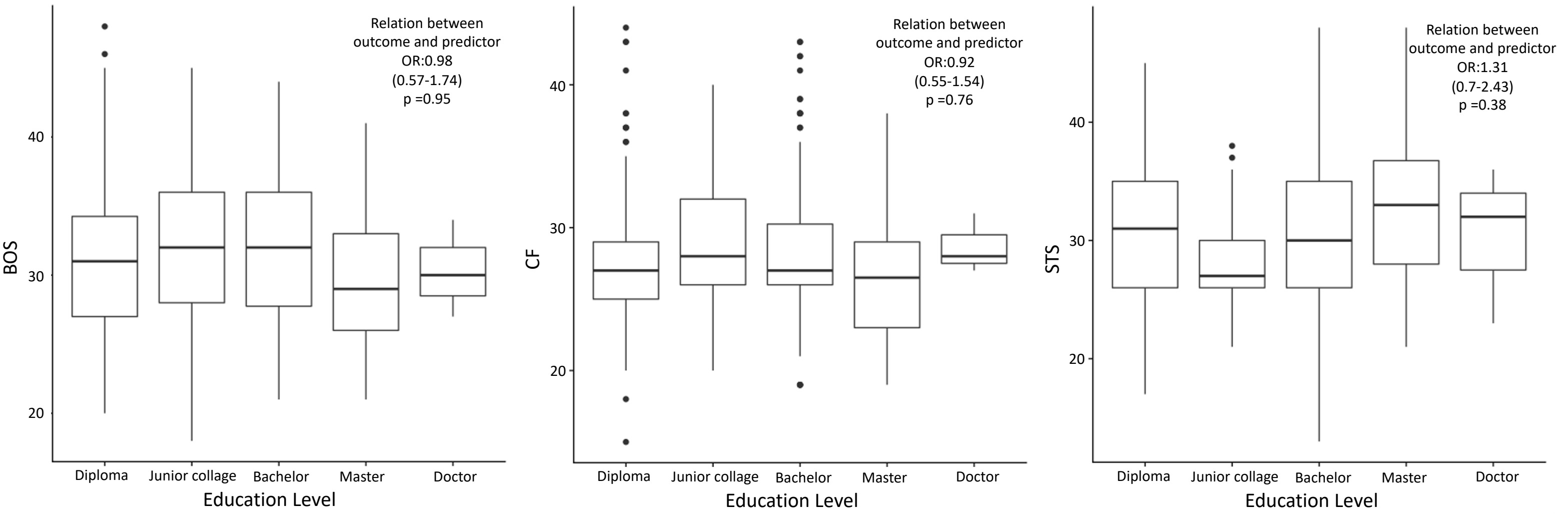

Figure 8: Univariate model of education for BOS, STS and CS

Table 22: Univariate model of education for BOS

Reference	Factor	Odds ratio	95%CI	p-value
Diploma	Degree of education level	0.98	(0.57-1.74)	0.95

Table 23: Univariate model of education for STS

Reference	Factor	Odds ratio	95%CI	p-value
Diploma	Degree of education level	0.92	(0.55-1.54)	0.76

Table 24: Univariate model of education for CS

Reference	Factor	Odds ratio	95%CI	p-value
Diploma	Degree of education level	1.31	(0.7-2.43)	0.38

We categorized the education level as follow:

Diploma; Junior college; Bachelor; Master; Doctor

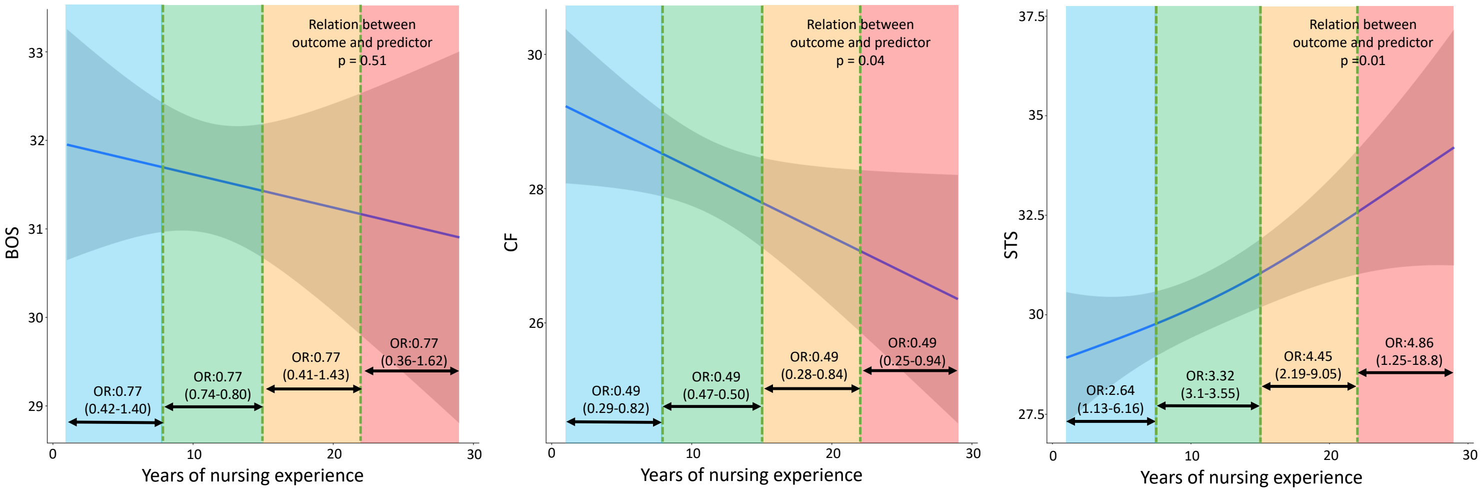

Figure 9: Univariate model of years of nursing experience for BOS, STS and CS

Table 25: Univariate model of years of nursing experience for BOS

Quartile	Range of factor	Odds ratio	95% CI
0th-25th	1-8	0.77	(0.42-1.40)
25th-50th	8-15	0.77	(0.74-0.80)
50th-75th	15-22	0.77	(0.41-1.43)
75th-100th	22-29	0.77	(0.36-1.62)

GAM modeling showed negative association and p-value was p=0.51

Table 26: Univariate model of years of nursing experience for BOS

Quartile	Range of factor	Odds ratio	95% CI
0th-25th	1-8	0.49	(0.29-0.82)
25th-50th	8-15	0.49	(0.47-0.50)
50th-75th	15-22	0.49	(0.28-0.84)
75th-100th	22-29	0.49	(0.25-0.94)

GAM modeling showed negative association and p-value was p=0.04

Table 27: Univariate model of years of nursing experience for BOS

Quartile	Range of factor	Odds ratio	95% CI
0th-25th	1-8	2.64	(1.13-6.16)
25th-50th	8-15	3.32	(3.1-3.55)
50th-75th	15-22	4.45	(2.19-9.05)
75th-100th	22-29	4.86	(1.25-18.8)

GAM modeling showed positive association and p-value was p=0.01

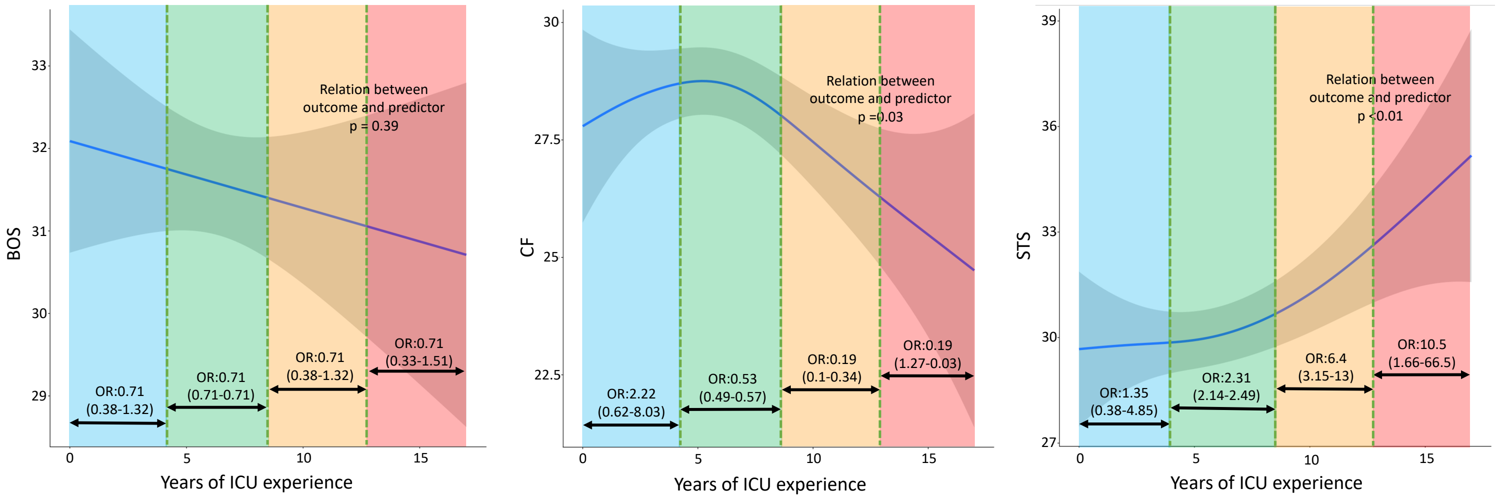

Figure 10: Univariate model of years of ICU experience for BOS, STS and CS

Table 28: Univariate model of years of ICU experience for BOS

Quartile	Range of factor	Odds ratio	95% CI
0th-25th	0-4	0.71	(0.38-1.32)
25th-50th	4-9	0.71	(0.71-0.71)
50th-75th	9-13	0.71	(0.38-1.32)
75th-100th	13-17	0.71	(0.33-1.51)

GAM modeling showed negative association and p-value was p=0.39

Table 29: Univariate model of years of ICU experience for BOS

Quartile	Range of factor	Odds ratio	95% CI
0th-25th	0-4	2.22	(0.62-8.03)
25th-50th	4-9	0.53	(0.49-0.57)
50th-75th	9-13	0.19	(0.1-0.34)
75th-100th	13-17	0.19	(0.03-1.27)

GAM modeling showed negative association and p-value was p=0.03

Table 30: Univariate model of years of ICU experience for BOS

Quartile	Range of factor	Odds ratio	95% CI
0th-25th	0-4	1.35	(0.38-4.85)
25th-50th	4-9	2.31	(2.14-2.49)
50th-75th	9-13	6.4	(3.15-13)
75th-100th	13-17	10.5	(1.66-66.5)

GAM modeling showed positive association and p-value was p<0.01

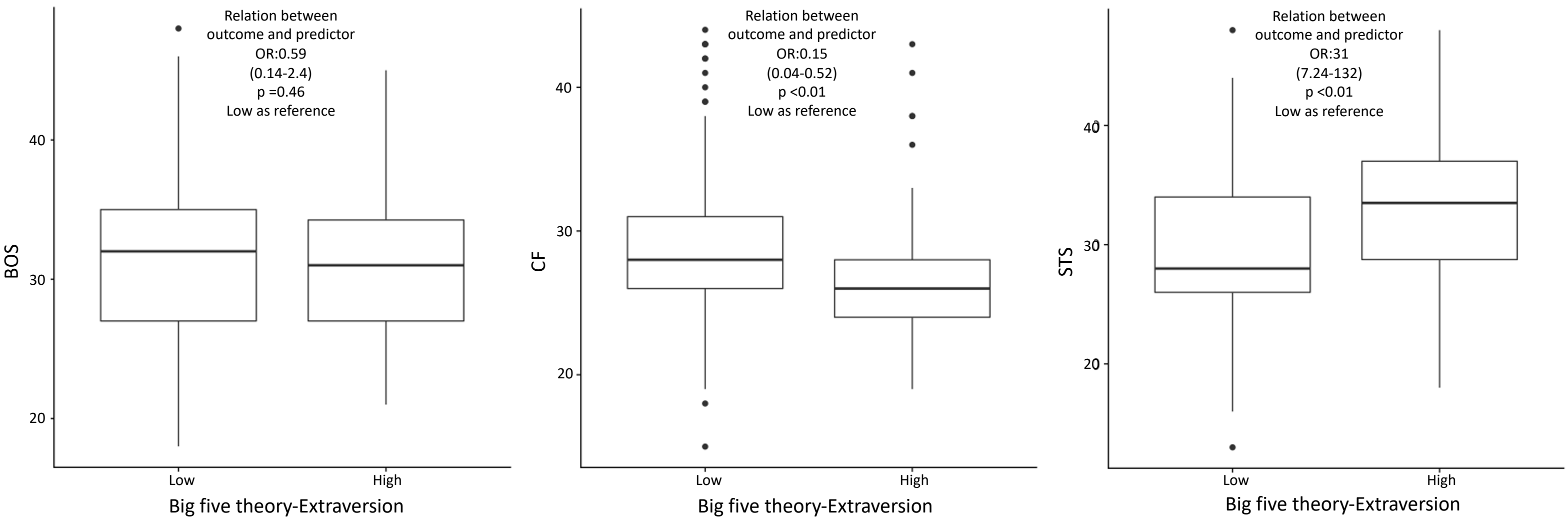

Figure 11: Univariate model of extraversion for BOS, STS and CS

Table 31: Univariate model of extraversion for BOS

Reference	Factor	Odds ratio	95%CI	p-value
Low	High	0.59	(0.14-2.4)	0.46

Table 32: Univariate model of extraversion for STS

Reference	Factor	Odds ratio	95%CI	p-value
Low	High	0.15	(0.04-0.52)	<0.01

Table 33: Univariate model of extraversion for CS

Reference	Factor	Odds ratio	95%CI	p-value
Low	High	31	(7.24-132)	<0.01

Big five theory- Agreeableness for BOS, STS, CS

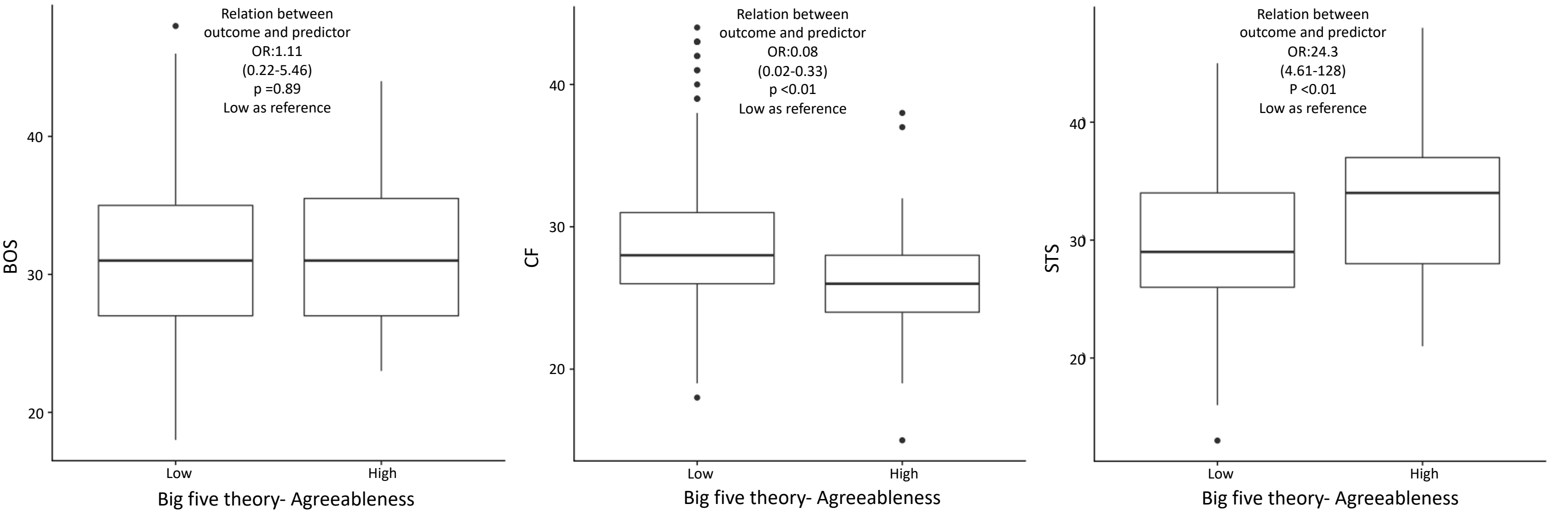

Figure 12: Univariate model of agreeableness for BOS, STS and CS

Table 34: Univariate model of agreeableness for BOS

Reference	Factor	Odds ratio	95%CI	p-value
Low	High	1.11	(0.22-5.46)	0.89

Table 35: Univariate model of agreeableness for STS

Reference	Factor	Odds ratio	95%CI	p-value
Low	High	0.08	(0.02-0.33)	<0.01

Table 36: Univariate model of agreeableness for CS

Reference	Factor	Odds ratio	95%CI	p-value
Low	High	24.3	(4.61-128)	<0.01

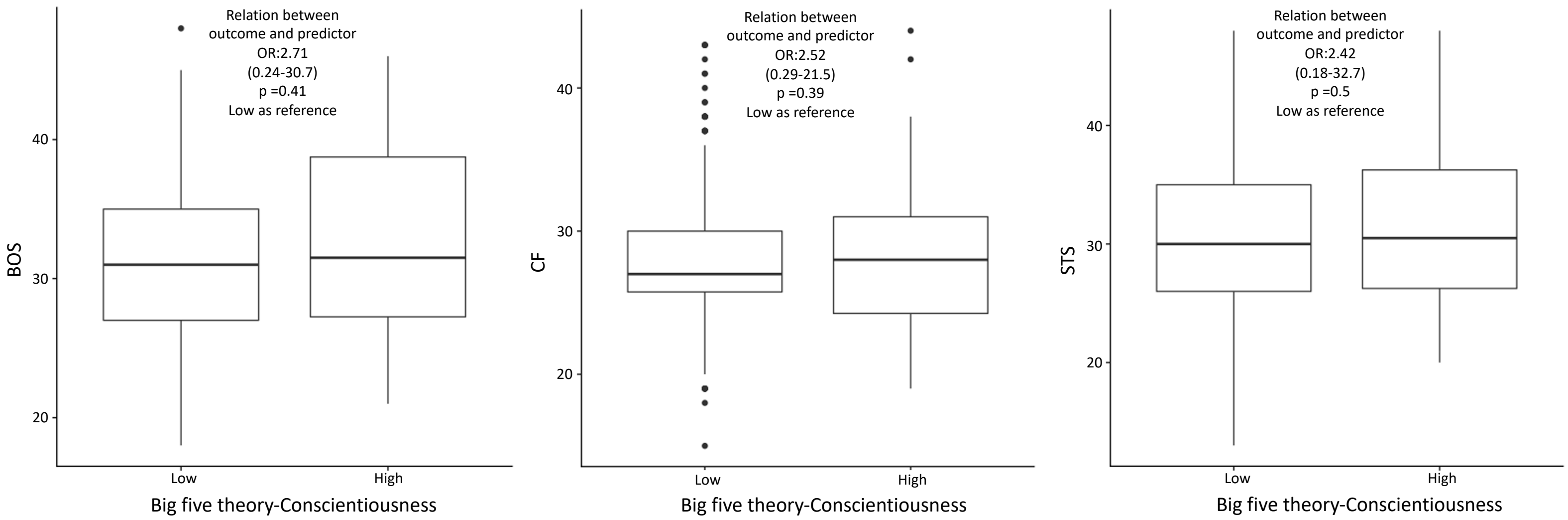

Figure 13: Univariate model of conscientiousness for BOS, STS and CS

Table 37: Univariate model of conscientiousness for BOS

Reference	Factor	Odds ratio	95%CI	p-value
Low	High	2.71	(0.24-30.7)	0.41

Table 38: Univariate model of conscientiousness for STS

Reference	Factor	Odds ratio	95%CI	p-value
Low	High	2.52	(0.29-21.5)	0.39

Table 39: Univariate model of conscientiousness for CS

Reference	Factor	Odds ratio	95%CI	p-value
Low	High	2.42	(0.18-32.7)	0.5

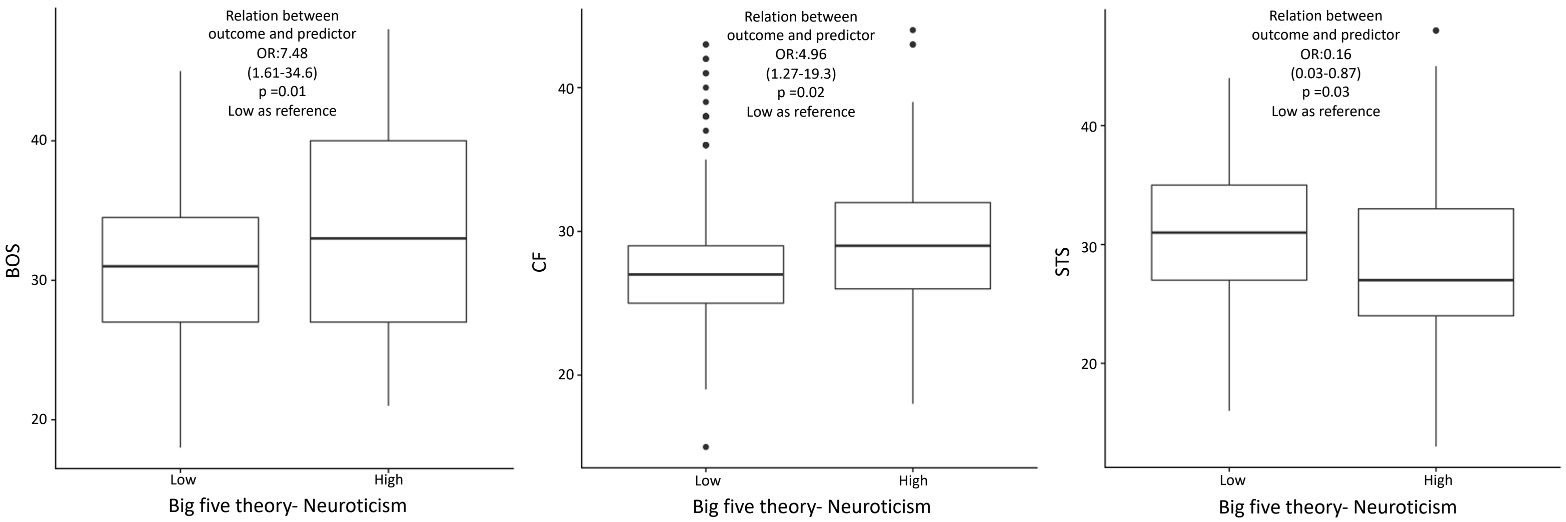

Figure 14: Univariate model of neuroticism for BOS, STS and CS

Table 40: Univariate model of neuroticism for BOS

Reference	Factor	Odds ratio	95%CI	p-value
Low	High	7.48	(1.61-34.6)	0.01

Table 41: Univariate model of neuroticism for STS

Reference	Factor	Odds ratio	95%CI	p-value
Low	High	4.96	(1.27-19.3)	0.02

Table 42: Univariate model of neuroticism for CS

Reference	Factor	Odds ratio	95%CI	p-value
Low	High	0.16	(0.03-0.87)	0.03

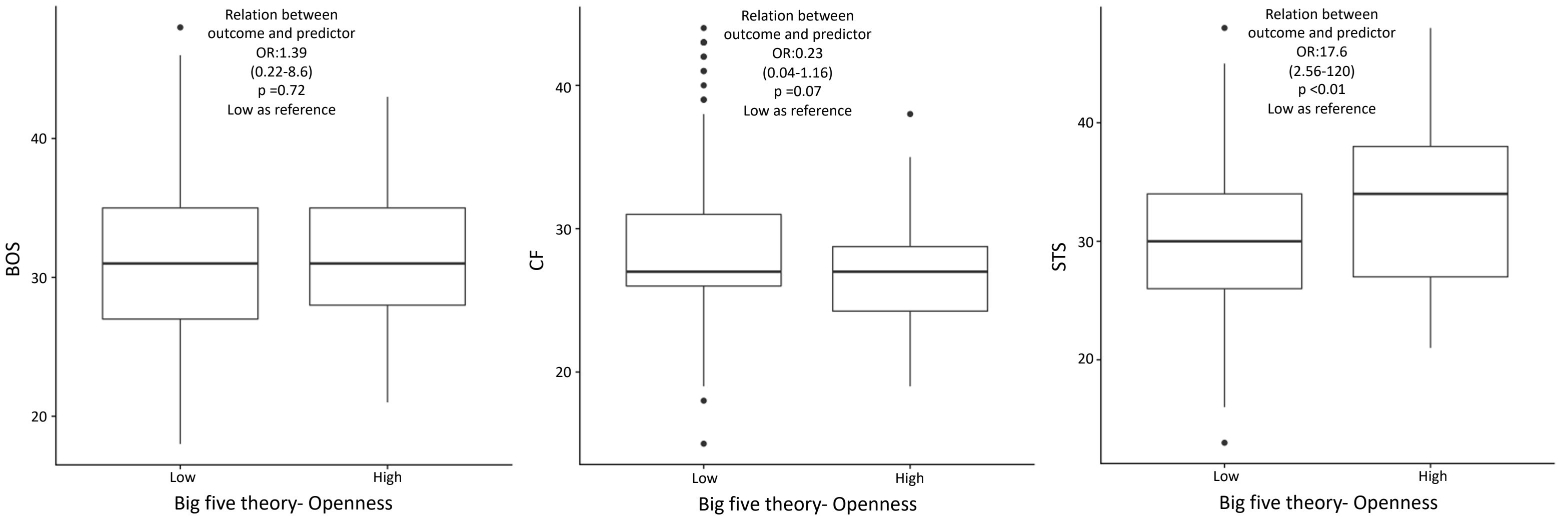

Figure 15: Univariate model of openness for BOS, STS and CS

Table 43: Univariate model of openness for BOS

Reference	Factor	Odds ratio	95%CI	p-value
Low	High	1.39	(0.22-8.6)	0.72

Table 44: Univariate model of openness for STS

Reference	Factor	Odds ratio	95%CI	p-value
Low	High	0.23	(0.04-1.16)	0.07

Table 45: Univariate model of openness for CS

Reference	Factor	Odds ratio	95%CI	p-value
Low	High	17.6	(2.56-120)	<0.01

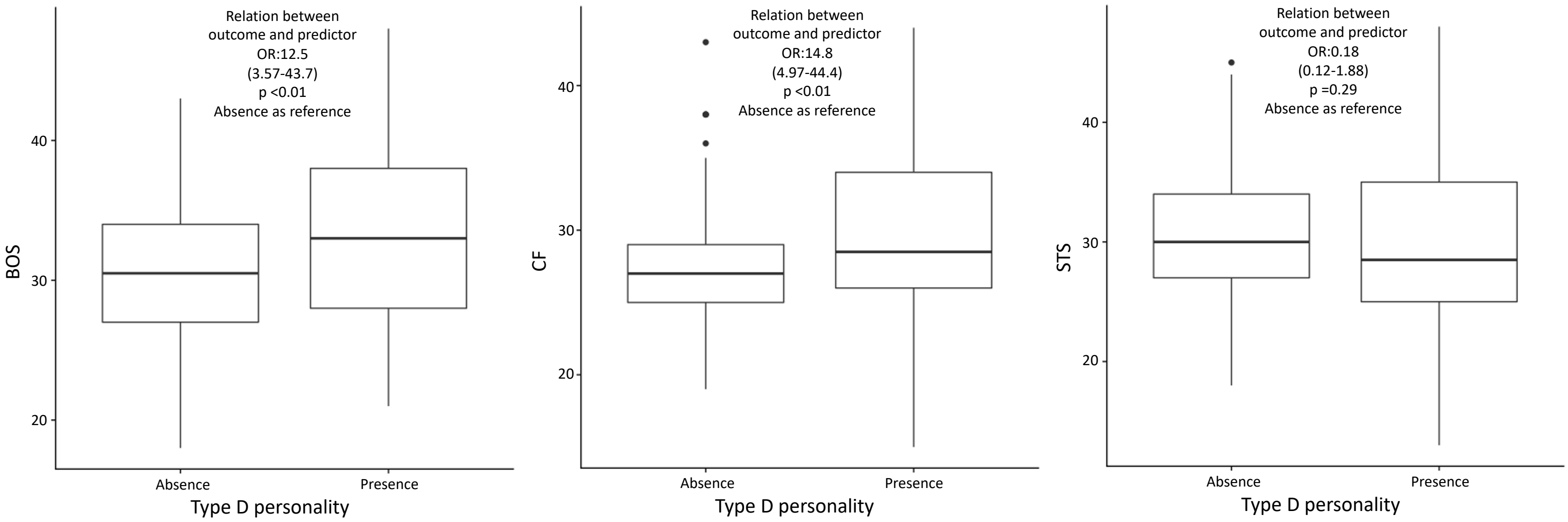

Figure 16: Univariate model of Type-D personality for BOS, STS and CS

Table 46: Univariate model of Type-D personality for BOS

Reference	Factor	Odds ratio	95%CI	p-value
Absence	Presence	12.5	(3.57-43.7)	<0.01

Table 47: Univariate model of Type-D personality for STS

Reference	Factor	Odds ratio	95%CI	p-value
Low	High	14.8	(4.97-44.4)	<0.01

Table 48: Univariate model of Type-D personality for CS

Reference	Factor	Odds ratio	95%CI	p-value
Low	High	0.18	(0.12-1.88)	0.29

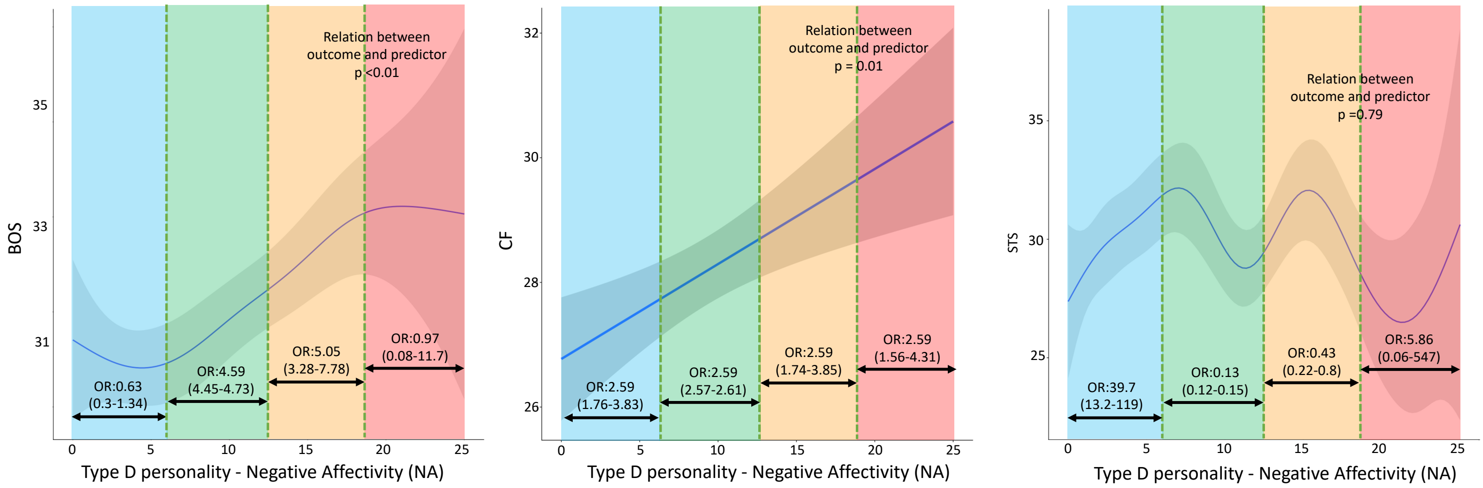

Figure 17: Univariate model of Type-D personality- Negative Affectivity (NA) for BOS, STS and CS

Table 49: Univariate model of Type-D personality- Negative Affectivity (NA) for BOS

Quartile	Range of factor	Odds ratio	95% CI
0th-25th	0-6	0.63	(0.3-1.34)
25th-50th	6-13	4.59	(4.45-4.73)
50th-75th	13-19	5.05	(3.28-7.78)
75th-100th	19-25	0.97	(0.08-11.7)

GAM modeling showed positive association and p-value was p>0.01

Table 50: Univariate model of Type-D personality- Negative Affectivity (NA) for STS

Quartile	Range of factor	Odds ratio	95% CI
0th-25th	0-6	2.59	(1.76-3.83)
25th-50th	6-13	2.59	(2.57-2.61)
50th-75th	13-19	2.59	(1.74-3.85)
75th-100th	19-25	2.59	(1.56-4.31)

GAM modeling showed positive association and p-value was p=0.01

Table 51: Univariate model of Type-D personality- Negative Affectivity (NA) for CS

Quartile	Range of factor	Odds ratio	95% CI
0th-25th	0-6	39.7	(13.2-119)
25th-50th	6-13	0.13	(0.12-0.15)
50th-75th	13-19	0.43	(0.22-0.8)
75th-100th	19-25	5.86	(0.06-547)

GAM modeling showed fluctuate association and p-value was p=0.79

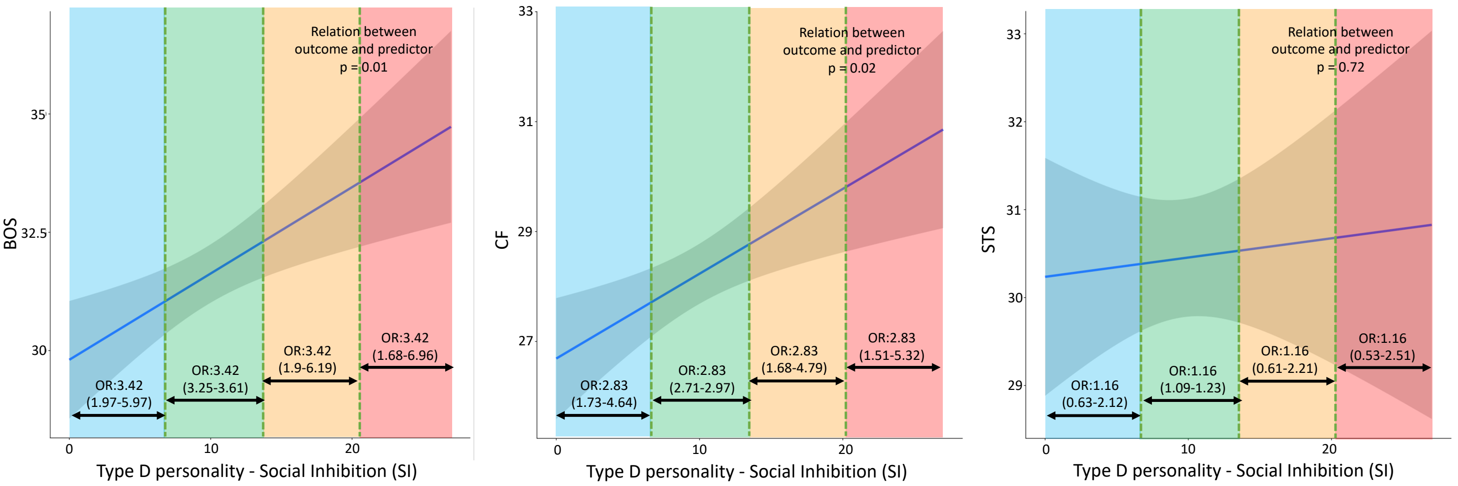

Figure 18: Univariate model of Type-D personality-Social Inhibition (SI) for BOS, STS and CS

Table 52: Univariate model of Type-D personality-Social Inhibition (SI) for BOS

Quartile	Range of factor	Odds ratio	95% CI
0th-25th	0-7	3.42	(1.97-5.97)
25th-50th	7-14	3.42	(3.25-3.61)
50th-75th	14-20	3.42	(1.9-6.19)
75th-100th	20-27	3.42	(1.68-6.96)

GAM modeling showed positive association and p-value was p=0.01

Table 53: Univariate model of Type-D personality-Social Inhibition (SI) for STS

Quartile	Range of factor	Odds ratio	95% CI
0th-25th	0-7	2.83	(1.73-4.64)
25th-50th	7-14	2.83	(2.71-2.97)
50th-75th	14-20	2.83	(1.68-4.79)
75th-100th	20-27	2.83	(1.51-5.32)

GAM modeling showed positive association and p-value was p=0.01

Table 54: Univariate model of Type-D personality-Social Inhibition (SI) for CS

Quartile	Range of factor	Odds ratio	95% CI
0th-25th	0-7	1.16	(0.63-2.12)
25th-50th	7-14	1.16	(1.09-1.23)
50th-75th	14-20	1.16	(0.61-2.21)
75th-100th	20-27	1.16	(0.53-2.51)

GAM modeling showed positive association and p-value was p=0.72

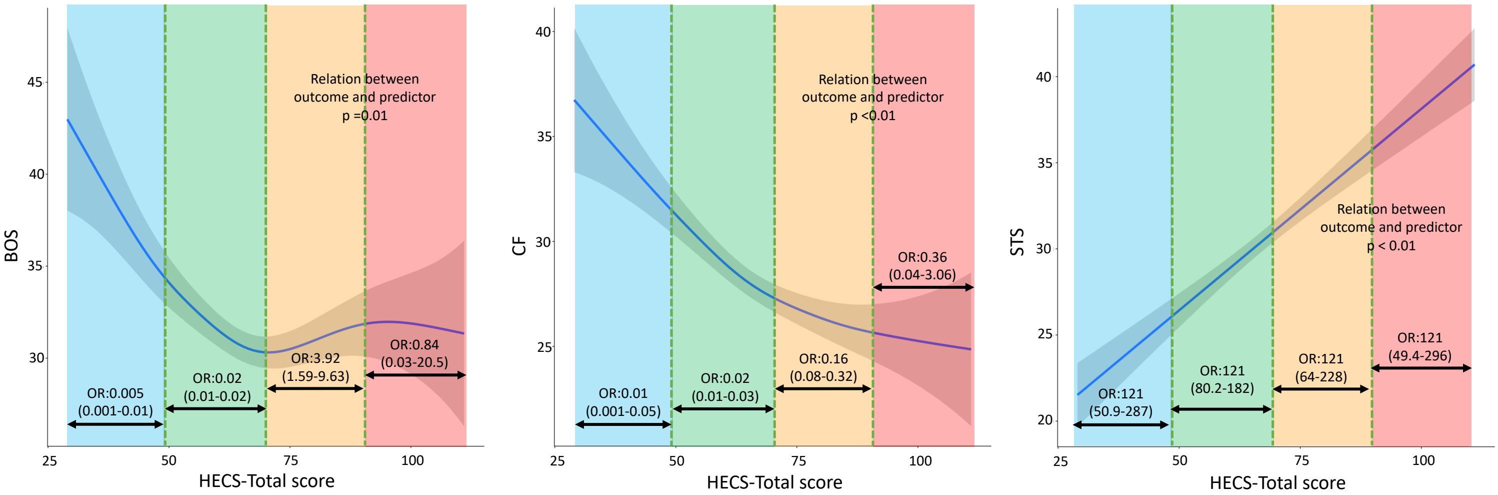

Figure 19: Univariate model of HECS-Total for BOS, STS, CS

Table 55: Univariate model of HECS-Total for BOS

Quartile	Range of factor	Odds ratio	95% CI
0th-25th	29-50	0.005	(0.001-0.01)
25th-50th	50-70	0.02	(0.01-0.02)
50th-75th	70-90	3.92	(1.59-9.63)
75th-100th	90-111	0.84	(0.03-20.5)

GAM modeling showed negative association and p-value was p=0.01

Table 56: Univariate model of HECS-Total for STS

Quartile	Range of factor	Odds ratio	95% CI
0th-25th	29-50	0.01	(0.001-0.05)
25th-50th	50-70	0.02	(0.01-0.03)
50th-75th	70-90	0.16	(0.08-0.32)
75th-100th	90-111	0.36	(0.04-3.06)

GAM modeling showed negative association and p-value was p<0.01

Table 57: Univariate model of HECS-Total for CS

Quartile	Range of factor	Odds ratio	95% CI
0th-25th	29-50	121	(50.9-287)
25th-50th	50-70	121	(80.2-182)
50th-75th	70-90	121	(64-228)
75th-100th	90-111	121	(49.4-296)

GAM modeling showed positive association and p-value was p<0.01

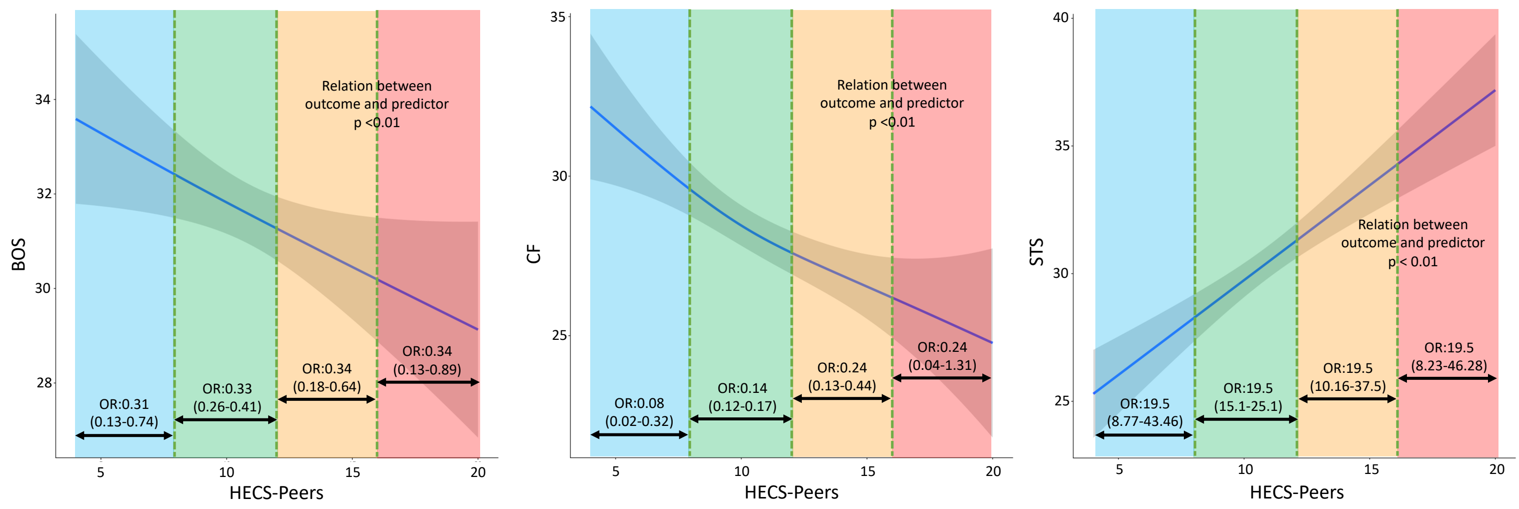

Figure 20: Univariate model of HECS- Peers for BOS, STS, CS

Table 58: Univariate model of HECS-Peers for BOS

Quartile	Range of factor	Odds ratio	95% CI
0th-25th	4-8	0.31	(0.13-0.74)
25th-50th	8-12	0.33	(0.26-0.41)
50th-75th	12-16	0.34	(0.18-0.64)
75th-100th	16-20	0.34	(0.13-0.89)

GAM modeling showed negative association and p-value was p<0.01

Table 59: Univariate model of HECS-Peers for STS

Quartile	Range of factor	Odds ratio	95% CI
0th-25th	4-8	0.08	(0.02-0.32)
25th-50th	8-12	0.14	(0.12-0.17)
50th-75th	12-16	0.24	(0.13-0.44)
75th-100th	16-20	0.24	(0.04-1.31)

GAM modeling showed negative association and p-value was p<0.01

Table 60: Univariate model of HECS-Peers for CS

Quartile	Range of factor	Odds ratio	95% CI
0th-25th	4-8	19.5	(8.77-43.46)
25th-50th	8-12	19.5	(15.1-25.1)
50th-75th	12-16	19.5	(10.16-37.5)
75th-100th	16-20	19.5	(8.23-46.28)

GAM modeling showed positive association and p-value was p<0.01

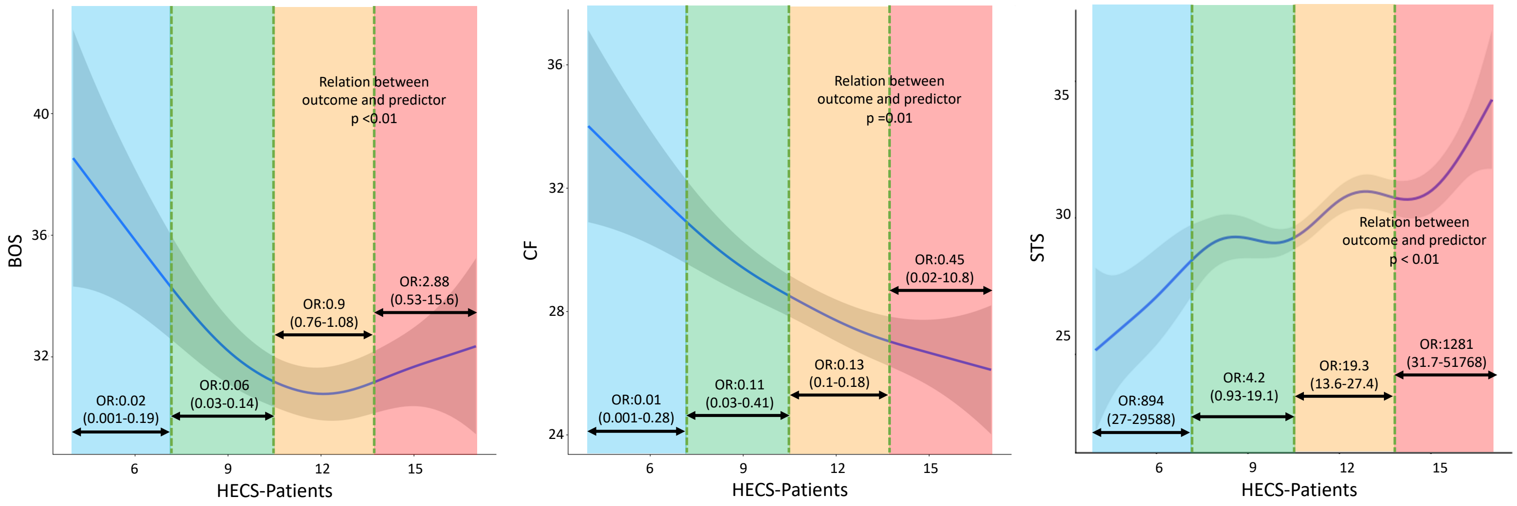

Figure 21: Univariate model of HECS- Patients for BOS, STS, CS

Table 61: Univariate model of HECS- Patients for BOS

Quartile	Range of factor	Odds ratio	95% CI
0th-25th	4-7	0.02	(0.001-0.19)
25th-50th	7-11	0.06	(0.03-0.14)
50th-75th	11-14	0.9	(0.76-1.08)
75th-100th	14-17	2.88	(0.53-15.6)

GAM modeling showed negative association and p-value was $p<0.01$

Table 62: Univariate model of HECS- Patients for STS

Quartile	Range of factor	Odds ratio	95% CI
0th-25th	4-7	0.01	(0.001-0.28)
25th-50th	7-11	0.11	(0.03-0.41)
50th-75th	11-14	0.13	(0.1-0.18)
75th-100th	14-17	0.45	(0.02-10.8)

GAM modeling showed negative association and p-value was $p=0.01$

Table 63: Univariate model of HECS- Patients for CS

Quartile	Range of factor	Odds ratio	95% CI
0th-25th	4-7	894	(27-29588)
25th-50th	7-11	4.2	(0.93-19.1)
50th-75th	11-14	19.3	(13.6-27.4)
75th-100th	14-17	1281	(31.7-51768)

GAM modeling showed positive association and p-value was $p<0.01$

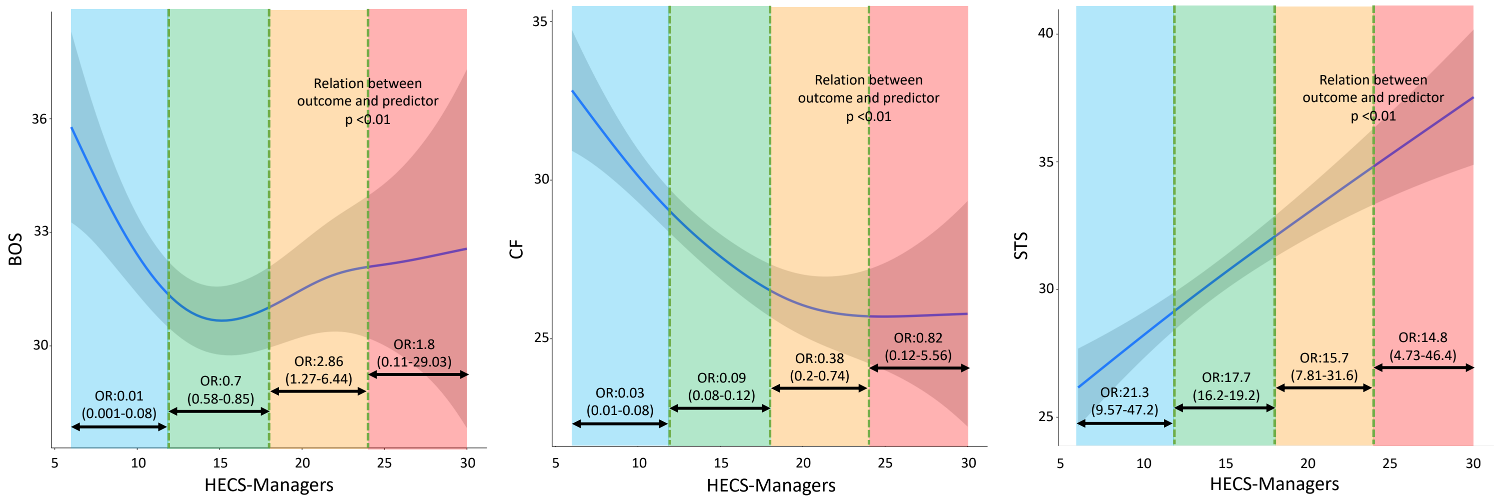

Figure 22: Univariate model of HECS- Managers for BOS, STS, CS

Table 64: Univariate model of HECS- Managers for BOS

Quartile	Range of factor	Odds ratio	95% CI
0th-25th	6-12	0.01	(0.001-0.08)
25th-50th	12-18	0.7	(0.58-0.85)
50th-75th	18-24	2.86	(1.27-6.44)
75th-100th	24-30	1.8	(0.11-29.03)

GAM modeling showed negative association and p-value was p<0.01

Table 65: Univariate model of HECS- Managers for STS

Quartile	Range of factor	Odds ratio	95% CI
0th-25th	6-12	0.03	(0.01-0.08)
25th-50th	12-18	0.09	(0.08-0.12)
50th-75th	18-24	0.38	(0.2-0.74)
75th-100th	24-30	0.82	(0.12-5.56)

GAM modeling showed negative association and p-value was p<0.01

Table 66: Univariate model of HECS- Managers for CS

Quartile	Range of factor	Odds ratio	95% CI
0th-25th	6-12	21.3	(9.57-47.2)
25th-50th	12-18	17.7	(16.2-19.2)
50th-75th	18-24	15.7	(7.81-31.6)
75th-100th	24-30	14.8	(4.73-46.4)

GAM modeling showed positive association and p-value was p<0.01

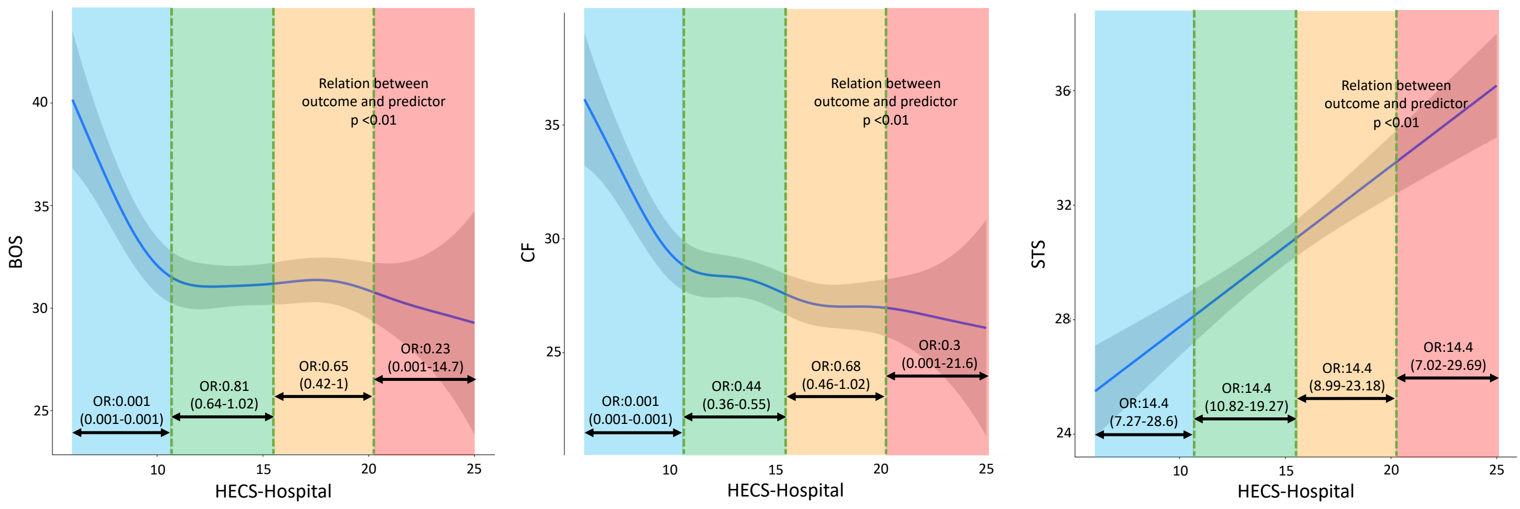

Figure 23: Univariate model of HECS- Hospital for BOS, STS, CS

Table 67: Univariate model of HECS- Hospital for BOS

Quartile	Range of factor	Odds ratio	95% CI
0th-25th	6-11	0.001	(0.001-0.001)
25th-50th	11-16	0.81	(0.64-1.02)
50th-75th	16-20	0.65	(0.42-1)
75th-100th	20-25	0.23	(0.001-14.7)

GAM modeling showed negative association and p-value was p<0.01

Table 68: Univariate model of HECS- Hospital for STS

Quartile	Range of factor	Odds ratio	95% CI
0th-25th	6-11	0.001	(0.001-0.001)
25th-50th	11-16	0.44	(0.36-0.55)
50th-75th	16-20	0.68	(0.46-1.02)
75th-100th	20-25	0.3	(0.001-21.6)

GAM modeling showed positive association and p-value was p<0.01

Table 69: Univariate model of HECS- Hospital for CS

Quartile	Range of factor	Odds ratio	95% CI
0th-25th	6-11	14.4	(7.27-28.6)
25th-50th	11-16	14.4	(10.82-19.27)
50th-75th	16-20	14.4	(8.99-23.18)
75th-100th	20-25	14.4	(7.02-29.69)

GAM modeling showed positive association and p-value was p<0.01

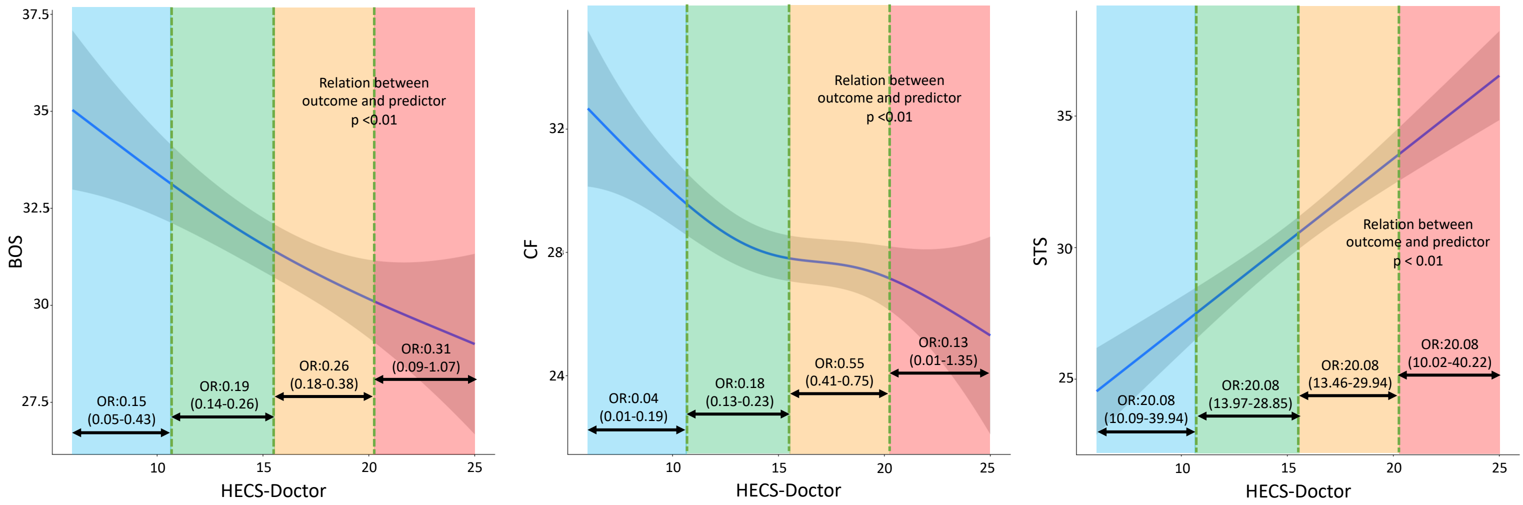

Figure 24: Univariate model of HECS- Doctors for BOS, STS, CS

Table 70: Univariate model of HECS- Doctors for BOS

Quartile	Range of factor	Odds ratio	95% CI
0th-25th	6-11	0.15	(0.05-0.43)
25th-50th	11-16	0.19	(0.14-0.26)
50th-75th	16-20	0.26	(0.18-0.38)
75th-100th	20-25	0.31	(0.09-1.07)

GAM modeling showed negative association and p-value was p<0.01

Table 71: Univariate model of HECS- Doctors for STS

Quartile	Range of factor	Odds ratio	95% CI
0th-25th	6-11	0.04	(0.01-0.19)
25th-50th	11-16	0.18	(0.13-0.23)
50th-75th	16-20	0.55	(0.41-0.75)
75th-100th	20-25	0.13	(0.01-1.35)

GAM modeling showed negative association and p-value was p<0.01

Table 72: Univariate model of HECS- Doctors for CS

Quartile	Range of factor	Odds ratio	95% CI
0th-25th	6-11	20.08	(10.09-39.94)
25th-50th	11-16	20.08	(13.97-28.85)
50th-75th	16-20	20.08	(13.46-29.94)
75th-100th	20-25	20.08	(10.02-40.22)

GAM modeling showed positive association and p-value was p<0.01

Figure legends

Figure 1: Univariate model of age for BOS, STS and CS

This figure shows the results of univariate model of age for burnout syndrome (BOS), Secondary traumatic stress (STS) and compassion satisfaction (CS).

Figure 2: Univariate model of sex for BOS, STS and CS

This figure shows the results of univariate model of sex for burnout syndrome (BOS), secondary traumatic stress (STS) and compassion satisfaction (CS).

Figure 3: Univariate model of marital status for BOS, STS and CS

This figure shows the results of univariate model of marital status for burnout syndrome (BOS), secondary traumatic stress (STS) and compassion satisfaction (CS).

Figure 4: Univariate model of children for BOS, STS and CS

This figure shows the results of univariate model of children for burnout syndrome (BOS), secondary traumatic stress (STS) and compassion satisfaction (CS).

Figure 5: Univariate model of hobby for BOS, STS and CS

This figure shows the results of univariate model of hobby for burnout syndrome (BOS), secondary traumatic stress (STS) and compassion satisfaction (CS).

Figure 6: Univariate model of exercise for BOS, STS and CS

This figure shows the results of univariate model of exercise for burnout syndrome (BOS), secondary traumatic stress (STS) and compassion satisfaction (CS).

Figure 7: Univariate model of annual income for BOS, STS and CS

This figure shows the results of univariate model of annual income for burnout syndrome (BOS), secondary traumatic stress (STS) and compassion satisfaction (CS).

Figure 8: Univariate model of education for BOS, STS and CS

This figure shows the results of univariate model of education for burnout syndrome (BOS), secondary traumatic stress (STS) and compassion satisfaction (CS).

Figure 9: Univariate model of years of nursing experience for BOS, STS and CS
This figure shows the results of univariate model of years of nursing experience for burnout syndrome (BOS), secondary traumatic stress (STS) and compassion satisfaction (CS).

Figure 10: Univariate model of ICU experience for BOS, STS and CS
This figure shows the results of univariate model of ICU experience for burnout syndrome (BOS), secondary traumatic stress (STS) and compassion satisfaction (CS).

Figure 11: Univariate model of extraversion for BOS, STS and CS
This figure shows the results of univariate model of extraversion for burnout syndrome (BOS), secondary traumatic stress (STS) and compassion satisfaction (CS).

Figure 12: Univariate model of agreeableness for BOS, STS and CS
This figure shows the results of univariate model of agreeableness for burnout syndrome (BOS), secondary traumatic stress (STS) and compassion satisfaction (CS).

Figure 13: Univariate model of conscientiousness for BOS, STS and CS
This figure shows the results of univariate model of conscientiousness for burnout syndrome (BOS), secondary traumatic stress (STS) and compassion satisfaction (CS).

Figure 14: Univariate model of neuroticism for BOS, STS and CS
This figure shows the results of univariate model of neuroticism for burnout syndrome (BOS), secondary traumatic stress (STS) and compassion satisfaction (CS).

Figure 15: Univariate model of openness for BOS, STS and CS
This figure shows the results of univariate model of openness for burnout syndrome (BOS), secondary traumatic stress (STS) and compassion satisfaction (CS).

Figure 16: Univariate model of Type-D personality for BOS, STS and CS
This figure shows the results of univariate model of Type-D personality for burnout syndrome (BOS), secondary traumatic stress (STS) and compassion satisfaction (CS).

Figure 17: Univariate model of Type-D personality- Negative Affectivity (NA) for BOS, STS and CS
This figure shows the results of univariate model of Type-D personality- Negative Affectivity (NA) for burnout syndrome (BOS), secondary traumatic stress (STS) and compassion satisfaction (CS).

Figure 18: Univariate model of Type-D personality-Social Inhibition (SI) for BOS, STS and CS
This figure shows the results of univariate model of Type-D personality-Social Inhibition (SI) for burnout syndrome (BOS), secondary traumatic stress (STS) and compassion satisfaction (CS).

Figure 19: Univariate model of HECS-Total for BOS, STS, CS
This figure shows the results of univariate model of hospital ethical climate survey (HECS)- Total for burnout syndrome (BOS), secondary traumatic stress (STS) and compassion satisfaction (CS).

Figure 20: Univariate model of HECS-Peers for BOS, STS, CS
This figure shows the results of univariate model of hospital ethical climate survey (HECS)- Peers for burnout syndrome (BOS), secondary traumatic stress (STS) and compassion satisfaction (CS).

Figure 21: Univariate model of HECS-Patients for BOS, STS, CS
This figure shows the results of univariate model of hospital ethical climate survey (HECS)- Patients for burnout syndrome (BOS), secondary traumatic stress (STS) and compassion satisfaction (CS).

Figure 22: Univariate model of HECS-Managers for BOS, STS, CS
This figure shows the results of univariate model of hospital ethical climate survey (HECS)- Managers for burnout syndrome (BOS), secondary traumatic stress (STS) and compassion satisfaction (CS).

Figure 23: Univariate model of HECS-Hospital for BOS, STS, CS
This figure shows the results of univariate model of hospital ethical climate survey (HECS)- Hospital for burnout syndrome (BOS), secondary traumatic stress (STS) and compassion satisfaction (CS).

Figure 24: Univariate model of HECS-Doctors for BOS, STS, CS
This figure shows the results of univariate model of hospital ethical climate survey (HECS)- Doctors for burnout syndrome (BOS), secondary traumatic stress (STS) and compassion satisfaction (CS).
