# Supplementary material for: PERSonality, Ehical, and PROfessional quality of life in Pediatric/Adult Intensive Nurses study: PERSEPRO PAIN study
Source: PLoS One. 2022 Mar 7;17(3):e0259721. doi: 10.1371/journal.pone.0259721 (PMC8901072; doi:10.1371/journal.pone.0259721)
Supplement: S2 Data — (PDF) [file pone.0259721.s002.pdf]

Online Data Supplement 2: Result of multivariate analysis for BOS, STS, CS

PERSonality, Ehical, and PROfessional quality of life in Pediatric/Adult Intensive Nurses study: PERSEPRO PAIN study

Yujiro Matsuishi[1†], Bryan J. Mathis[2†], Haruhiko Hoshino[3], Yuki Enomoto [4,5], Nobutake Shimojo[5], Satoru Kawano[5], Hideaki Sakuramoto[6], Yoshiaki Inoue[5]

1 Neuroscience Nursing, St. Luke's International University, Tokyo, Japan

2 International Medical Center, University of Tsukuba Hospital, Tsukuba, Ibaraki, Japan.

3 Adult Health Nursing, Department of Nursing, International University of Health and Welfare, Narita, Japan

4University of Tsukuba Hospital, Department of Pediatrics, Tsukuba, Ibaraki, Japan.

5Department of Emergency and Critical Care Medicine, Faculty of Medicine, University of Tsukuba, Tsukuba, Ibaraki, Japan.

6Adult Health Nursing, College of Nursing, Ibaraki Christian University, Hitachi, Ibaraki, Japan

[†]These authors contributed equally to this study.

Running title: PERSE PRO PAIN study

Corresponding author's contact information.

Dr. Yoshiaki Inoue

Department of Emergency and Critical Care Medicine, Faculty of Medicine, University of Tsukuba, Tsukuba, Ibaraki, 305-8575, Japan.

Phone: +81-29-853-5633.

FAX: +81-29-853-3092

E-mail: yinoue@md.tsukuba.ac.jp

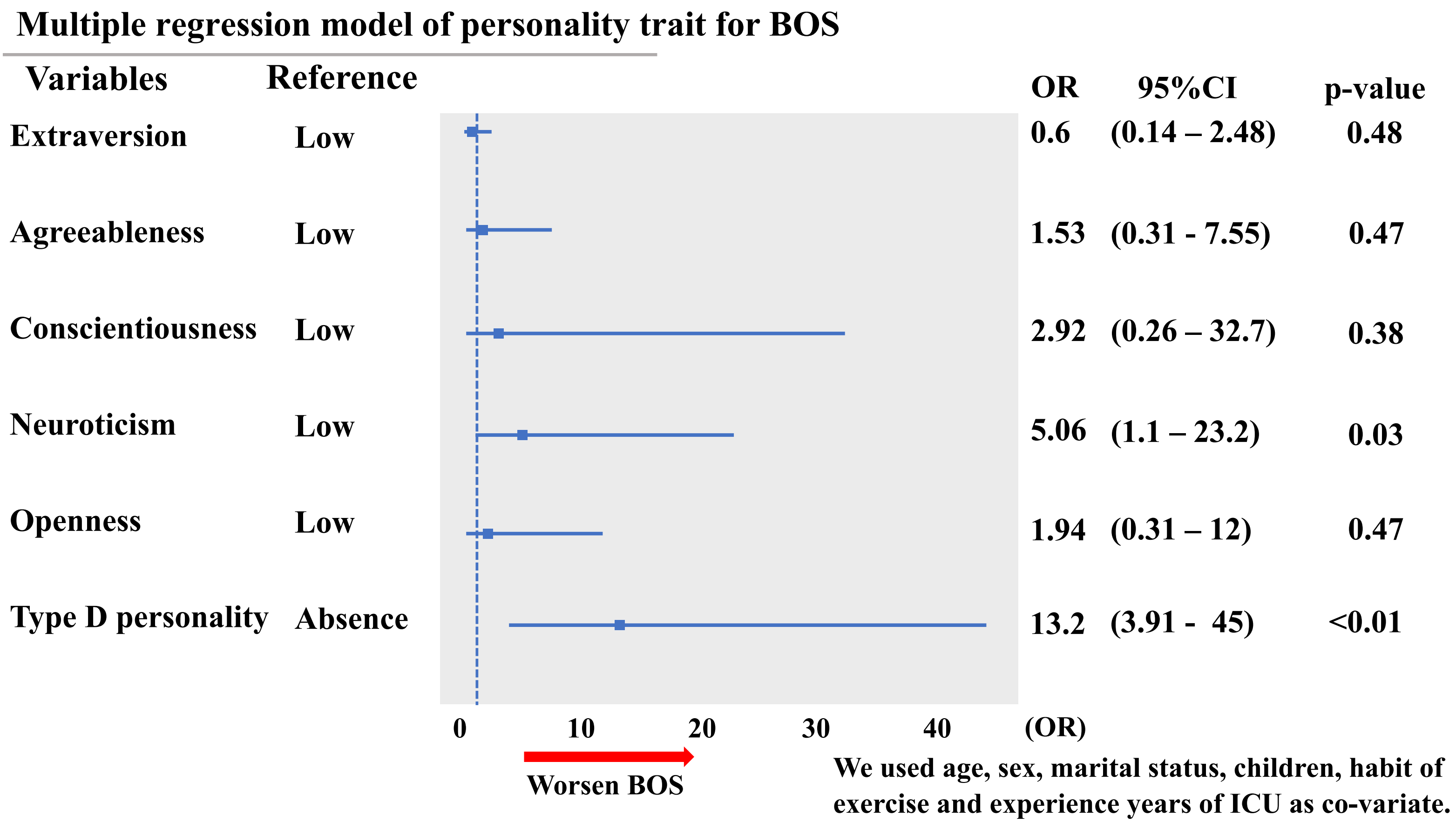

Figure 1: Multiple regression model of personality trait for BOS

Table 1: Result of extraversion for BOS in multiple regression

Variables	Odds ratio	95% CI	p-value
Age	GAM modeling: negative association		0.83
Sex	1.65	(0.4-6.71)	0.48
Marital status	0.53	(0.12-2.3)	0.4
Children	1.76	(0.63-4.88)	0.27
Exercise	0.03	(0.009-0.15)	<0.01
Years of ICU experience	GAM modeling: negative association		0.35
Extraversion	0.6	(0.14-2.48)	0.48

Table 2: Result of Agreeableness for BOS in multiple regression

Variables	Odds ratio	95% CI	p-value
Age	GAM modeling: negative association		0.7
Sex	1.48	(0.35-6.17)	0.54
Marital status	0.55	(0.12-2.36)	0.41
Children	1.72	(0.62-4.78)	0.3
Exercise	0.03	(0.009-0.14)	<0.01
Years of ICU experience	GAM modeling: negative association		0.44
Agreeableness	1.53	(0.31-7.55)	0.47

Table 3: Result of Conscientiousness for BOS in multiple regression

Variables	Odds ratio	95% CI	p-value
Age	GAM modeling: negative association		0.61
Sex	1.57	(0.38-6.38)	0.52
Marital status	0.55	(0.13-2.56)	0.48
Children	1.71	(0.61-4.72)	0.3
Exercise	0.03	(0.009-0.14)	<0.01
Years of ICU experience	GAM modeling: negative association		0.46
conscientiousness	2.92	(0.26-32.7)	0.38

Table 4: Result of Neuroticism for BOS in multiple regression

Variables	Odds ratio	95% CI	p-value
Age	GAM modeling: negative association		0.61
Sex	1.66	(0.41-6.71)	0.47
Marital status	0.58	(0.13-2.47)	0.46
Children	1.82	(0.66-5.02)	0.24
Exercise	0.04	(0.01-0.18)	<0.01
Years of ICU experience	GAM modeling: negative association		0.36
Neuroticism	5.06	(1.1-23.2)	0.03

Table 5: Result of Openness for BOS in multiple regression

Variables	Odds ratio	95% CI	p-value
Age	GAM modeling: negative association		0.66
Sex	1.53	(0.37-6.25)	0.54
Marital status	0.54	(0.12-2.33)	0.41
Children	1.7	(0.61-4.7)	0.3
Exercise	0.03	(0.01-0.14)	<0.01
Years of ICU experience	GAM modeling: negative association		0.49
Openness	1.94	(0.31-12)	0.47

Table 6: Result of Type D personality for BOS in multiple regression

Variables	Odds ratio	95% CI	p-value
Age	GAM modeling: negative association		0.49
Sex	1.77	(0.45-6.94)	0.4
Marital status	0.58	(0.14-2.42)	0.46
Children	1.7	(0.63-4.59)	0.29
Exercise	0.03	(0.009-0.14)	<0.01
Years of ICU experience	GAM modeling: negative association		0.39
Type D personality	13.2	(3.91-45)	<0.01

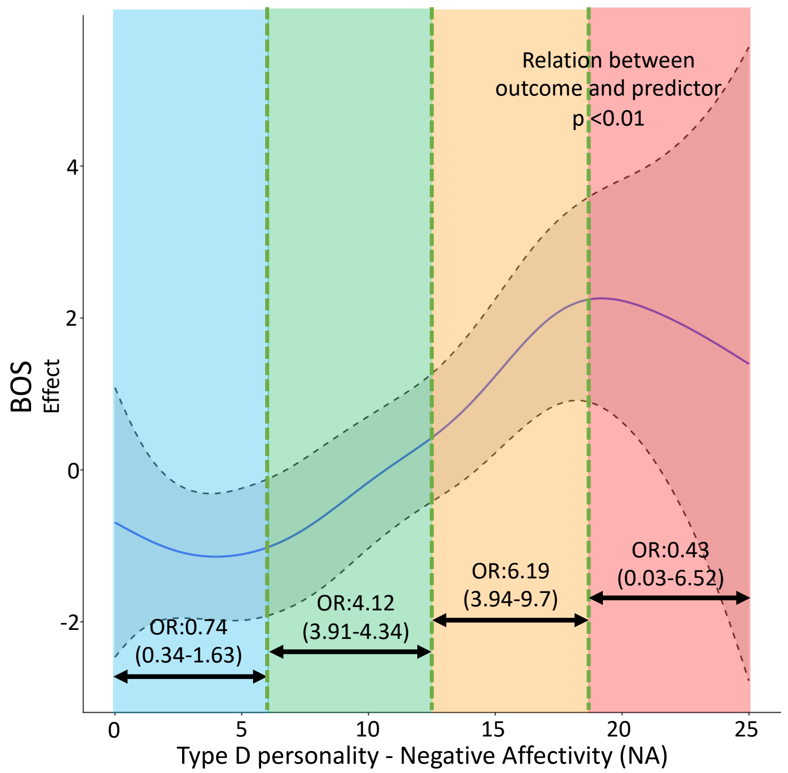

Figure 2: Multiple regression model of Type D personality - Negative Affectivity (NA) for BOS

Table 7: Result of Type D personality - Negative Affectivity (NA) for BOS in multiple regression

Variables	Odds ratio	95% CI	p-value
Age	GAM modeling: negative association		0.63
Sex	1.71	(0.43-6.81)	0.44
Marital status	0.8	(0.18-3.39)	0.76
Children	1.37	(0.49-3.8)	0.54
Exercise	0.03	(0.009-0.13)	<0.01
Years of ICU experience	GAM modeling: negative association		0.38
Type D personality- Negative Affectivity (NA)	GAM modeling: positive association		<0.01
	0th-25th 0.74	(0.34-1.63)	
	25th-50th 4.12	(3.91-4.34)	
	50th -75th 6.19	(3.94-9.7)	
	75th-100th 0.43	(0.03-6.52)	

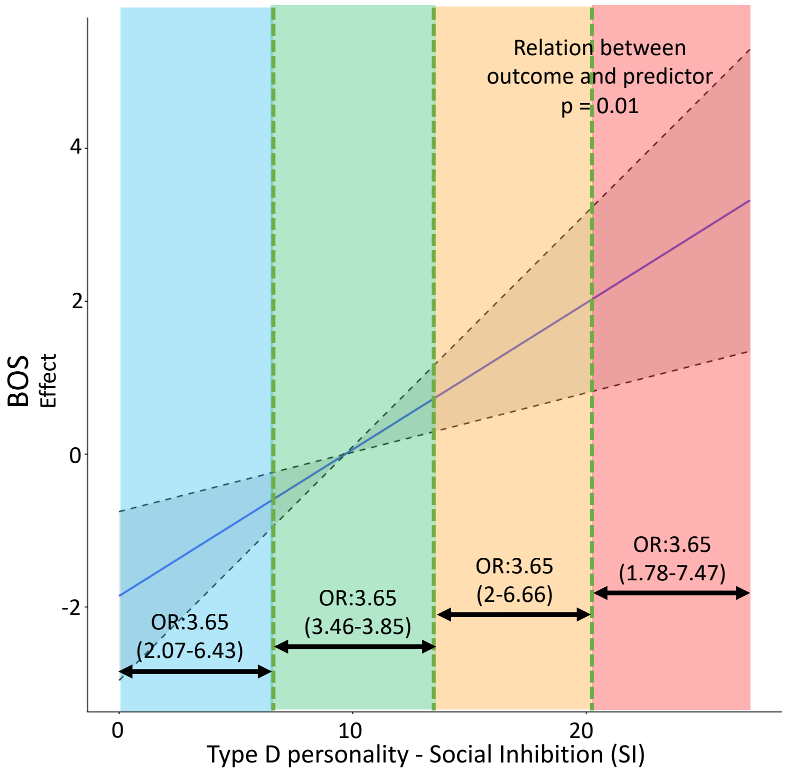

Figure 3: Multiple regression model of Type D personality -Social Inhibition (SI) for BOS

Table 8: Result of Type-D personality -Social Inhibition (SI) for BOS in multiple regression

**Variables**	**Odds ratio**	**95% CI**	**p-value**
Age	GAM modeling: negative association		0.28
Sex	1.43	(0.36-5.67)	0.61
Marital status	0.67	(0.16-2.84)	0.59
Children	1.54	(0.56-4.19)	0.39
Exercise	0.03	(0.009-0.14)	<0.01
Years of ICU experience	GAM modeling: negative association		0.54
Type D personality-Social Inhibition (SI)	GAM modeling: positive association<br>0th-25th 3.65<br>25th-50th 3.65<br>50th-75th 3.65<br>75th-100th 3.65	 (2.07-6.43)<br>(3.46-3.85)<br>(2-6.66)<br>(1.78-7.47)	<0.01

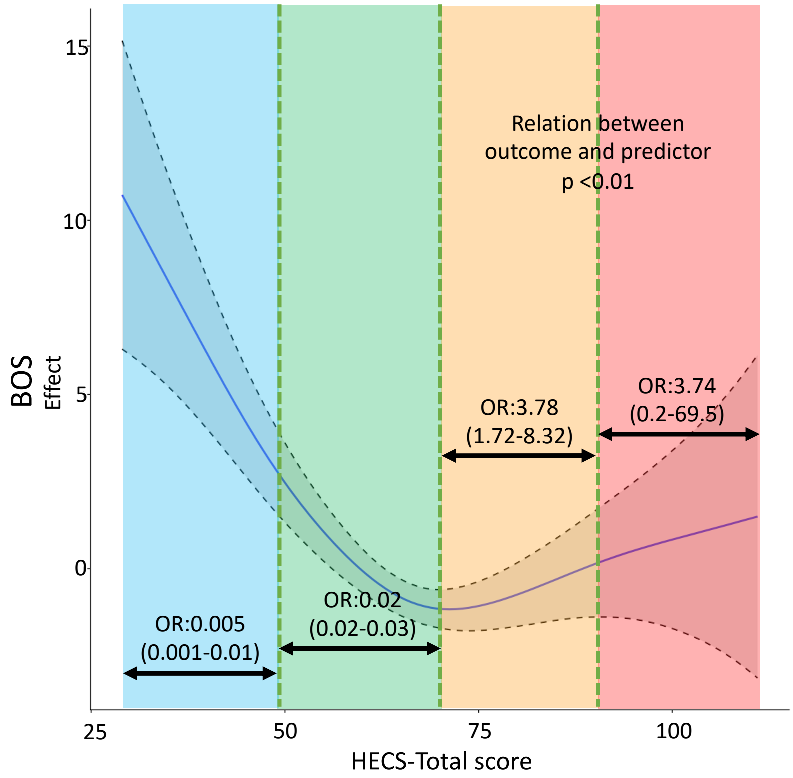

Figure 4: Multiple regression model of HECS-Total for BOS

Table 9: Result of HECS-Total for BOS in multiple regression

Variables	Odds ratio	95% CI	p-value
Age	GAM modeling: negative association		0.74
Sex	1.38	(0.36-5.31)	0.63
Marital status	0.43	(0.1-1.74)	0.24
Children	1.68	(0.63-4.46)	0.29
Exercise	0.04	(0.01-0.15)	<0.01
Years of ICU experience	GAM modeling: negative association		0.84
HECS-Total	GAM modeling: negative association<br>0th-25th 0.005<br>25th-50th 0.02<br>50th -75th 3.78<br>75th-100th 3.74	 (0.001-0.01)<br>(0.02-0.03)<br>(1.72-8.32)<br>(0.2-69.6)	<0.01

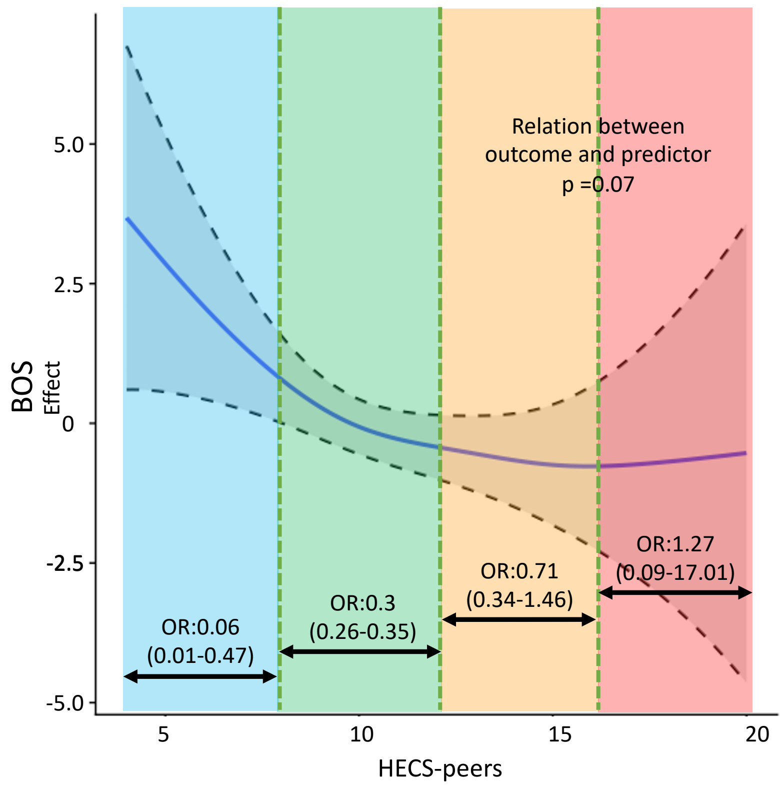

Figure 5: Multiple regression model of HECS-peers for BOS

Table 10: Result of HECS –peers for BOS in multiple regression

Variables	Odds ratio	95% CI	p-value
Age	GAM modeling: negative association		0.83
Sex	1.67	(0.41-6.74)	0.47
Marital status	0.52	(0.12-2.23)	0.38
Children	1.7	(0.62-4.66)	0.3
Exercise	0.03	(0.009-0.14)	<0.01
Years of ICU experience	GAM modeling: negative association		0.46
HECS-Peers	GAM modeling: negative association<br>0th-25th 0.06<br>25th-50th 0.3<br>50th-75th 0.71<br>75th-100th 1.24	 (0.01-0.47)<br>(0.26-0.35)<br>(0.34-1.46)<br>(0.09-17)	0.07

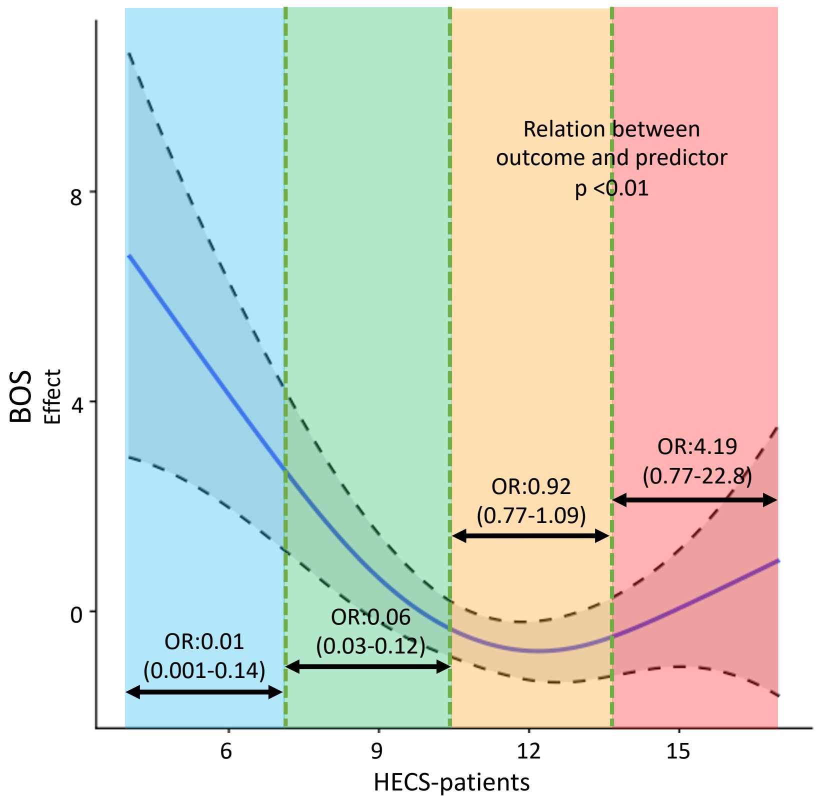

Figure 6: Multiple regression model of HECS–patients for BOS

Table 11: Result of HECS–patients for BOS in multiple regression

Variables	Odds ratio	95% CI	p-value
Age	GAM modeling: negative association		0.67
Sex	1.68	(0.43-6.58)	0.45
Marital status	0.39	(0.09-1.68)	0.21
Children	2.11	(0.78-5.73)	0.14
Exercise	0.04	(0.01-0.15)	<0.01
Years of ICU experience	GAM modeling: negative association		0.4
HECS-Patients	GAM modeling: negative association<br>0th-25th 0.01<br>25th-50th 0.06<br>50th -75th 0.92<br>75th-100th 4.19	 (0.001-0.14)<br>(0.03-0.12)<br>(0.77-1.09)<br>(0.77-22.8)	<0.01

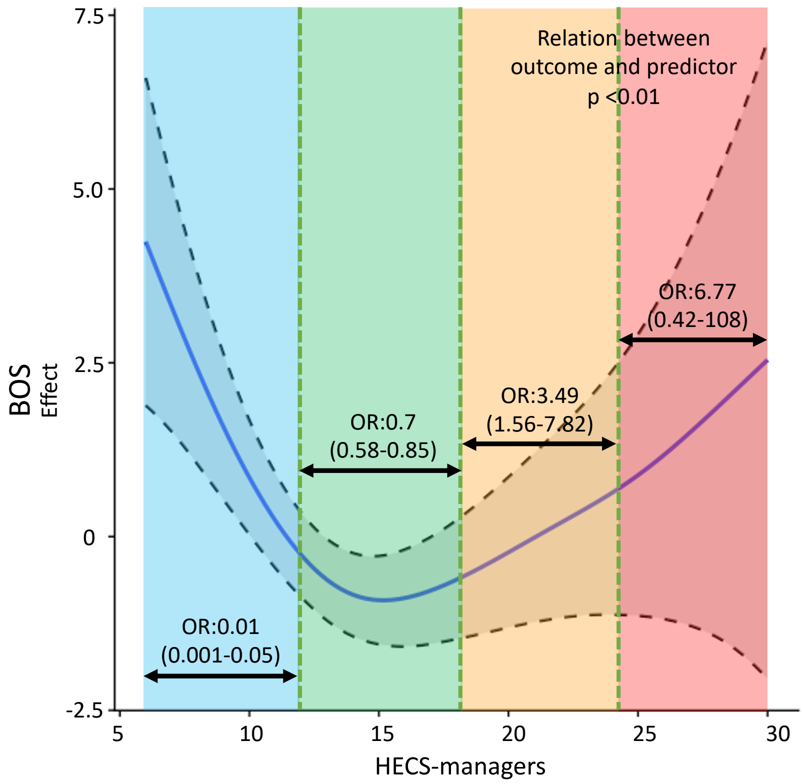

Figure 7: Multiple regression model of HECS−Managers for BOS

Table 12: Result of HECS−Managers for BOS in multiple regression

Variables	Odds ratio	95% CI	p-value
Age	GAM modeling: negative association		0.69
Sex	1.41	(0.35-5.62)	0.62
Marital status	0.55	(0.13-2.28)	0.41
Children	1.68	(0.62-4.53)	0.3
Exercise	0.03	(0.008-0.12)	<0.01
Years of ICU experience	GAM modeling: negative association		0.59
HECS-Managers	GAM modeling: negative association<br>0th-25th 0.01<br>25th-50th 0.7<br>50th -75th 3.49<br>75th-100th 6.77	 (0.001-0.05)<br>(0.58-0.85)<br>(1.56-7.82)<br>(0.42-108)	<0.01

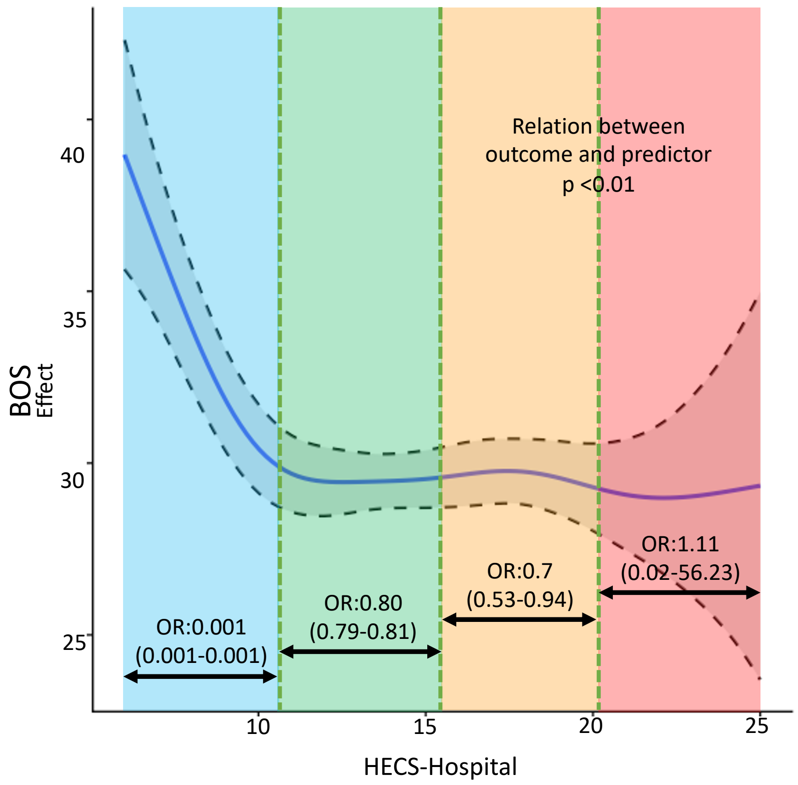

Figure 8: Multiple regression model of HECS–Hospital for BOS

Table:13 Result of HECS –Hospital for BOS in multiple regression

**Variables**	**Odds ratio**	**95% CI**	**p-value**
Age	GAM modeling: negative association		0.33
Sex	1.92	(0.43-6.82)	0.43
Marital status	0.36	(0.11-1.91)	0.29
Children	1.54	(0.57-4.17)	0.39
Exercise	0.03	(0.01-0.14)	<0.01
Years of ICU experience	GAM modeling: negative association		0.97
HECS- Hospital	GAM modeling: negative association		<0.01
	0th-25th 0.001	(0.001-0.001)	
	25th-50th 0.8	(0.79-0.81)	
	50th -75th 0.7	(0.53-0.94)	
	75th-100th 1.11	(0.02-56.23)	

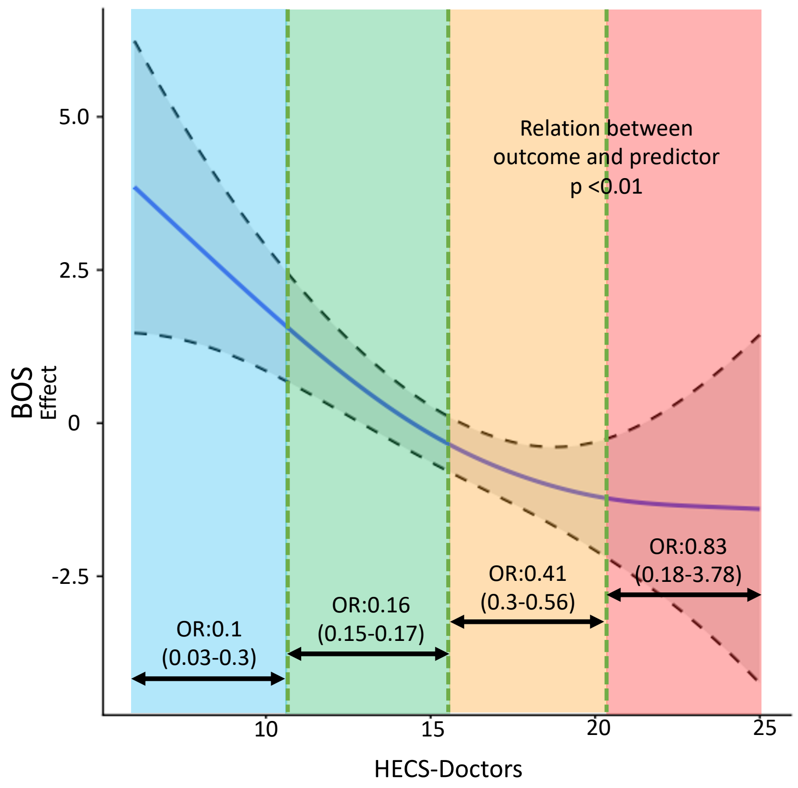

Figure 9: Multiple regression model of HECS–Doctors for BOS

Table:14 Result of HECS –Doctors for BOS in multiple regression

Variables	Odds ratio	95% CI	p-value
Age	GAM modeling: negative association		0.33
Sex	1.92	(0.43-6.82)	0.43
Marital status	0.36	(0.11-1.91)	0.29
Children	1.54	(0.57-4.17)	0.39
Exercise	0.03	(0.01-0.14)	<0.01
Years of ICU experience	GAM modeling: negative association		0.97
HECS- Doctors	GAM modeling: negative association<br>0th-25th 0.1<br>25th-50th 0.16<br>50th-75th 0.41<br>75th-100th 0.83	 (0.03-0.3)<br>(0.15-0.17)<br>(0.3-0.56)<br>(0.18-3.78)	<0.01

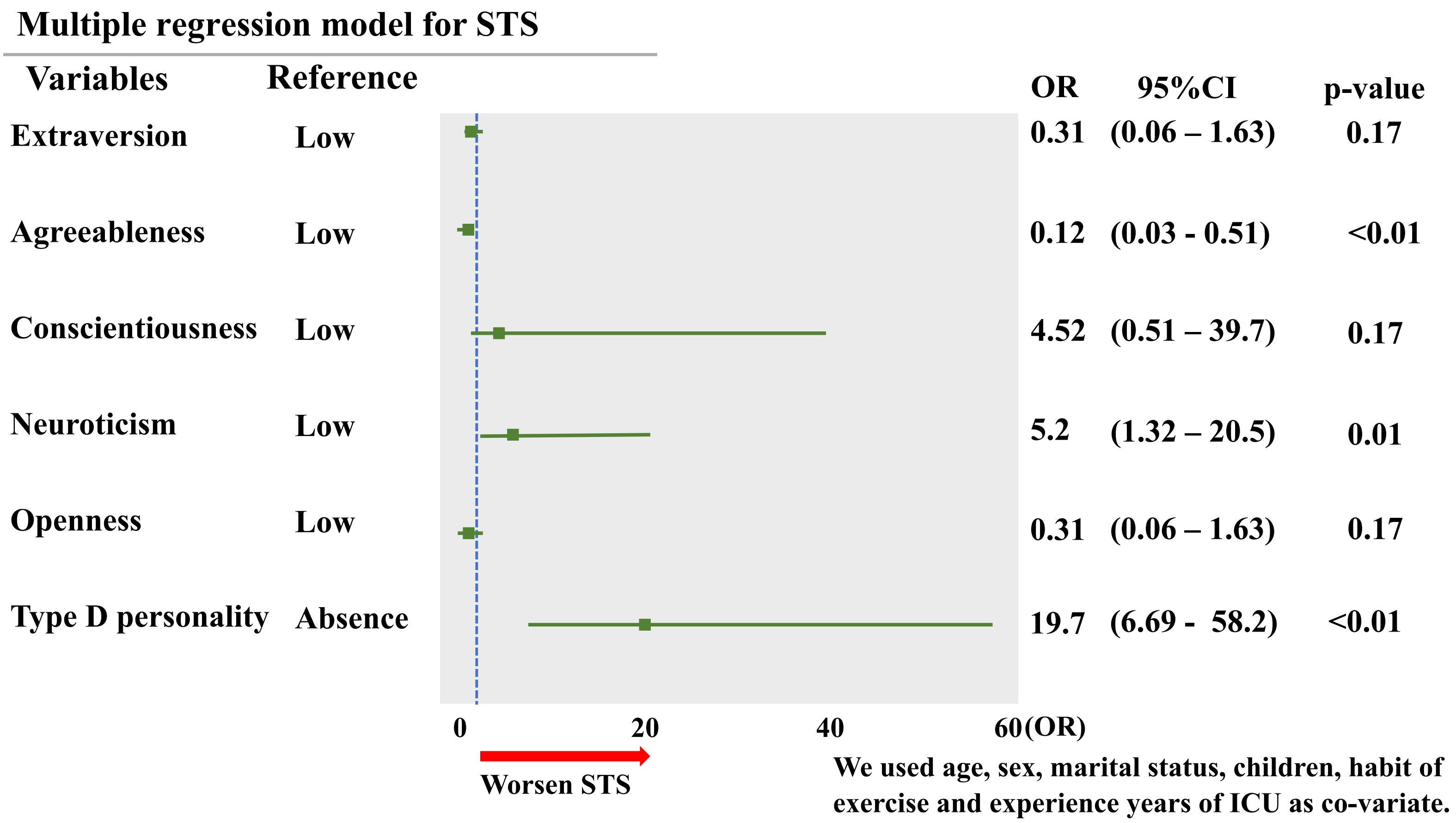

Figure 10: Multiple regression model of personality trait for STS

Table 15: Result of extraversion for STS in multiple regression

Variables	Odds ratio	95% CI	p-value
Age	GAM modeling: negative association		0.4
Sex	0.29	(0.08-1.04)	0.06
Marital status	1.42	(0.38-5.28)	0.59
Children	0.92	(0.37-2.31)	0.87
Exercise	0.58	(0.17-1.98)	0.38
Years of ICU experience	GAM modeling: negative association		0.08
Extraversion	0.31	(0.06-1.63)	0.17

Table 16: Result of Agreeableness for STS in multiple regression

Variables	Odds ratio	95% CI	p-value
Age	GAM modeling: negative association		0.34
Sex	0.37	(0.1-1.34)	0.13
Marital status	1.43	(0.38-5.26)	0.58
Children	0.88	(0.35-2.04)	0.79
Exercise	0.6	(0.18-2.04)	0.42
Years of ICU experience	GAM modeling: negative association		0.05
Agreeableness	0.12	(0.03-0.51)	<0.01

Table 17: Result of Conscientiousness for STS in multiple regression

Variables	Odds ratio	95% CI	p-value
Age	GAM modeling: negative association		0.26
Sex	0.27	(0.07-0.97)	<0.01
Marital status	1.49	(0.39-5.56)	0.04
Children	0,9	(0.36-2.24)	0.82
Exercise	0.53	(0.15-1.81)	0.31
Years of ICU experience	GAM modeling: negative association		0.1
conscientiousness	4.52	(0.5-39.7)	0.17

Table 18: Result of Neuroticism for STS in multiple regression

Variables	Odds ratio	95% CI	p-value
Age	GAM modeling: negative association		0.27
Sex	0.28	(0.08-1)	0.05
Marital status	1.38	(0.37-5.1)	0.62
Children	0.96	(0.38-2.39)	0.93
Exercise	0.66	(0.19-2.27)	0.51
Years of ICU experience	GAM modeling: negative association		0.05
Neuroticism	5.2	(1.32-20.5)	0.01

Table 19: Result of Openness for STS in multiple regression

Variables	Odds ratio	95% CI	p-value
Age	GAM modeling: negative association		0.4
Sex	0.29	(0.08-1.04)	0.06
Marital status	1.42	(0.38-5.28)	0.59
Children	0.92	(0.37-2.31)	0.87
Exercise	0.58	(0.17-1.98)	0.38
Years of ICU experience	GAM modeling: negative association		0.08
Openness	0.31	(0.06-1.63)	0.17

Table 20: Result of Type-D personality for STS in multiple regression

Variables	Odds ratio	95% CI	p-value
Age	GAM modeling: negative association		0.15
Sex	0.3	(0.08-1.01)	0.05
Marital status	1.33	(0.75-2.33)	0.65
Children	0.9	(0.37-2.18)	0.83
Exercise	0.53	(0.16-1.72)	0.29
Years of ICU experience	GAM modeling: negative association		0.03
Type D personality	19.7	(6.69-58.2)	<0.01

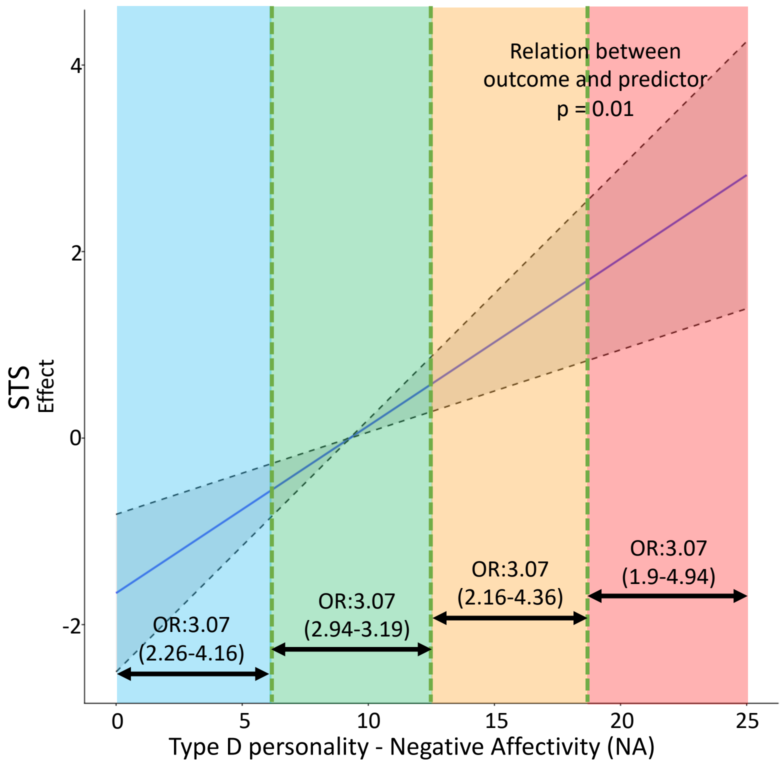

Figure 11: Multiple regression model of Type D personality - Negative Affectivity (NA) for STS

Table 21: Result of Type D personality - Negative Affectivity (NA) for STS in multiple regression

Variables	Odds ratio	95% CI	p-value
Age	GAM modeling: negative association		0.23
Sex	0.3	(0.08-1.04)	0.05
Marital status	0.36	(0.52-6.96)	0.33
Children	0.68	(0.27-1.69)	0.41
Exercise	0.49	(0.14-1.63)	0.24
Years of ICU experience	GAM modeling: negative association		0.06
Type D personality-Negative Affectivity (NA)	GAM modeling: positive association<br>0th-25th 3.07<br>25th-50th 3.07<br>50th -75th 3.07<br>75th-100th 3.07	 (2.26-4.16)<br>(2.94-3.19)<br>(2.16-4.36)<br>(1.9-4.94)	<0.01

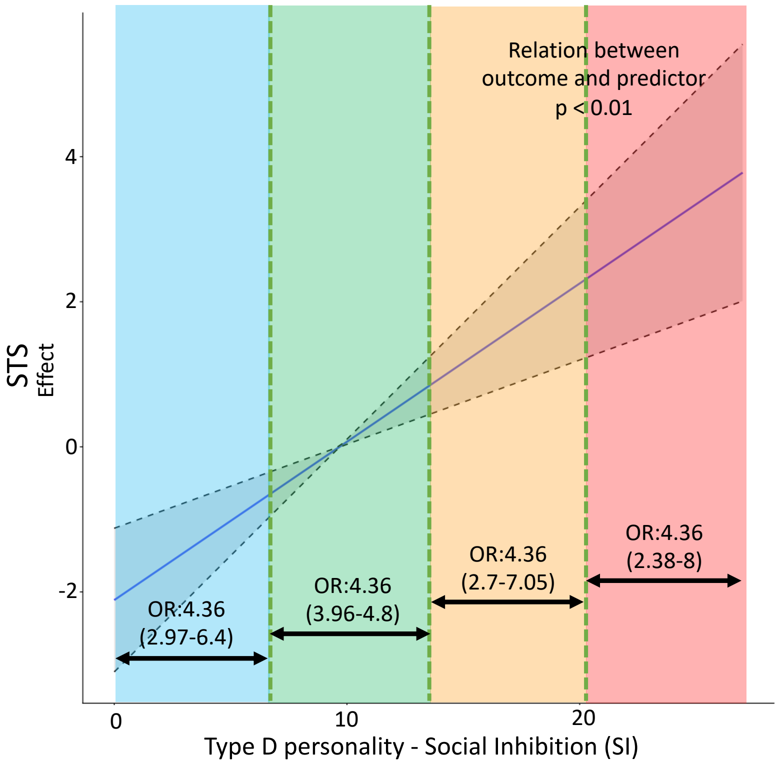

Figure 12: Multiple regression model of Type D personality - Social Inhibition (SI) for STS

Table 22: Result of Type D personality - Social Inhibition (SI) for STS in multiple regression

Variables	Odds ratio	95% CI	p-value
Age	GAM modeling: negative association		0.1
Sex	0.24	(0.07-0.83)	0.02
Marital status	1.57	(0.43-5.65)	0.49
Children	0.81	(0.33-1.99)	0.65
Exercise	0.54	(0.16-1.78)	0.31
Years of ICU experience	GAM modeling: negative association		0.07
Type D personality – Social Inhibition (SI)	GAM modeling: positive association<br>0th-25th 4.36<br>25th-50th 4.36<br>50th-75th 4.36<br>75th-100th 4.36	 (2.97-6.4)<br>(3.96-4.8)<br>(2.7-7.05)<br>(2.38-8)	<0.01

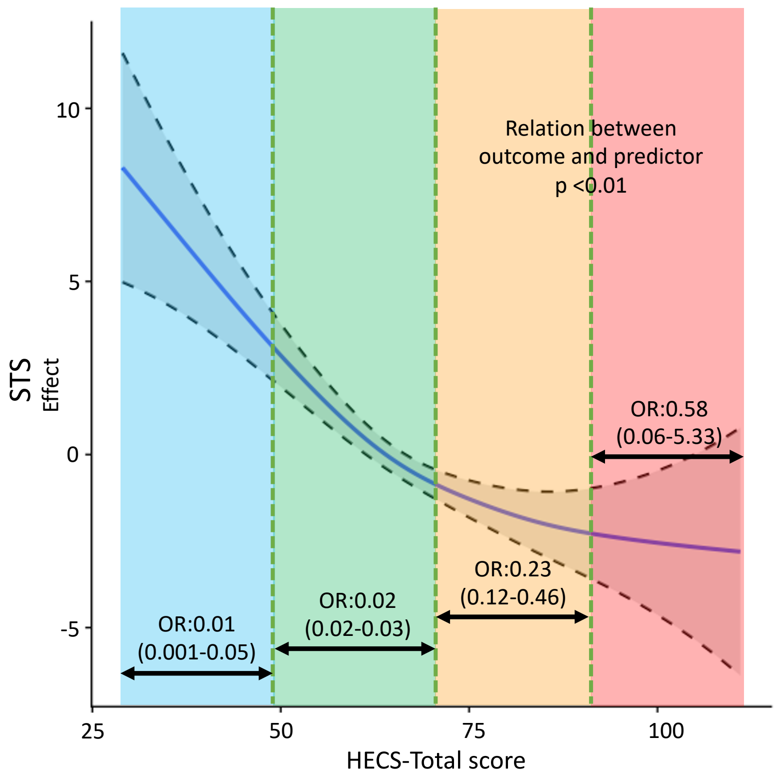

Figure 13: Multiple regression model of HECS-Total for STS

Table:23 Result of HECS –Hospital for STS in multiple regression

Variables	Odds ratio	95% CI	p-value
Age	GAM modeling: negative association		0.49
Sex	0.32	(0.09-1.05)	0.06
Marital status	1.25	(0.36-4.25)	0.71
Children	0.97	(0.41-2.28)	0.95
Exercise	0.67	(0.21-2.11)	0.5
Years of ICU experience	GAM modeling: negative association		0.23
HECS-Total	GAM modeling: negative association<br>0th-25th 0.01 (0.001-0.05)<br>25th-50th 0.02 (0.02-0.03)<br>50th -75th 0.23 (0.12-0.46)<br>75th-100th 0.58 (0.06-5.33)		<0.01

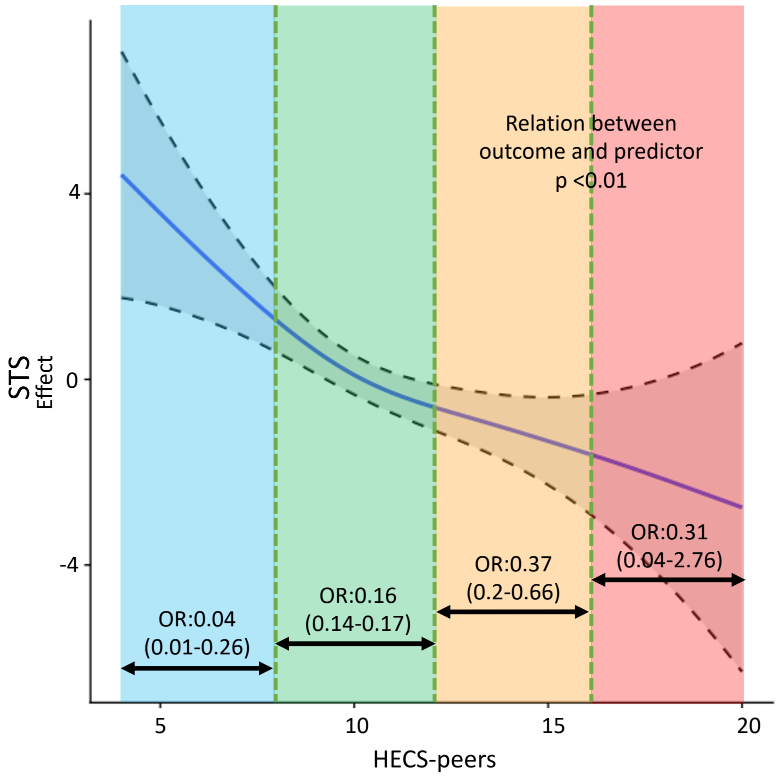

Figure 14: Multiple regression model of HECS- Peers for STS

Table 24: Result of HECS –Peers for STS in multiple regression

**Variables**	**Odds ratio**	**95% CI**	**p-value**
Age	GAM modeling: negative association		0.41
Sex	0.32	(0.09-1.1)	0.07
Marital status	1.38	(0.38-5)	0.61
Children	0.85	(0.34-2.08)	0.73
Exercise	0.57	(0.21-2.11)	0.36
Years of ICU experience	GAM modeling: negative association		0.06
HECS-Peers	GAM modeling: negative association<br>0th-25th 0.04<br>25th-50th 0.16<br>50th -75th 0.37<br>75th-100th 0.31	 (0.01-0.26)<br>(0.14-0.17)<br>(0.2-0.66)<br>(0.04-2.76)	<0.01

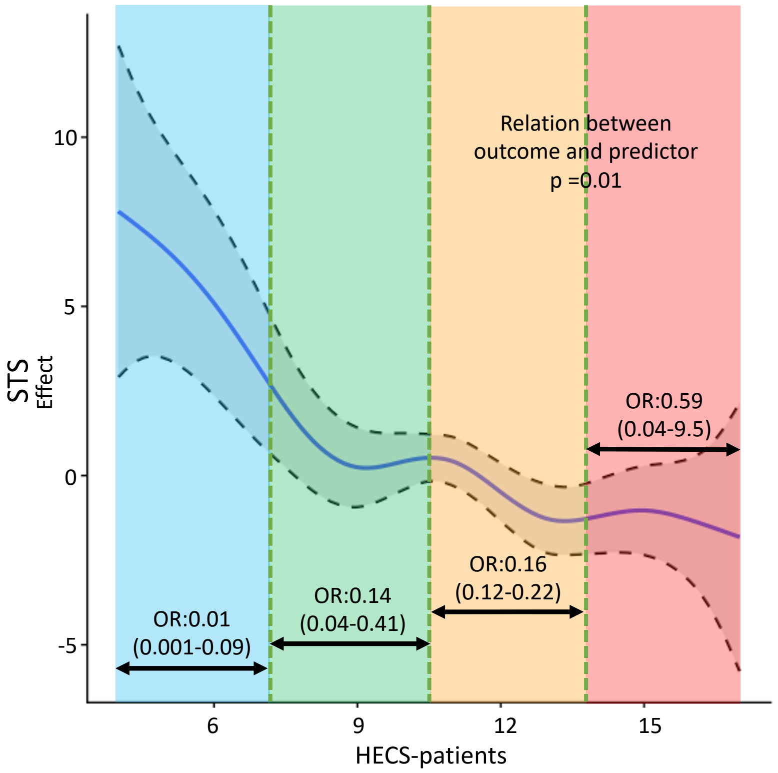

Figure 15: Multiple regression model of HECS-Patients for STS

Table 25: Result of HECS –Patients for STS in multiple regression

Variables	Odds ratio	95% CI	p-value
Age	GAM modeling: negative association		0.41
Sex	0.28	(0.02-3.29)	0.04
Marital status	1.35	(0.37-4.92)	0.65
Children	1.05	(0.43-2.57)	0.9
Exercise	0.55	(0.16-1.81)	0.32
Years of ICU experience	GAM modeling: negative association		0.12
HECS –Patients	GAM modeling: negative association<br>0th-25th 0.01<br>25th-50th 0.14<br>50th -75th 0.16<br>75th-100th 0.59	 (0.001-0.09)<br>(0.04-0.41)<br>(0.12-0.22)<br>(0.04-9.5)	0.01

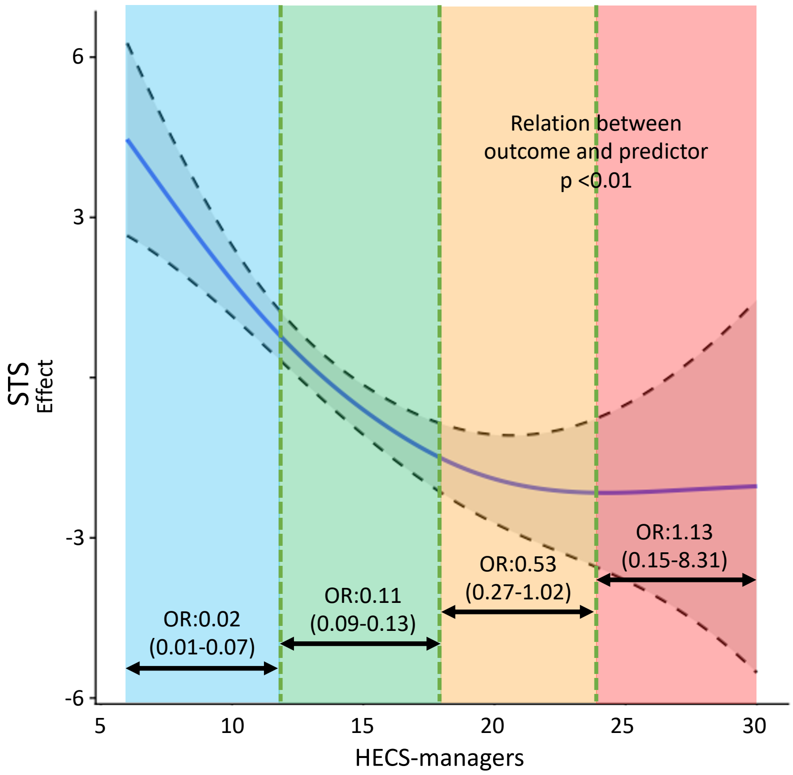

Figure 16: Multiple regression model of HECS- Managers for STS

Table 26: Result of HECS –Managers for STS in multiple regression

Variables	Odds ratio	95% CI	p-value
Age	GAM modeling: negative association		0.48
Sex	0.37	(0.05-2.55)	0.11
Marital status	1.33	(0.38-4.62)	0.64
Children	0.9	(0.38-2.15)	0.82
Exercise	0.49	(0.15-1.58)	0.23
Years of ICU experience	GAM modeling: negative association		0.09
HECS – Managers	GAM modeling: negative association<br>0th-25th 0.02<br>25th-50th 0.11<br>50th -75th 0.53<br>75th-100th 1.13	 (0.01-0.07)<br>(0.09-0.13)<br>(0.27-1.02)<br>(0.15-8.31)	<0.01

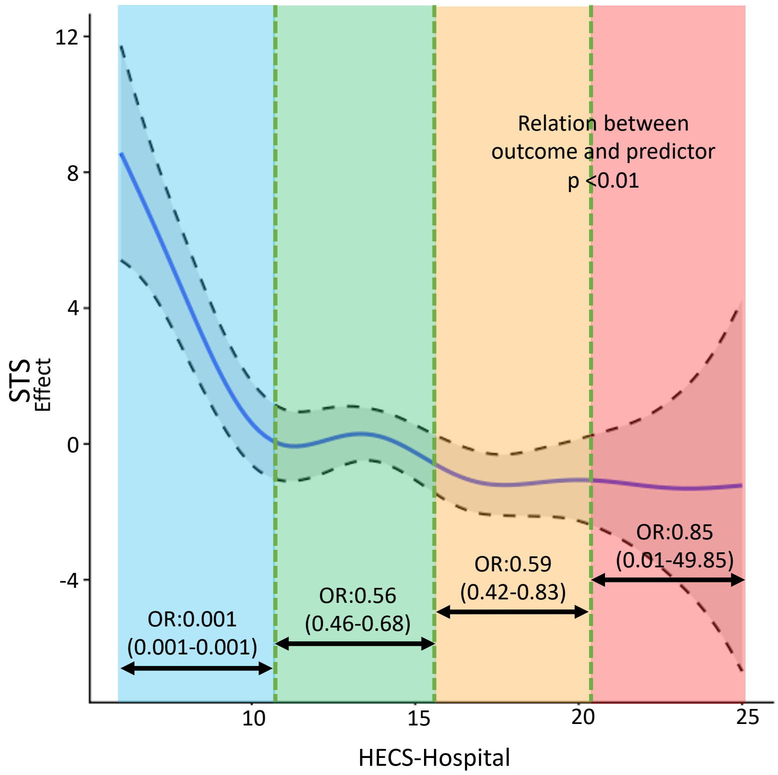

Figure 17: Multiple regression model of HECS- Hospital for STS

Table 27: Result of HECS –Hospital for STS in multiple regression

Variables	Odds ratio	95% CI	p-value
Age	GAM modeling: negative association		0.39
Sex	0.32	(0.09-1.06)	0.06
Marital status	1.27	(0.37-4.37)	0.69
Children	0.84	(0.35-1.98)	0.69
Exercise	0.6	(0.19-1.9)	0.38
Years of ICU experience	GAM modeling: negative association		0.18
HECS –Hospital	GAM modeling: negative association<br>0th-25th 0.001<br>25th-50th 0.56<br>50th-75th 0.59<br>75th-100th 0.85	 (0.001-0.001)<br>(0.46-0.68)<br>(0.42-0.83)<br>(0.01-49.85)	<0.01

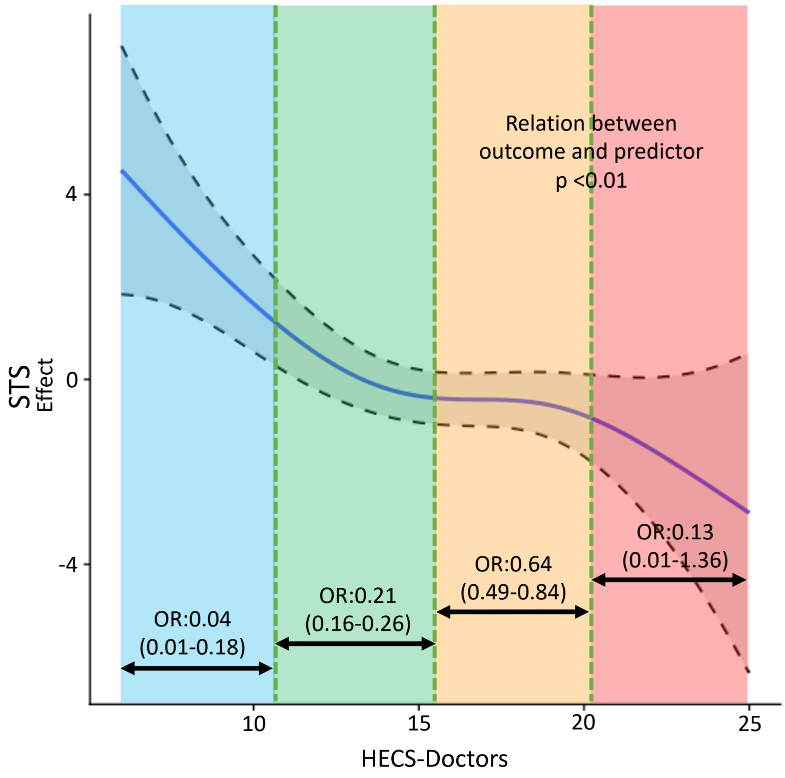

Figure 18: Multiple regression model of HECS- Doctors for STS

Table 28: Result of HECS –Doctors for STS in multiple regression

Variables	Odds ratio	95% CI	p-value
Age	GAM modeling: negative association		0.49
Sex	0.24	(0.07-0.83)	0.02
Marital status	1.19	(0.33-4.29)	0.79
Children	1	(0.41-2.46)	0.98
Exercise	0.71	(0.21-2.39)	0.59
Years of ICU experience	GAM modeling: negative association		0.23
HECS – Doctors	GAM modeling: negative association<br>0th-25th 0.04<br>25th-50th 0.21<br>50th-75th 0.64<br>75th-100th 0.13	 (0.01-0.18)<br>(0.16-0.26)<br>(0.49-0.84)<br>(0.01-1.36)	<0.01

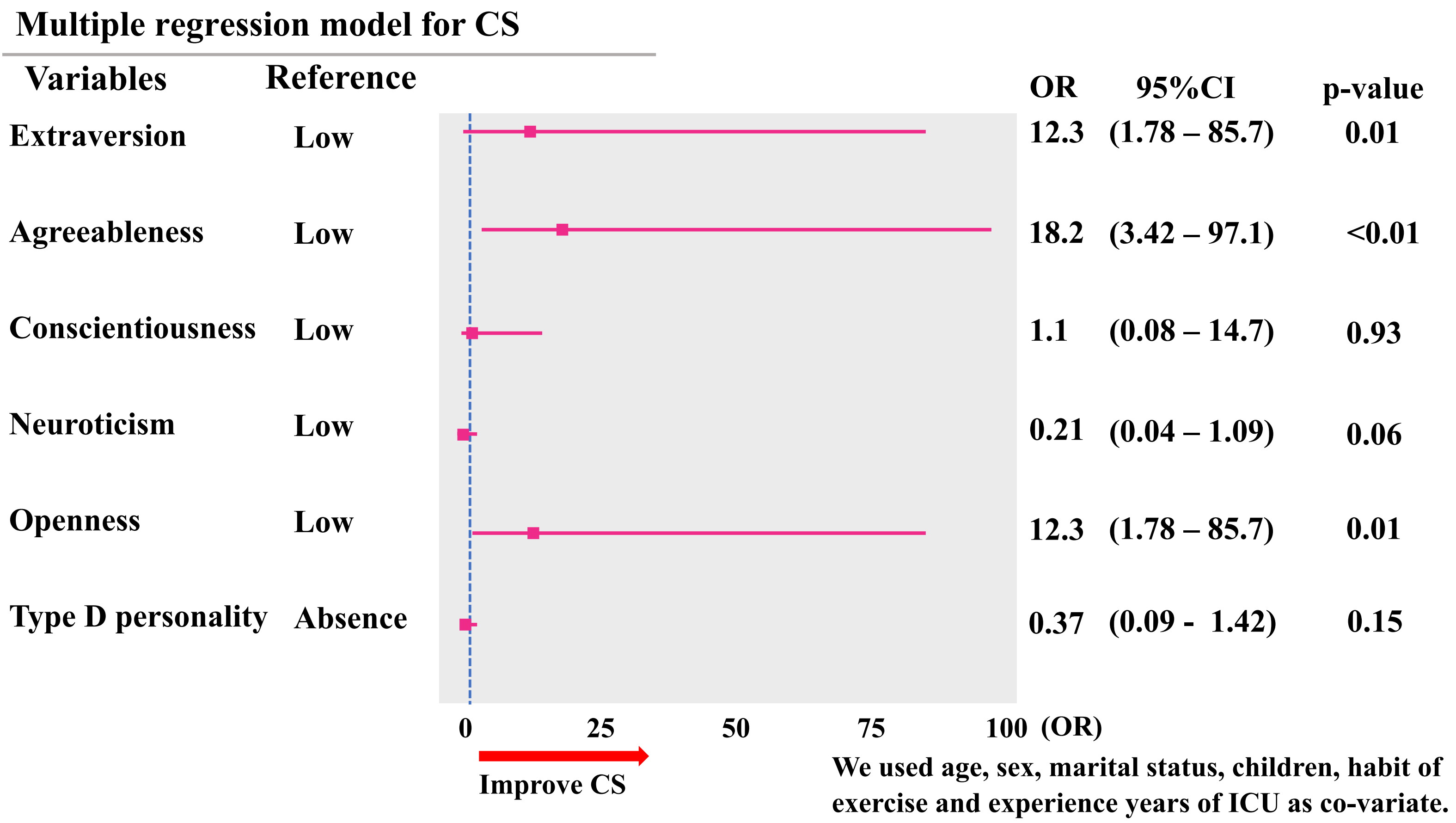

Figure 19: Multiple regression model of personality trait for CS

Table 29: Result of extraversion for CS in multiple regression

Variables	Odds ratio	95% CI	p-value
Age	GAM modeling: negative association		0.22
Sex	0.8	(0.18-3.54)	0.76
Marital status	0.23	(0.05-1.09)	0.06
Children	2.31	(0.78-6.79)	0.12
Exercise	11.55	(2.7-49.2)	<0.01
Years of ICU experience	GAM modeling: negative association		0.07
Extraversion	12.3	(1.78-85.7)	0.01

Table 30: Result of Agreeableness for CS in multiple regression

Variables	Odds ratio	95% CI	p-value
Age	GAM modeling: negative association		0.16
Sex	0.57	(0.12-2.56)	0.46
Marital status	0.23	(0.05-1.08)	0.06
Children	2.49	(0.85-7.27)	0.09
Exercise	11.44	(2.71-48.21)	<0.01
Years of ICU experience	GAM modeling: negative association		0.07
Agreeableness	18.2	(3.42-97.1)	<0.01

Table 31: Result of Conscientiousness for CS in multiple regression

Variables	Odds ratio	95% CI	p-value
Age	GAM modeling: negative association		0.12
Sex	0.91	(0.2-4.11)	0.91
Marital status	0.25	(0.05-1.23)	0.09
Children	2.41	(0.81-7.18)	0.11
Exercise	1.11	(0.25-4.82)	<0.01
Years of ICU experience	GAM modeling: negative association		0.14
conscientiousness	1.1	(0.08-14.7)	0.93

Table 32: Result of Neuroticism for CS in multiple regression

Variables	Odds ratio	95% CI	p-value
Age	GAM modeling: negative association		0.12
Sex	0.87	(0.19-3.89)	0.86
Marital status	0.25	(0.05-1.17)	0.08
Children	2.27	(0.76-6.72)	0.13
Exercise	11.03	(2.54-47.8)	<0.01
Years of ICU experience	GAM modeling: negative association		0.11
Neuroticism	0.21	(0.04-1.09)	0.06

Table 33: Result of Openness for CS in multiple regression

Variables	Odds ratio	95% CI	p-value
Age	GAM modeling: negative association		0.22
Sex	0.8	(0.18-3.54)	0.76
Marital status	0.23	(0.05-1.09)	0.06
Children	2.31	(0.78-6.79)	0.12
Exercise	11.55	(2.7-49.24)	<0.01
Years of ICU experience	GAM modeling: negative association		0.07
Openness	12.3	(1.78-85.7)	0.01

Table 34: Result of Type-D personality for CS in multiple regression

Variables	Odds ratio	95% CI	p-value
Age	GAM modeling: negative association		0.09
Sex	0.87	(0.19-3.92)	0.86
Marital status	0.25	(0.05-1.19)	0.08
Children	2.41	(0.81-7.15)	0.11
Exercise	13.49	(3.14-57.83)	<0.01
Years of ICU experience	GAM modeling: negative association		0.14
Type-D personality	0.37	(0.09-1.42)	0.15

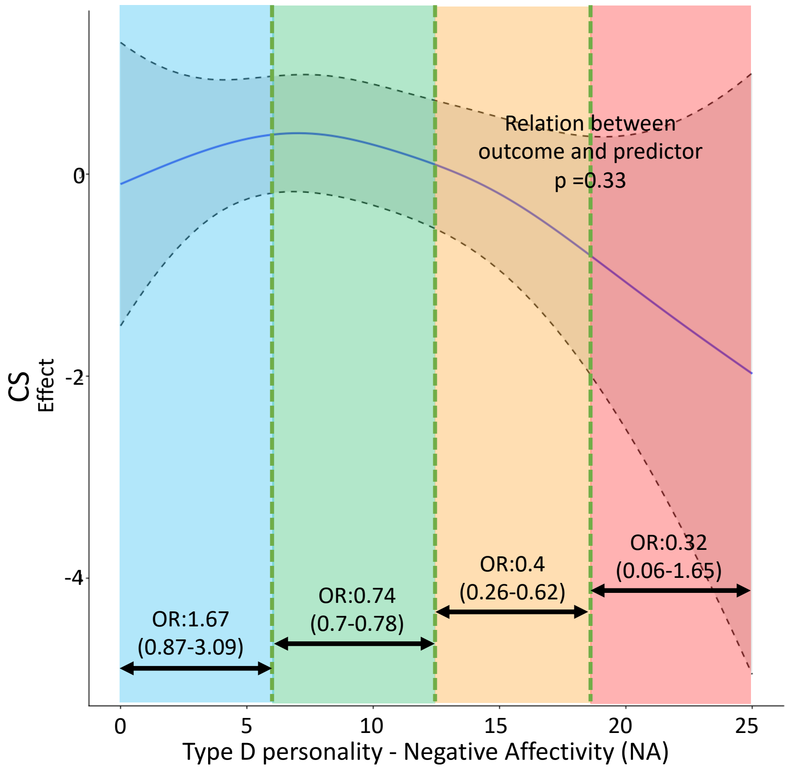

Figure 20: Multiple regression model of Type D personality - Negative Affectivity (NA) for CS

Table 35: Result of Type D personality - Negative Affectivity (NA) for CS in multiple regression

Variables	Odds ratio	95% CI	p-value
Age	GAM modeling: negative association		0.12
Sex	0.85	(0.19-3.8)	0.83
Marital status	0.23	(0.04-1.1)	0.06
Children	2.69	(0.89-8.09)	0.07
Exercise	13.39	(3.12-57.47)	<0.01
Years of ICU experience	GAM modeling: negative association		0.14
Type D personality-Negative Affectivity (NA)	GAM modeling: negative association		0.33
	0th-25th 1.67	(0.87-3.09)	
	25th-50th 0.74	(0.7-0.78)	
	50th-75th 0.4	(0.26-0.62)	
	75th-100th 0.32	(0.06-1.65)	

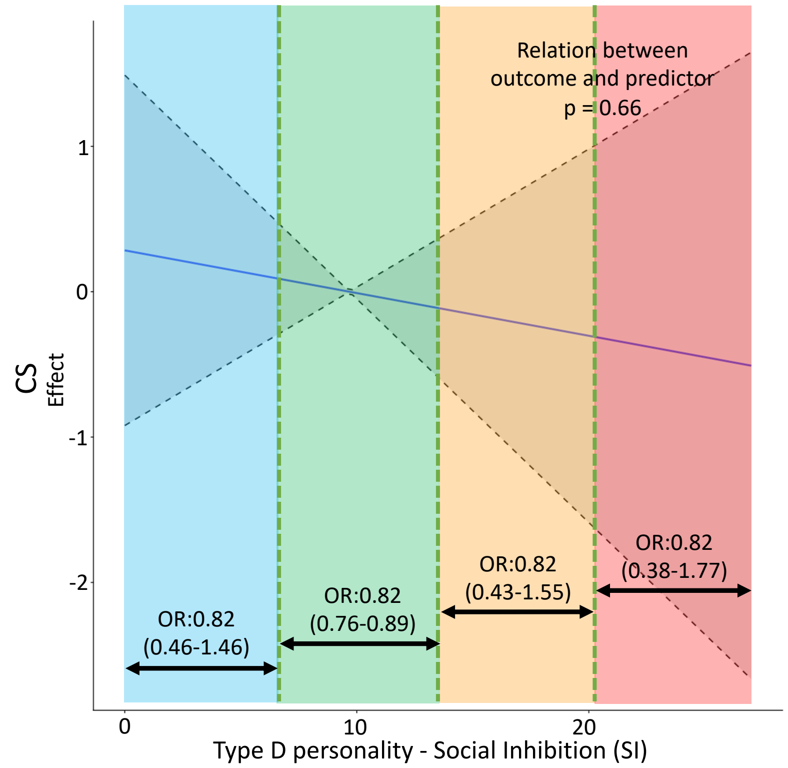

Figure 21: Multiple regression model of Type D personality - Social Inhibition (SI) for CS

Table 36: Result of Type D personality - Social Inhibition (SI) for CS in multiple regression

**Variables**	**Odds ratio**	**95% CI**	**p-value**
Age	GAM modeling: negative association		0.1
Sex	0.93	(0.2-4.18)	0.92
Marital status	0.25	(0.05-1.19)	0.08
Children	2.45	(0.82-7.3)	0.1
Exercise	13.46	(3.12-57.95)	<0.01
Years of ICU experience	GAM modeling: negative association		0.15
Type D personality – Social Inhibition (SI)	GAM modeling: negative association<br>0th-25th 0.82<br>25th-50th 0.82<br>50th -75th 0.82<br>75th-100th 0.82	 (0.46-1.46)<br>(0.76-0.89)<br>(0.43-1.55)<br>(0.38-1.77)	0.63

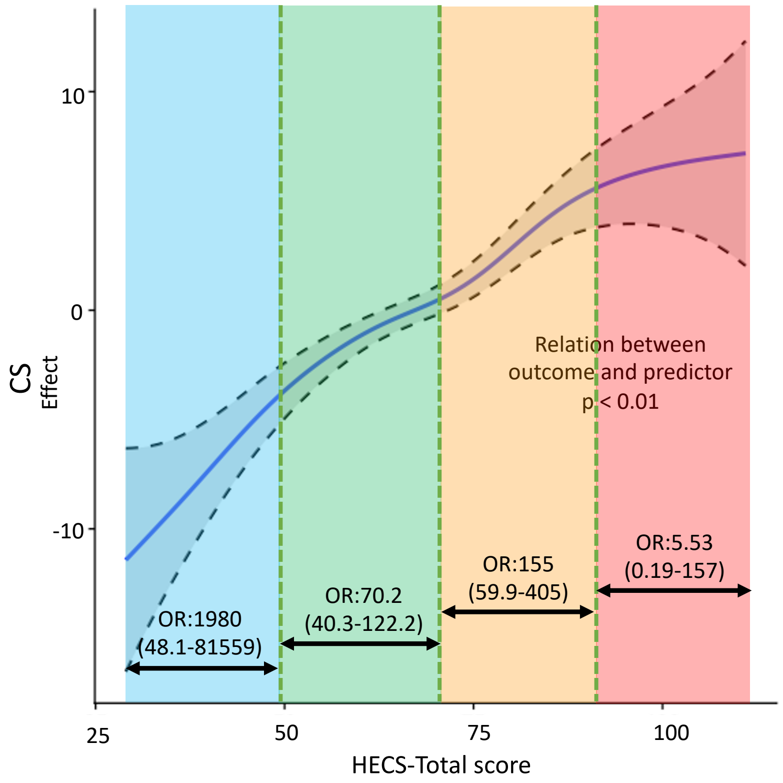

Figure 22: Multiple regression model of HECS-Total for CS

Table 37: Result of HECS –Hospital for CS in multiple regression

Variables	Odds ratio	95% CI	p-value
Age	GAM modeling: negative association		0.23
Sex	0.63	(0.16-2.37)	0.49
Marital status	0.3	(0.07-1.19)	0.69
Children	2.14	(0.82-5.57)	0.11
Exercise	9.52	(2.62-34.59)	<0.01
Years of ICU experience	GAM modeling: negative association		0.18
HECS-Total	GAM modeling: positive association<br>0th-25th 1980<br>25th-50th 70.2<br>50th -75th 155<br>75th-100t 5.53	 (48.1-81559)<br>(40.3-122.2)<br>(59.9-405)<br>(0.19-157)	<0.01

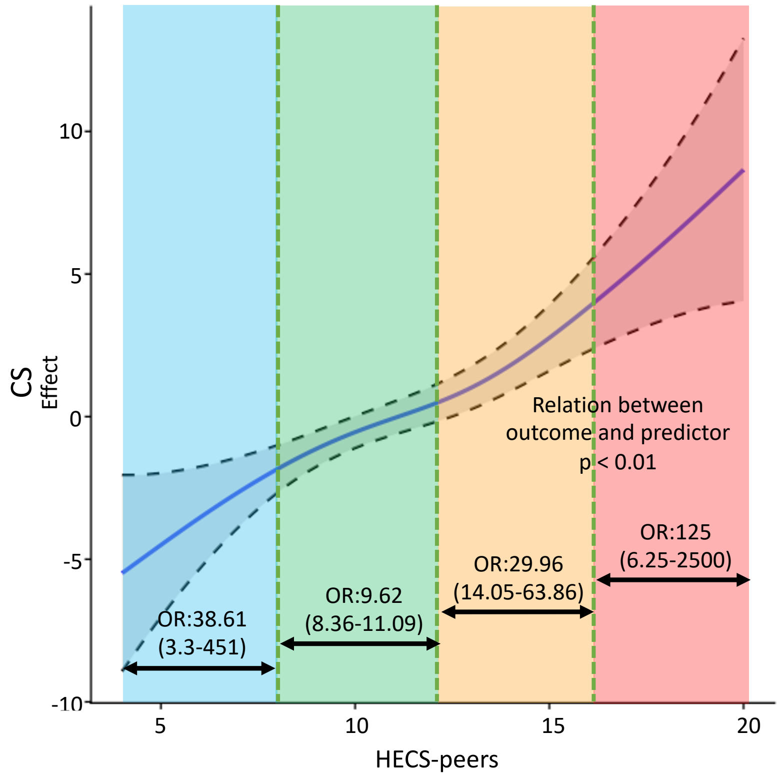

Figure 23: Multiple regression model of HECS-Peers for CS

Table 38: Result of HECS –Peers for CS in multiple regression

Variables	Odds ratio	95% CI	p-value
Age	GAM modeling: negative association		0.27
Sex	0.61	(0.14-2.53)	0.49
Marital status	0.22	(0.05-0.99)	0.04
Children	2.82	(1-7.88)	0.04
Exercise	9.86	(2.46-39.5)	<0.01
Years of ICU experience	GAM modeling: negative association		0.04
HECS-Peers	GAM modeling: positive association<br>0th-25th 38.61<br>25th-50th 9.62<br>50th-75th 29.96<br>75th-100th 125	 (3.3-451)<br>(8.36-11.09)<br>(14.05-63.86)<br>(6.25-2500)	<0.01

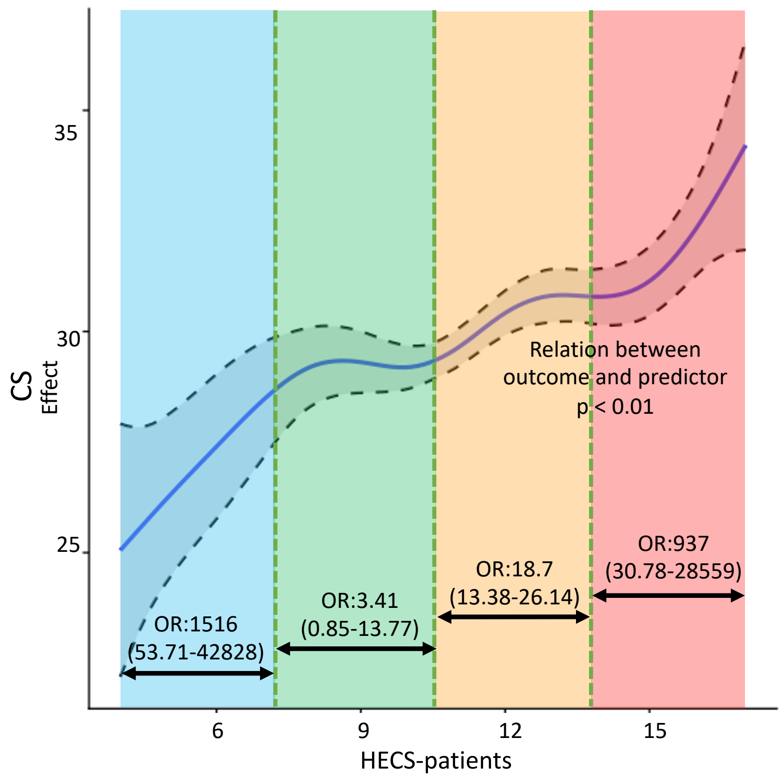

Figure 24: Multiple regression model of HECS-Patients for CS

Table 39: Result of HECS –Patients for CS in multiple regression

Variables	Odds ratio	95% CI	p-value
Age	GAM modeling: negative association		0.14
Sex	0.83	(0.2-3.34)	0.79
Marital status	0.77	(0.04-0.77)	0.02
Children	2.22	(0.8-6.17)	0.12
Exercise	11.9	(3.05-46.5)	<0.01
Years of ICU experience	GAM modeling: negative association		0.04
HECS –Patients	GAM modeling: positive association<br>0th-25th 1516<br>25th-50th 3.41<br>50th-75th 18.7<br>75th-100th 937	 (53.71-42828)<br>(0.85-13.77)<br>(13.38-26.14)<br>(30.78-28559)	<0.01

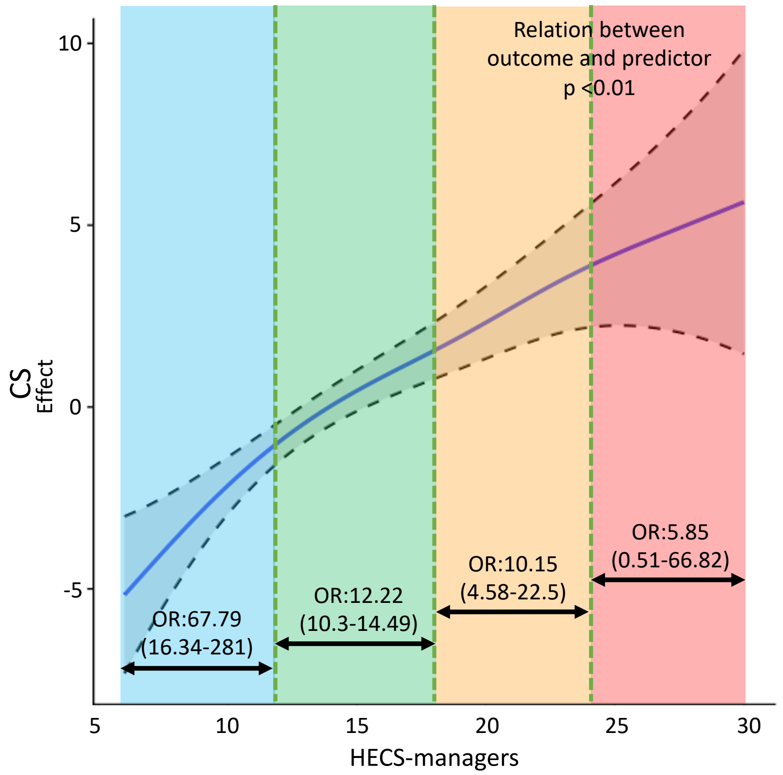

Figure 25: Multiple regression model of HECS- Managers for CS

Table 40: Result of HECS –Managers for CS in multiple regression

Variables	Odds ratio	95% CI	p-value
Age	GAM modeling: negative association		0.24
Sex	0.56	(0.13-2.32)	0.42
Marital status	0.27	(0.06-1.19)	0.08
Children	2.31	(0.83-6.39)	0.1
Exercise	13.4	(3.39-53.16)	<0.01
Years of ICU experience	GAM modeling: negative association		0.1
HECS – Managers	GAM modeling: positive association<br>0th-25th 67.79<br>25th-50th 12.22<br>50th -75th 10.15<br>75th-100th 5.85	 (16.34-281)<br>(10.3-14.49)<br>(4.58-22.5)<br>(0.51-66.82)	<0.01

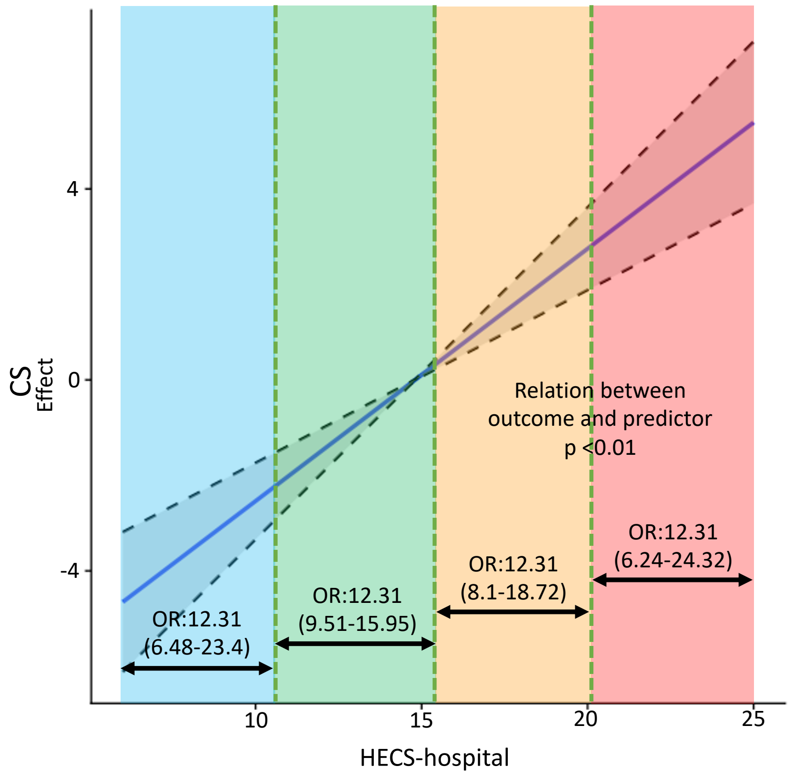

Figure 26: Multiple regression model of HECS- Hospital for CS

Table 41: Result of HECS –Hospital for CS in multiple regression

Variables	Odds ratio	95% CI	p-value
Age	GAM modeling: negative association		0.17
Sex	0.68	(0.16-2.81)	0.6
Marital status	0.27	(0.06-1.16)	0.08
Children	2.45	(0.88-6.81)	0.08
Exercise	11.1	(2.84-44.16)	<0.01
Years of ICU experience	GAM modeling: negative association		0.2
HECS –Hospital	GAM modeling: positive association		<0.01
	0th-25th 12.31	(6.48-23.4)	
	25th-50th 12.31	(9.51-15.95)	
	50th -75th 12.31	(8.1-18.72)	
	75th-100th 12.31	(6.24-24.32)	

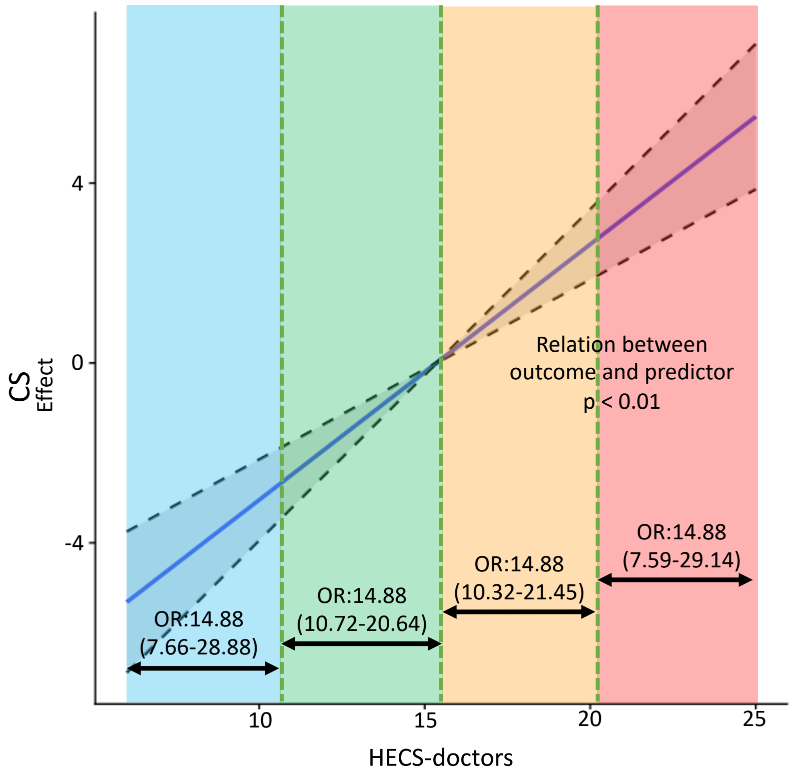

Figure 27: Multiple regression model of HECS- Doctors for CS

Table 42: Result of HECS –Doctors for CS in multiple regression

Variables	Odds ratio	95% CI	p-value
Age	GAM modeling: negative association		0.14
Sex	0.24	(0.07-0.83)	0.8
Marital status	1.19	(0.33-4.29)	0.12
Children	1	(0.41-2.46)	0.27
Exercise	0.71	(0.21-2.39)	<0.01
Years of ICU experience	GAM modeling: negative association		0.34
HECS – Doctors	GAM modeling: positive association<br>0th-25th 14.88<br>25th-50th 14.88<br>50th -75th 14.88<br>75th-100th 14.88	 (7.66-28.88)<br>(10.72-20.64)<br>(10.32-21.45)<br>(7.59-29.14)	<0.01

Figure legends

Figure 1: Multiple regression model of personality trait for BOS

This figure shows the result of personality trait for burnout syndrome (BOS) in multiple regression model. Odds ratio (OR) over 1.0 indicate that personality trait worsens BOS.

Figure 2: Multiple regression model of Type D personality - Negative Affectivity (NA) for BOS

This figure shows the result of Type D personality - Negative Affectivity (NA) for Burnout syndrome (BOS) in multiple regression model. We used age, sex, marital status, children, habit of exercise and experience years of ICU as co-variate. Relation between Type D personality – NA and BOS were significantly associated ($p<0.01$).

Figure 3: Multiple regression model of Type D personality -Social Inhibition (SI) for BOS

This figure shows the result of Type D personality - N Social Inhibition (SI) for Burnout syndrome (BOS) in multiple regression model. We used age, sex, marital status, children, habit of exercise and experience years of ICU as co-variate. Relation between Type D personality - SI and BOS were significantly associated ($p=0.01$).

Figure 4: Multiple regression model of HECS-Total for BOS

This figure shows the result of Hospital Ethical Climate Survey (HECS) -Total for Burnout syndrome (BOS) in multiple regression model. We used age, sex, marital status, children, habit of exercise and experience years of ICU as co-variate. Relation between HECS-Total and BOS were significantly associated ($p<0.01$).

Figure 5: Multiple regression model of HECS- Peers for BOS

This figure shows the result of Hospital Ethical Climate Survey (HECS) - Peers for Burnout syndrome (BOS) in multiple regression model. We used age, sex, marital status, children, habit of exercise and experience years of ICU as co-variate. Relation between HECS- Peers and BOS were not significantly associated ($p=0.07$).

Figure 6: Multiple regression model of HECS- Patients for BOS

This figure shows the result of Hospital Ethical Climate Survey (HECS) - Patients for Burnout syndrome (BOS) in multiple regression model. We used age, sex, marital status, children, habit of exercise and experience years of ICU as co-variate. Relation between HECS- Patients and BOS were significantly associated ($p<0.01$).

Figure 7: Multiple regression model of HECS- Managers for BOS

This figure shows the result of Hospital Ethical Climate Survey (HECS) - Managers for Burnout syndrome (BOS) in multiple regression model. We used age, sex, marital status, children, habit of exercise and experience years of ICU as co-variate. Relation between HECS- Managers and BOS were significantly associated ($p<0.01$).

Figure 8: Multiple regression model of HECS- Hospital for BOS

This figure shows the result of Hospital Ethical Climate Survey (HECS) - Hospital for Burnout syndrome (BOS) in multiple regression model. We used age, sex, marital status, children, habit of exercise and experience years of ICU as co-variate. Relation between HECS- Hospital and BOS were significantly associated ($p<0.01$).

Figure 9: Multiple regression model of HECS- Doctors for BOS

This figure shows the result of Hospital Ethical Climate Survey (HECS) - Doctors for Burnout syndrome (BOS) in multiple regression model. We used age, sex, marital status, children, habit of exercise and experience years of ICU as co-variate. Relation between HECS- Doctors and BOS were significantly associated ($p<0.01$).

Figure 10: Multiple regression model of personality trait for STS

This figure shows the result of personality trait for secondary traumatic stress (STS) in multiple regression model. Odds ratio (OR) over 1.0 indicate that personality trait worsens STS.

Figure 11: Multiple regression model of Type D personality - Negative Affectivity (NA) for STS

This figure shows the result of Type D personality - Negative Affectivity (NA) for secondary traumatic stress (STS) in multiple regression model. We used age, sex, marital status, children, habit of exercise and experience years of ICU as co-variate. Relation between Type D personality – NA and STS were significantly associated ($p=0.01$).

Figure 12: Multiple regression model of Type D personality -Social Inhibition (SI) for STS

This figure shows the result of Type D personality - N Social Inhibition (SI) for secondary traumatic stress (STS) in multiple regression model. We used age, sex, marital status, children, habit of exercise and experience years of ICU as co-variate. Relation between Type D personality - SI and STS were significantly associated ($p<0.01$).

Figure 13: Multiple regression model of HECS-Total for STS

This figure shows the result of Hospital Ethical Climate Survey (HECS) -Total for secondary traumatic stress (STS) in multiple regression model. We used age, sex, marital status, children, habit of exercise and experience years of ICU as co-variate. Relation between HECS-Total and STS were significantly associated (p<0.01).

Figure 14: Multiple regression model of HECS- Peers for STS

This figure shows the result of Hospital Ethical Climate Survey (HECS) - Peers for secondary traumatic stress (STS) in multiple regression model. We used age, sex, marital status, children, habit of exercise and experience years of ICU as co-variate. Relation between HECS- Peers and STS were significantly associated (p<0.01).

Figure 15: Multiple regression model of HECS- Patients for STS

This figure shows the result of Hospital Ethical Climate Survey (HECS) - Patients for secondary traumatic stress (STS) in multiple regression model. We used age, sex, marital status, children, habit of exercise and experience years of ICU as co-variate. Relation between HECS- Patients and STS were significantly associated (p=0.01).

Figure 16: Multiple regression model of HECS- Managers for STS

This figure shows the result of Hospital Ethical Climate Survey (HECS) - Managers for secondary traumatic stress (STS) in multiple regression model. We used age, sex, marital status, children, habit of exercise and experience years of ICU as co-variate. Relation between HECS-Managers and STS were significantly associated (p<0.01).

Figure 17: Multiple regression model of HECS- Hospital for STS

This figure shows the result of Hospital Ethical Climate Survey (HECS) - Hospital for secondary traumatic stress (STS) in multiple regression model. We used age, sex, marital status, children, habit of exercise and experience years of ICU as co-variate. Relation between HECS- Hospital and STS were significantly associated (p<0.01).

Figure 18: Multiple regression model of HECS- Doctors for STS

This figure shows the result of Hospital Ethical Climate Survey (HECS) - Doctors for secondary traumatic stress (STS) in multiple regression model. We used age, sex, marital status, children, habit of exercise and experience years of ICU as co-variate. Relation between HECS- Doctors and STS were significantly associated (p<0.01).

Figure 19: Multiple regression model of personality trait for CS
This figure shows the result of personality trait for compassion satisfaction (CS) in multiple regression model. Odds ratio (OR) over 1.0 indicate that personality trait improve CS.

Figure 20: Multiple regression model of Type D personality - Negative Affectivity (NA) for CS
This figure shows the result of Type D personality - Negative Affectivity (NA) for compassion satisfaction (CS) in multiple regression model. We used age, sex, marital status, children, habit of exercise and experience years of ICU as co-variate. Relation between Type D personality – NA and CS were not significantly associated (p=0.33).

Figure 21: Multiple regression model of Type D personality -Social Inhibition (SI) for CS
This figure shows the result of Type D personality - N Social Inhibition (SI) for compassion satisfaction (CS) in multiple regression model. We used age, sex, marital status, children, habit of exercise and experience years of ICU as co-variate. Relation between Type D personality - SI and CS were not significantly associated (p=0.66).

Figure 22: Multiple regression model of HECS-Total for CS
This figure shows the result of Hospital Ethical Climate Survey (HECS) -Total for compassion satisfaction (CS) in multiple regression model. We used age, sex, marital status, children, habit of exercise and experience years of ICU as co-variate. Relation between HECS-Total and CS were significantly associated (p<0.01).

Figure 23: Multiple regression model of HECS- Peers for CS
This figure shows the result of Hospital Ethical Climate Survey (HECS) - Peers for compassion satisfaction (CS) in multiple regression model. We used age, sex, marital status, children, habit of exercise and experience years of ICU as co-variate. Relation between HECS- Peers and CS were significantly associated (p<0.01).

Figure 24: Multiple regression model of HECS- Patients for CS
This figure shows the result of Hospital Ethical Climate Survey (HECS) - Patients for compassion satisfaction (CS) in multiple regression model. We used age, sex, marital status, children, habit of exercise and experience years of ICU as co-variate. Relation between HECS-Patients and CS were significantly associated (p<0.01).

Figure 25: Multiple regression model of HECS- Managers for CS

This figure shows the result of Hospital Ethical Climate Survey (HECS) - Managers for compassion satisfaction (CS) in multiple regression model. We used age, sex, marital status, children, habit of exercise and experience years of ICU as co-variate. Relation between HECS-Managers and CS were significantly associated ($p<0.01$).

Figure 26: Multiple regression model of HECS- Hospital for CS

This figure shows the result of Hospital Ethical Climate Survey (HECS) - Hospital for compassion satisfaction (CS) in multiple regression model. We used age, sex, marital status, children, habit of exercise and experience years of ICU as co-variate. Relation between HECS-Hospital and CS were significantly associated ($p<0.01$).

Figure 27: Multiple regression model of HECS- Doctors for CS

This figure shows the result of Hospital Ethical Climate Survey (HECS) - Doctors for compassion satisfaction (CS) in multiple regression model. We used age, sex, marital status, children, habit of exercise and experience years of ICU as co-variate. Relation between HECS-Doctors and CS were significantly associated ($p<0.01$).
